# Coordination of ε-Caprolactone to a Cationic Niobium(V) Alkoxide Complex: Fundamental Insight into Ring-Opening Polymerization via Coordination–Insertion

**DOI:** 10.1021/acs.inorgchem.3c02491

**Published:** 2023-09-11

**Authors:** Antoine Buchard, Matthew G. Davidson, Gerrit Gobius du Sart, Matthew D. Jones, Gabriele Kociok-Köhn, Strachan N. McCormick, Paul Mckeown

**Affiliations:** † Institute for Sustainability, University of Bath, Bath BA2 7AY, U.K.; ‡ Department of Chemistry, University of Bath, Bath BA2 7AY, U.K.; § TotalEnergies Corbion, Stadhuisplein 70, Gorinchem 4203 NS, The Netherlands; ∥ Material and Chemical Characterization and Analysis Facility (MC2), University of Bath, Bath BA2 7AY, U.K.

## Abstract

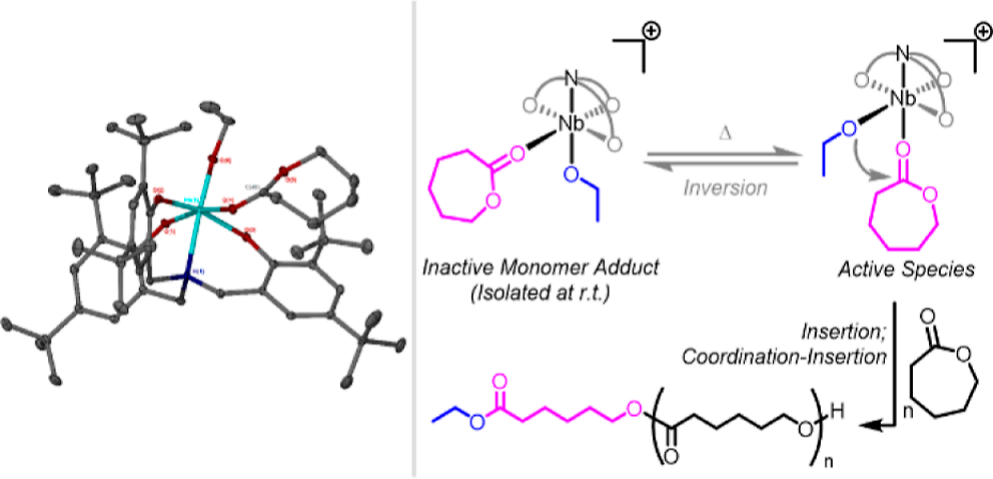

We report three niobium-based initiators for the catalytic ring-opening polymerization (ROP) of ε-caprolactone, exhibiting good activity and molecular weight control. In particular, we have prepared on the gram-scale and fully characterized a monometallic cationic alkoxo-Nb(V) ε-caprolactone adduct representing, to the best of our knowledge, an unprecedented example of a metal complex with an intact lactone monomer and a functional ROP-initiating group simultaneously coordinated at the metal center. At 80 °C, all three systems initiate the immortal solution-state ROP of ε-caprolactone via a coordination–insertion mechanism, which has been confirmed through experimental studies, and is supported by computational data. Natural bond orbital calculations further indicate that polymerization may necessitate isomerization about the metal center between the alkoxide chain and the coordinated monomer. The observations made in this work are expected to inform mechanistic understanding both of amine tris(phenolate)-supported metal alkoxide ROP initiators, including various highly stereoselective systems for the polymerization of lactides and of coordination–insertion-type ROP protocols more broadly.

## Abstract 2

Cationic niobium(V) complexes are reported as catalysts for the controlled, immortal ring-opening polymerization of ε-caprolactone. In particular, gram-scale isolation of an unprecedented stable metal complex, simultaneously bearing a coordinated monomer molecule and an alkoxide initiating group, is facile at ambient temperature. Catalytic studies and mechanistic interrogation of this species and others have been undertaken.

## Introduction

As the environmental persistence of traditional plastics leads to widespread contamination of marine and terrestrial ecosystems, the development of compostable alternatives to established polyolefins and polyesters, with well-defined properties, becomes increasingly relevant.^1–3^ Biodegradable plastics such as poly(lactic acid) (PLA) hold potential for the production of food packaging and single-use items such as tea bags, coffee pods, and drinking cups.^4–6^ PLA, poly(ε-caprolactone) (PCL), and associated copolymers have also received attention regarding high-value biomedical and tissue engineering applications.^6–19^ For the industrial production of biodegradable polyesters, the catalytic, living or immortal, ring-opening polymerization (ROP) of cyclic esters is the preferred route, rather than polycondensation of the relevant hydroxy acid as high-molecular-weight polymers can be readily produced in a controlled manner and under relatively mild conditions.^6,20^ A living kinetic regime, in which the rate of initiation is much greater than that of propagation, ensures simultaneous or near-simultaneous growth of all polymer chains in the system. This yields a product of narrow molecular weight distribution (dispersity, *D̵*_M_), and controllable molecular weight, the degree of polymerization at quantitative conversion being inversely proportional to the concentration of initiating groups (catalyst loading).^1,5,6,21–25^

The ROP of cyclic esters is generally carried out in the presence of a catalytic quantity of a metal complex (although many organocatalytic systems have been reported)^26–30^ and proceeds via either a coordination–insertion^17,21,22,29,31–33^ or activated monomer pathway.^21,34–36^ The coordination–insertion mechanism is typically associated with metal alkoxide precatalysts. Therein, following coordination to the metal, the monomer inserts into the metal–alkoxide bond via nucleophilic attack by the alkoxide at the monomer carbonyl group, which is activated by the Lewis acid metal center. This is followed by ring-opening to yield a metal-coordinated growing chain, and further equivalents of the monomer can then insert into the resulting metal–alkoxide bond. In the case of an immortal system, the rate at which chains exchange between the active site of the catalytic species and an exogenous alcohol chain-transfer agent (nucleophile, co-initiator) far exceeds that of propagation. This permits controlled chain growth at substoichiometric concentrations of the catalyst relative to polymer chain ends, allowing the polymer molecular weight to be manipulated without altering the precatalyst loading, by instead varying the co-initiator concentration.^17,29,31,32^

Although widely understood to be the first intermediate of the coordination–insertion mechanism, to date there have been no reports of a metal complex simultaneously bearing both a coordinated lactone and an alkoxide or other initiating group. However, the literature contains several crystallographically characterized examples of ε-caprolactone coordinated to a metal.^37–42^ Most relevant to our work, Dagorne and co-workers prepared a ε-caprolactone adduct of a cationic methylaluminum monophenolate, elucidating its solid-state structure via X-ray diffraction. They also prepared an analogous ε-caprolactone adduct of a cationic isobutylaluminum monophenolate and a lactide adduct of a cationic methylaluminum monophenolate. However, solid-state structures of the two latter systems were not obtained.^41^ It was proposed that the ROP of ε-caprolactone initiated by a structurally related cationic alkoxoaluminum monophenolate precatalyst would proceed via a hypothetical alkoxoaluminum ε-caprolactone adduct intermediate, although that species was not observed. Similarly, Lewiński and co-workers reported a stable ε-caprolactone adduct of a neutral methylaluminum bis(phenolate), which initiates ROP on exposure to oxygen in the presence of excess ε-caprolactone.^43^ This was attributed to initial formation of the corresponding methoxide complex, although that species was not observed. Additionally, examples of lithium complexes of γ-butyrolactone and γ-valerolactone,^44^ and rhenium complexes of γ-butyrolactone and β-propiolactone,^45^ have been reported. However, those reports were not concerned with polymerization catalysis. Despite the importance of the coordination–insertion mechanism to the ROP of cyclic esters, none of the lactone complexes described in the literature bear any kind of functional initiating group.

We and others have previously reported *C*_3_-symmetric amine tris(phenolate)-supported monoalkoxide complexes of the tetravalent elements titanium, zirconium, hafnium, and germanium as initiators for the ROP of lactides.^46–48^ Our Zr and Hf systems combined high activity with remarkable heteroselectivity in the ROP of the racemic monomer (*P*_r_ = 0.88–0.98). Proceeding via a coordination–insertion mechanism, selectivity was then attributed to a dynamic enantiomorphic site control mechanism, relating to inversion of helical chirality of the *C*_3_-symmetric ligand scaffold.^46^ Notably, Kol and co-workers demonstrated that sterically demanding substituents ortho to the phenolate oxygen atoms of the ancillary ligand can realize in catalysts of this type exceptional activity and robustness in the ROP of l-lactide under challenging conditions.^48^ Moreover, our group subsequently reported a homoleptic Zr amine tris(phenolate)-based protocol employing a liquid catalyst formulation, that represents a credible alternative to established industrial approaches.^49,50^

Complexes of the heavier group five metals, niobium and tantalum, have received little attention as initiators for the ROP of cyclic esters, with only a small handful of reports in the literature.^14,51–57^ Indeed, the authors are aware of no established industrial catalytic processes mediated by Nb-based species, although this metal does have various specialist metallurgical uses,^58,59^ as well as having more recently garnered interest in relation to potential energy material applications.^60–62^ Importantly, ethical sourcing of Nb and Ta is a key consideration in developing any process for which they might be employed.^63–66^

In addition to a recent report from Plaman and Durr regarding the catalytic application of phenoxyimino Nb and Ta ethoxides,^56^ and Al-Khafaji and co-worker’s work encompassing the use of calixarene-supported NbCl_5_ as a ROP initiator,^57^ reports from the groups of Chakraborty and Redshaw concerning, respectively, the use of Nb and Ta imino phenoxides and tetraphenolate-supported Nb and Ta species,^51,55^ and a poorly controlled trihydridoniobocene system reported by Otero and co-workers,^54^ remain the only examples of these metals being successfully applied to the polymerization of ε-caprolactone. Several vanadium-catalyzed protocols have also been reported for the ROP of cyclic esters, including for ε-caprolactone.^67–70^

The groups of Kol and Verkade have both independently prepared a tantalum(V) bis(ethoxide) complex supported by a methyl-substituted amine tris(phenolate) ancillary ligand.^52,71^ Verkade and co-workers reported that pseudo-octahedral species is inactive for lactide polymerization.^52^ Furthermore, Kol and co-workers found that the alkoxide position cis to the amine donor could be selectively substituted in the presence of a halide source, chlorotrimethylsilane (TMSCl), with the alkoxide moiety trans to the amine group remaining remarkably unreactive.^71^ Kol and co-workers also prepared a Ta(V) bis(ethoxide) complex bearing a bulkier *tert*-butyl-substituted ligand scaffold, although chlorination of that species was not reported.^72^ Nonetheless, Nomura and co-workers described the selective triflation of a dichloro Nb(V) complex supported by that more sterically demanding ancillary ligand, at the position trans to the bridgehead nitrogen.^73^ Accordingly, it is apparent that a trans influence arising from the nitrogen atom of the amine tris(phenolate) scaffold confers a remarkable difference in the lability of otherwise identical monodentate ligands situated at the cis and trans positions, respectively, in complexes of this type.

Herein, we report the synthesis and full characterization of a range of novel niobium(V) complexes. Several species have been successfully applied to the catalytic ROP of ε-caprolactone, exhibiting good activity and molecular weight control, providing mechanistic insight into the coordination–insertion-type ROP of cyclic esters in the presence of metal alkoxide precatalysts, and in particular tetravalent amine tris(phenolate)-supported systems. Moreover, we report a stable monometallic cationic Nb(V) ε-caprolactone adduct that is, to the best of our knowledge, the first observed example of a metal complex with an intact lactone monomer and a functional initiating group simultaneously coordinated at the metal center.

## Results and Discussion

### Synthesis and Reactivity of Niobium(V) Complexes

**Scheme 1:**
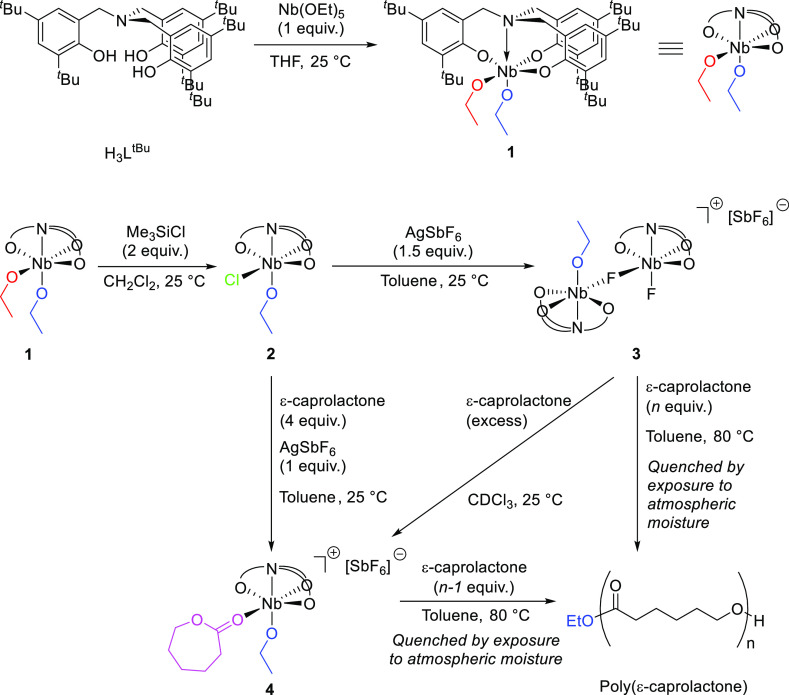
Synthesis and Reactivity of *tert*-Butyl-Substituted Complexes **1–4**

**Figure 1:**
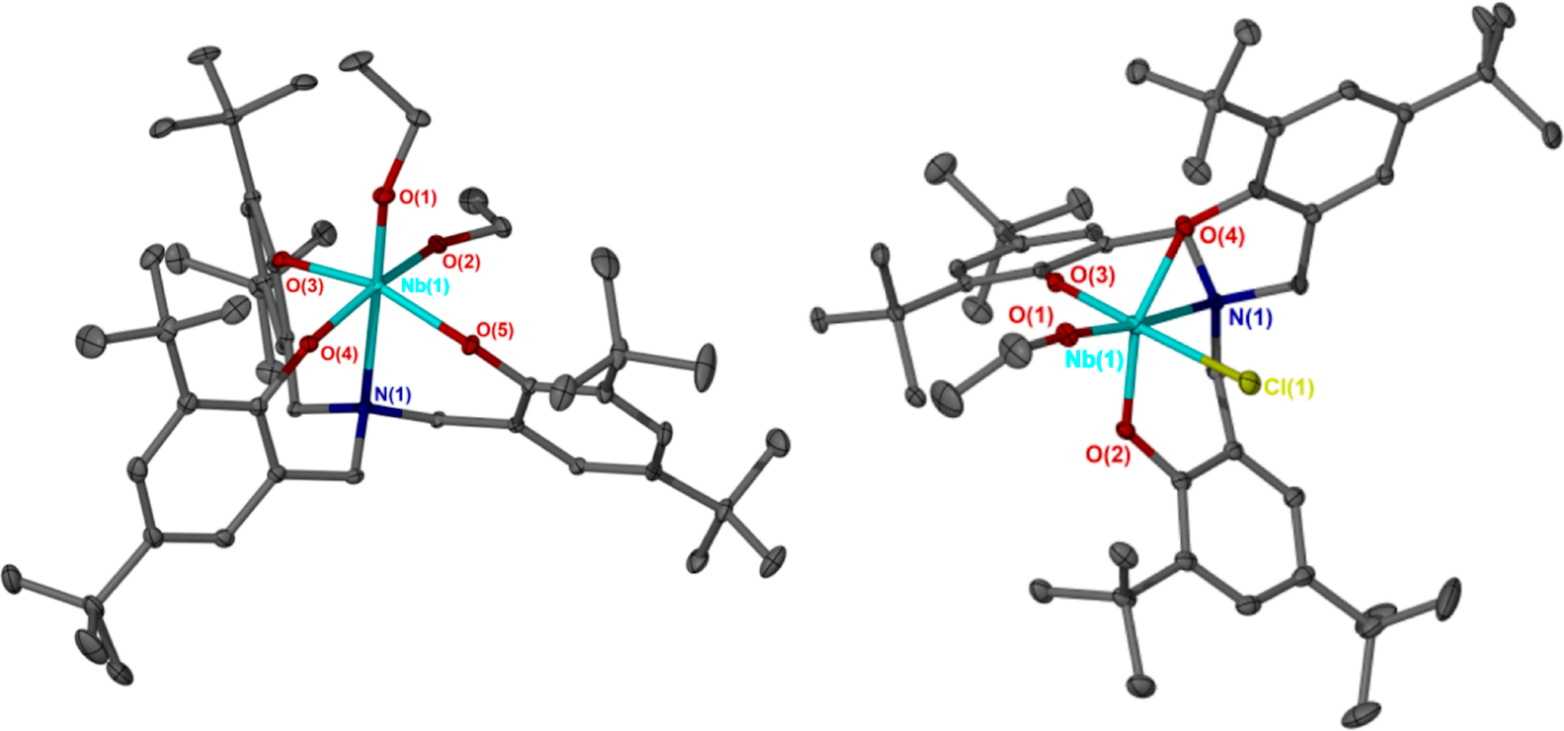
Solid-state structures of neutral Nb(V) complexes **1** (left) and **2** (right). Ellipsoids are shown at the 30% probability level. Hydrogen atoms and lattice solvent have been omitted for clarity. Selected bond lengths (Å) and angles (deg): **1** Nb(1)–O(1) 1.8737(16), Nb(1)–O(2) 1.8828(17), Nb(1)–N(1) 2.3765(17), N(1)–Nb(1)–O(1) 173.24(7), O(1)–Nb(1)–O(2) 97.40(7), O(1)–Nb(1)–O(3) 101.60(7), O(1)–Nb(1)–O(4) 93.40(7), O(1)–Nb(1)–O(5) 100.30(7), O(2)–Nb(1)–O(3) 87.04(7), O(2)–Nb(1)–O(5) 88.62(7), and O(2)–Nb(1)–N(1) 88.84(7). **2** Nb(1)–O(1) 1.8510(19), Nb(1)–Cl(1) 2.4203(7), Nb(1)–N(1) 2.371(2), N(1)–Nb(1)–O(1) 174.45(8), O(1)–Nb(1)–O(2) 100.82(8), O(1)–Nb(1)–O(3) 94.81(8), O(1)–Nb(1)–O(4) 100.17(8), O(1)–Nb(1)–Cl(1) 95.73(6), O(2)–Nb(1)–Cl(1) 83.86(5), O(4)–Nb(1)–Cl(1) 84.37(6), N(1)–Nb(1)–Cl(1) 89.12(5).

Treatment of niobium(V) ethoxide with pro-ligand tris(2-hydroxy-3,5-di-*tert*-butylbenzyl)amine, H_3_L^*t*Bu^, afforded the pseudo-octahedral bis(ethoxide) complex; [L^*t*Bu^Nb(OEt)_2_], **1** (Scheme 1 and Figure 1).

Characterization by single-crystal X-ray diffraction revealed **1** to be structurally similar to the Ta(V) bis(alkoxide) system, [L^Me^Ta(OEt)_2_], prepared by the groups of Kol and Verkade, using pro-ligand tris(2-hydroxy-3,5-dimethylbenzyl)amine, H_3_L^Me^.^52,71^ On reaction with excess TMSCl, **1** was selectively chlorinated at the position cis to the nitrogen moiety to yield [L^*t*Bu^Nb(OEt)Cl], **2** (Figure 1), exhibiting analogous reactivity to the Ta(V) bis(ethoxide) complex of (L^Me^)^3–^ reported by Kol.^71^ This is consistent with the discrepancy observed between the Nb–O bond lengths corresponding to the ethoxide positions cis and trans to the bridgehead nitrogen, respectively, of **1** (and of **5**, see the Supporting Information) in the solid state, attributable to a trans influence. Moreover, calculation of natural charges for **1**, vide infra, reveals significantly greater localization of negative charge on the oxygen atom of the cis alkoxide than on that in the trans position, consistent with the increased nucleophilicity of the former. However, the (L^Me^)^3–^ scaffolds of all relevant Ta(V) complexes in the literature have been consistently reported to be *C*_1_-symmetric about the Ta–N axis, the sterically demanding *tert*-butyl-substituted ligand systems of complexes **1** and **2** were found to be pseudo-*C*_3_-symmetric with respect to the conformation of the aromatic rings about the N–Nb bond, although the hexacoordinate Nb(V) centers remain *C*_1_-symmetric and pseudo-octahedral. NMR spectroscopic data and elemental (CHN) analyses confirmed that the solid-state structures of **1** and **2** were representative of the bulk material and were retained in solution. While consistent with the structure of Nb(V) triflate complex [L^*t*Bu^Nb(OTf)Cl], reported by Nomura and co-workers,^73^ the pseudo-*C*_3_ geometry of the ancillary ligand systems of **1** and **2** is in contrast to the *C*_1_-symmetric structure of bis(dimethylamido) complex [L^*t*Bu^Ta(NMe)]_2_ reported by Kol and co-workers.^72^ It is also therefore distinct from the proposed structure of the bis(ethoxide) complex [L^*t*Bu^Ta(OEt)]_2_, simultaneously reported by Kol, but not characterized in the solid state.^72^ In the current work, neither complex **1** nor **2** exhibited appreciable activity for the ROP of ε-caprolactone (see the Supporting Information).

**Figure 2:**
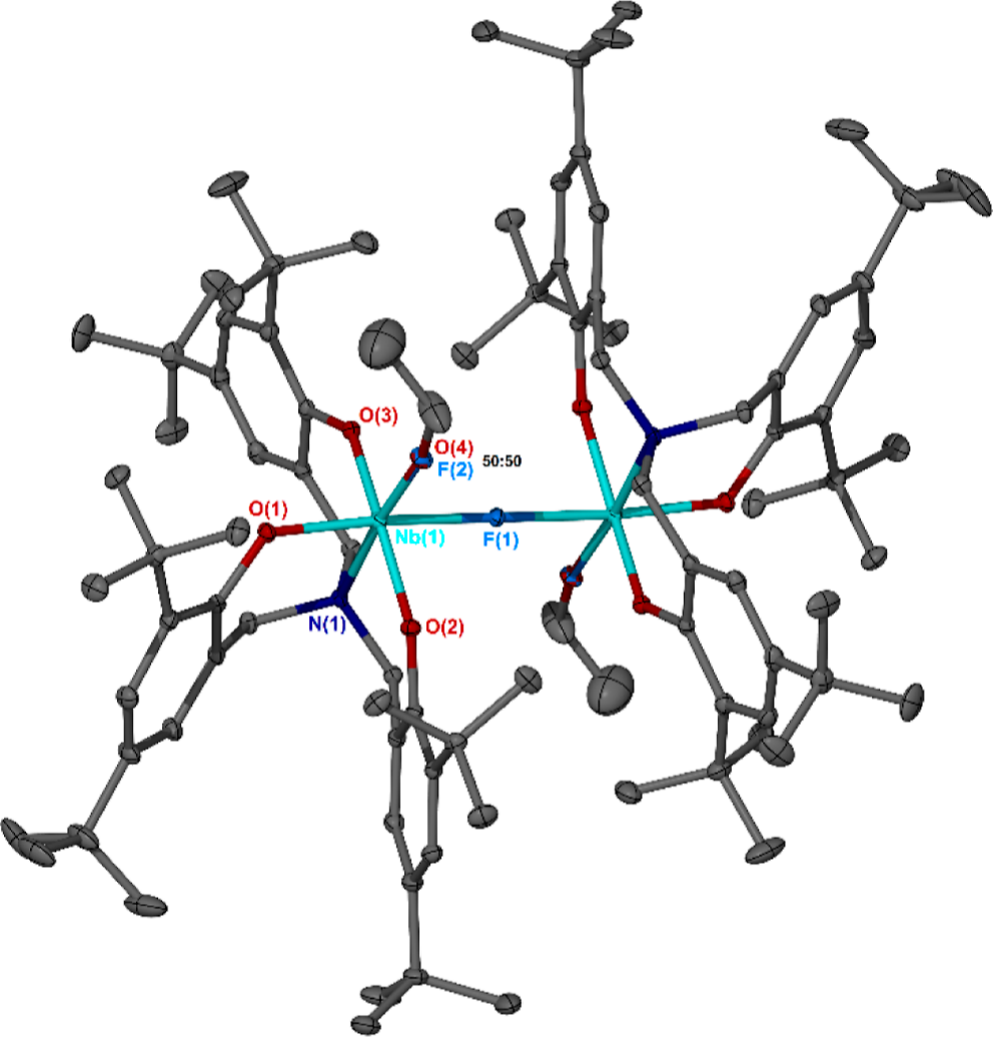
Crystal structure of the bimetallic, monocationic fragment of Nb(V) species **3**. Ellipsoids are shown at the 30% probability level. Hydrogen atoms, lattice solvent, and the SbF_6_^–^ anion have been omitted for clarity. The alkoxo and terminal fluoro ligands are distributed in a 50:50 ratio at the O(4)/F(2) position. Selected bond lengths (Å) and angles (deg): Nb(1)–F(1) 2.1058(3), Nb(1)–O(4)/F(2) 1.866(2), Nb(1)–N(1) 2.333(3), N(1)–Nb(1)–O(4)/F(2) 174.36(9), O(2)–Nb(1)–O(3) 161.51(9), O(1)–Nb(1)–O(4)/F(2) 100.12(9), O(2)–Nb(1)–F(1) 85.73(6), O(2)–Nb(1)–O(4)/F(2) 98.47(9), O(3)–Nb(1)–F(1) 85.48(6), O(3)–Nb(1)–O(4)/F(2) 97.87(9), F(1)–Nb(1)–O(4)/F(2) 90.69(6), and F(1)–Nb(1)–N(1) 83.70(6).

Substitution of the chloro ligand of **2** with the noncoordinating SbF_6_^–^ anion was attempted by treatment with 1.5 equiv of AgSbF_6_ at ambient temperature in toluene. Unexpectedly, this reaction afforded in high yield (78% isolated yield) the hexafluoroantimonate salt [{L^*t*Bu^Nb(OEt)}-μ_2_F-{L^*t*Bu^NbF}]^+^[SbF_6_]^−^, **3**, of a fluorobridged, bimetallic, monocationic monoalkoxide complex (Figure 2). **3** was isolated as a dark red, crystalline solid (Scheme 1). Unlike precursor complexes **1** and **2**, both amine tris(phenolate) ligands of **3** are *C*_1_-symmetric. The presence of two fluoro ligand environments (bridging and terminal, respectively) is attributed to fluoride abstraction from the anion of the excess AgSbF_6_, suggesting that the Nb centers are highly Lewis acidic.^74–80^ Moreover, when the synthesis or solvation of **3** in THF was attempted, the solvent was polymerized at ambient temperature (Supporting Information). Nonetheless, when the synthetic procedure employed for the synthesis of **3** was modified to use 2.33 equiv of AgSbF_6_, **3** remained the major product. Also detectable in that case, however, was a small quantity of a related species, **3a**, considered to be of no catalytic interest and therefore not subject to further synthetic efforts, in which both Nb centers bore terminal fluoro ligands, with no ethoxide moiety remaining (Supporting Information). The bis-ethoxide species, **3b**, bearing only a single fluoro ligand, in the μ_2_ position, was also detected via high-resolution mass spectrometry as a minor impurity of **3**. Considered incidental to this study, the synthesis of **3b** was similarly not pursued further.

**3** was found to be highly insoluble in toluene-*d*_8_, benzene-*d*_6_, and chloroform-*d*. ^1^H NMR spectra were also poorly resolved at 298 K, necessitating acquisition at low temperature, and indicating fluxionality of ^1^H environments on the NMR time scale. The methylene resonance corresponding to the alkoxide moiety was discernible at 298 K, but resolution of the methylene resonances of ligand (L^*t*Bu^)^3–^ necessitated the use of low-temperature techniques, and signals remained broad at 223 K.

**Figure 3:**
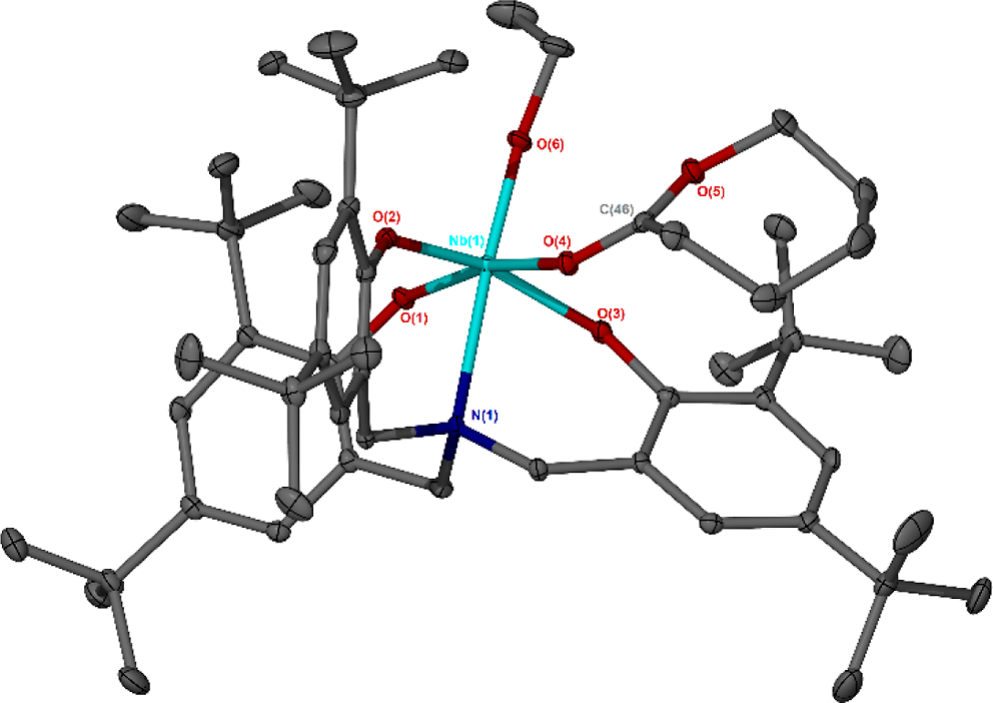
Crystal structure of the cationic fragment of Nb(V)-ε-caprolactone adduct **4**. Ellipsoids are shown at the 30% probability level. Hydrogen atoms, lattice solvent, and SbF_6_^–^ anion have been omitted for clarity. Selected bond lengths (Å) and angles (deg): Nb(1)–N(1) 2.340(3), Nb(1)–O(6) 1.833(2), Nb(1)–O(4) 2.165(2), O(4)–C(46) 1.244(4), O(5)–C(46) 1.293(5), N(1)–Nb(1)–O(6) 176.69(11), O(4)–Nb(1)–O(6) 95.64(10), O(1)–Nb(1)–O(4) 164.76(10), O(1)–Nb(1)–O(6) 99.27(11), O(2)–Nb(1)–O(3) 153.87(10), O(2)–Nb(1)–O(4) 77.76(10), O(2)–Nb(1)–O(6) 100.06(11), O(3)–Nb(1)–O(4) 82.75(10), O(3)–Nb(1)–O(6) 99.00(11), O(4)–Nb(1)–N(1) 82.40(9), and C(46)–O(4)–Nb(1) 135.5(3).

On reaction of **2** with 1 equiv of AgSbF_6_ and excess (4 equiv) ε-caprolactone (ε-CL) at ambient temperature in toluene, yellow crystals of the monometallic monocationic species [L^*t*Bu^Nb(OEt)(ε-CL)]^+^[SbF_6_]^−^, **4**, were obtained in good yield (68% isolated) (Figure 3 and Scheme 1). Preparation of **4** was facile on the gram scale, permitting thorough characterization and catalytic studies. **4** was also readily produced by addition of excess ε-CL to a suspension of **3** in CDCl_3_ at ambient temperature. On addition of ε-CL, **3** was rapidly solubilized, and ^1^H NMR analysis confirmed the presence of **4**, presumably formed by elimination of neutral byproduct [L^*t*Bu^NbF_2_] (Scheme 1). The concurrent presence of a coordinated cyclic ester molecule and alkoxide ligand at a metal center, as in **4**, is to the best of our knowledge unprecedented. The formation and stability of this species, despite the presence of excess ε-CL during both synthetic procedures by which it has been prepared, indicate that polymerization is not accessible at ambient temperature.

Unlike the bimetallic cationic fragment of **3**, and consistent with monometallic complexes **1** and **2**, the ancillary ligand of **4** adopts pseudo-*C*_3_ symmetry in the solid state. Furthermore, the carbonyl C=O bond length of the coordinated ε-CL moiety of **4** is 1.244(4) Å, suggesting greater activation of the monomer than that in the cationic methylaluminum species of Dagorne and co-workers (C=O = 1.217(6) Å).^41^ In the two existing examples of solid-state structures for ε-CL not coordinated to a metal center, wherein Yartseva et al. cocrystallized two equivalents of ε-CL with a triisocyanurate,and Yan et al. cocrystallized one equivalent with a trinitrotriazinane, the C=O distances were 1.230(8) and 1.203(9) Å, and 1.217(3) Å, respectively.^81,82^

The alkoxide and ε-CL moieties of **4** are spatially orientated such that initiation of ROP via intramolecular nucleophilic attack at the monomer carbonyl, facilitating insertion into the metal–alkoxide bond, appears feasible [O(6)–C(46) distance (not bonded) = 3.435 Å]. However, at ambient temperature, this does not occur, and **4** is readily stable under an inert atmosphere.

**Figure 4:**
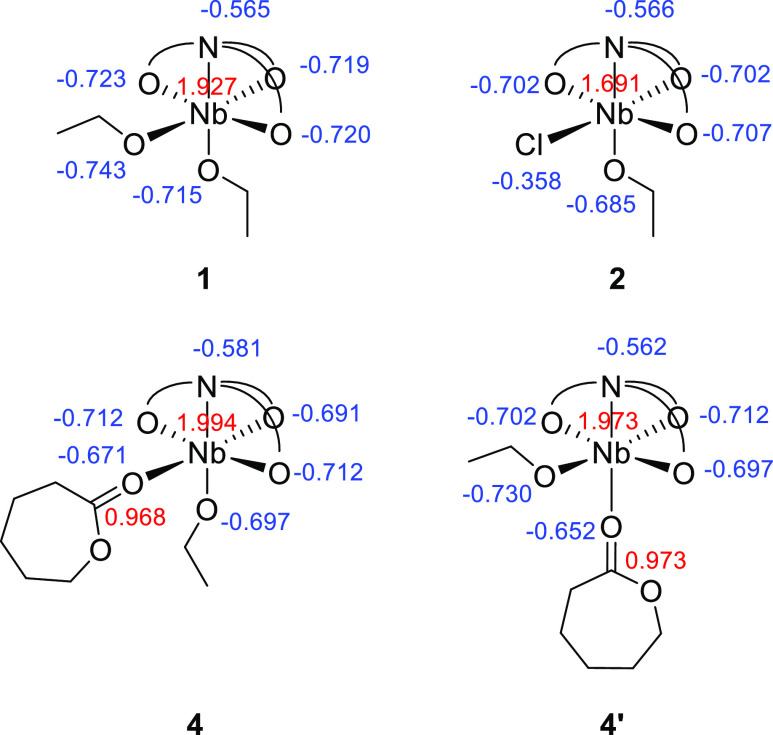
Nb(V) complexes **1**, **2**, **4**, and **4′** with NBO charges shown adjacent to the corresponding atoms (DFT protocol: rωB97XD/cpcm = toluene/6-31g(d)/6-311+g(d)/SDD).

We hypothesize that the alkoxide moiety of **4** is stabilized toward intramolecular nucleophilic attack by its position trans to the bridgehead nitrogen, consistent with the reports from the groups of Kol and Nomura regarding the relative inertness of the trans coordination site in group 5 amine tris(phenolate) systems.^71,73^ This is supported by our computational analysis of the natural charges on the Nb(V) center and donor atoms of complexes **1**, **2**, and **4**, and on the carbonyl group of the coordinated ε-CL molecule of **4** (Figure 4). The significantly different charges on the two alkoxide oxygen atoms, cis and trans to the nitrogen donor, of Nb(V) complex **1** are consistent with the difference in the reactivity of the two alkoxide moieties toward TMSCl observed for this species, as well as for complex **5** and the analogous Ta(V) complex reported by both Kol and Verkade.^52,71^ Furthermore, the reduced magnitude of the negative charge on the alkoxide oxygen of **4**, relative to the equivalent atom of **1**, is indicative of a concomitant reduction in nucleophilicity, resulting in a low affinity for attacking the carbonyl group of the coordinated monomer. This is compatible with the ROP of ε-CL initiated by **4** being inaccessible at ambient temperature.^83^ As expected for a cationic species, the positive charge on the Nb(V) center of **4** is also of greater magnitude than those of **1** and **2** (1.994 versus 1.927 and 1.691, respectively).

In addition to the confirmed existence of **4**, we surmised that isomerization of that species, proceeding via inversion of alkoxide and ε-CL positions about the Nb center, could give rise to a second, hypothetical adduct, **4′**. In **4′**, the negative charge on the alkoxide oxygen is calculated to be of greater magnitude than that in **4** (−0.730 versus −0.697), while the positive charge at the ε-CL carbonyl carbon is likewise increased.

Structurally, compared to that of **4**, the optimized geometry of **4′** also features a longer Nb–alkoxide bond (1.91363 Å vs 1.85702 Å), a shorter distance between the metal and the carbonyl group of the ε-CL moiety (2.13551 Å vs 2.18459 Å), as well as a longer carbonyl C=O bond length (1.24525 Å vs 1.23377 Å). These structural features highlight a potential need for **4** to isomerize to higher-energy species **4′** to become active in polymerization and explain its relative stability toward ROP at ambient temperature. It is further conceivable that any such isomerization process would also necessarily occur during each subsequent propagation event.

Although **4** was found to be much more readily soluble in several organic solvents than **3**, low-temperature ^1^H NMR spectroscopic techniques were still required to obtain well-resolved signals. The resulting NMR spectra of **4** in solution were consistent with the structure observed in the solid state. The major species detected via high-resolution mass spectrometry was [L^*t*Bu^Nb(OEt)]^+^, consistent with loss of ε-CL from the cationic component of **4**, although the intact cation, [L^*t*Bu^Nb(OEt)(ε-CL)]^+^, was also detected (Supporting Information). Elemental analysis was further consistent with **4** being isolated in high purity. Like **3**, solvation of **4** in THF resulted in polymerization of the solvent at ambient temperature, yielding poly(tetrahydrofuran). As expected for a catalytically active coordination product, unstable toward intramolecular nucleophilic attack, isomer **4′** was not observed spectroscopically, either during catalytic reactions or on incrementally heating a solution of **4** (see Figure S73 in the Supporting Information). The proposed transient species’ insufficient longevity for observation on the NMR time scale is consistent with the general absence of detectable preinsertion intermediates for catalytic coordination–insertion ROP protocols reported in the literature.

Using both **2** and **3** as starting materials, we were consistently unable to isolate or detect an l-lactide (l-LA) complex analogous to ε-CL complex **4**, and both **3** and **4** were inactive for the ROP of racemic lactide (*rac*-LA). We attribute this to a steric effect arising from the presence of lactide’s methyl substituents.^84^ The inertness of **4** toward lactide is further demonstrated by the facile nature of ε-CL homopolymerization from a solution containing equimolar quantities of ε-CL and l-lactide (Supporting Information).

**Scheme 2:**
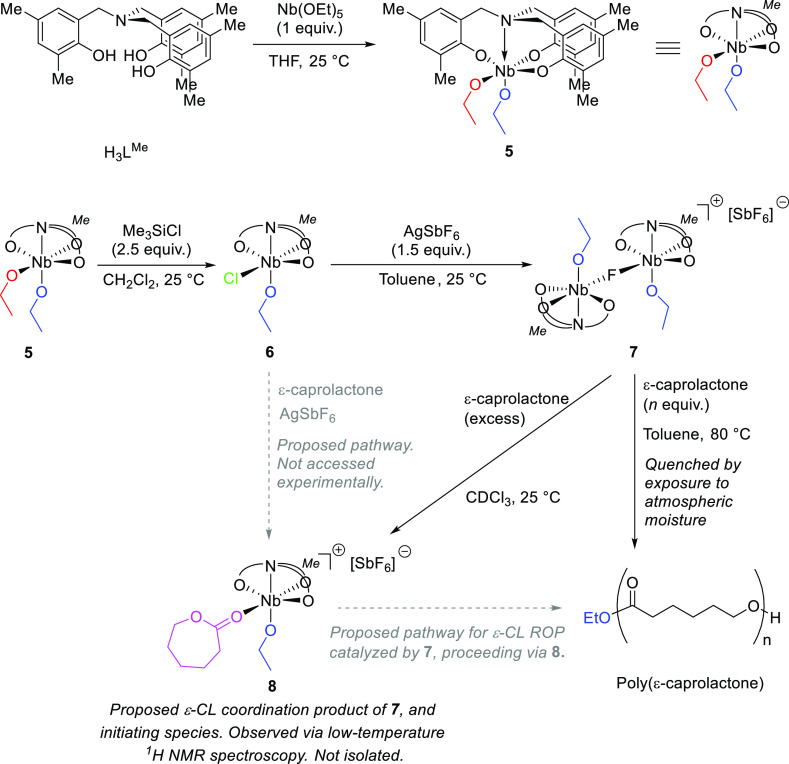
Synthesis and Reactivity of Methyl-Substituted Complexes **5–8**

Analogues **5** and **6** of neutral Nb alkoxides **1** and **2** were prepared using the methyl-substituted pro-ligand tris(2-hydroxy-3,5-dimethylbenzyl)amine, H_3_L^Me^ (Scheme 2). In the cases of both the bis(alkoxide) species, **1** and **5**, and the chlorinated derivatives, **2** and **6**, the proligands H_3_L^Me^ and H_3_L^*t*Bu^ yielded mutually isostructural, heteroleptic complexes [albeit with the (L^Me^)^3–^ and (L^*t*Bu^)^3–^ ligand scaffolds adopting distinct spatial arrangements in the solid state. See below]. This is in contrast to the structural distinction between the, respectively, heteroleptic monoalkoxide, and homoleptic, zwitterionic, *C*_3_-symmetric amine tris(phenolate) complexes, [L^*t*Bu^Zr(O^*i*^Pr)] and [Zr(HL^Me^)_2_], that we have previously reported for the preparation of by reaction of zirconium(IV) alkoxide precursor [Zr(O^*i*^Pr)_4_](HO^*i*^Pr) with H_3_L^*t*Bu^ and H_3_L^Me^.^46,85^ Unlike *tert*-butyl-substituted species **1** and **2**, however, and consistent with Kol and Verkade’s Ta systems,^52,71^ and with a Nb dichloro complex reported by Nomura and co-workers,^73^ the methyl-substituted ligand frameworks of Nb complexes **5** and **6** adopted *C*_1_-symmetry about the Nb–N axis in the solid state. At 298 K, the methylene NC*H*_2_ protons of **1** exhibited greater fluxionality on the NMR time scale than those of **5**, despite the greater steric profile of the (L^*t*Bu^)^3–^ ligand system, indicating that the difference in symmetry is retained in solution. Complexes **5** and **6**, much like **1** and **2**, exhibited negligible activity in the ROP of ε-CL (Supporting Information).

When **6** was treated with 1.5 equiv of AgSbF_6_ in toluene, small, red crystals of [{L^Me^Nb(OEt)}_2_-μ_2_F]^+^[SbF_6_]^−^, **7**, were isolated in low yield (35%). Solid-state analysis confirmed that **7** was structurally reminiscent of **3**. However, ethoxide groups remained at both Nb centers, with the cation bearing only one fluoro ligand located in the μ_2_ bridging position. This suggests that the metal-alkoxide bond of **7** is less labile than that of **3**. Furthermore, the ligand scaffolds binding each of the two Nb centers of the bimetallic cation of **7** exhibited distinct symmetries, with the aromatic rings adopting *C*_1_- and pseudo-*C*_3_-symmetric conformations, respectively. A methyl-substituted analogue, **8**, of ε-CL adduct **4** could not be isolated, although its formation was detected via ^1^H NMR spectroscopy on addition of ε-CL to a suspension of **7** in CDCl_3_ (see the Supporting Information). In similarity to **3**, treatment of **7** with l-LA produced no reaction. For further discussion of the synthesis and characterization of methyl-substituted Nb complexes, see the Supporting Information.

### Polymerization Studies

We anticipated that the cationic alkoxo-Nb ε-CL adduct **4** would be a stable structural analogue of the unobserved active species in the heteroselective coordination–insertion-type ROP of *rac*-LA proceeding in the presence of amine tris(phenolate)-supported Zr, Hf and Ge alkoxides.^46,47^ Indeed, the cationic complexes **3**, **4**, and **7** were all active initiators for the ROP of ε-CL in toluene when heated to 80 °C. In all cases, the crystalline Nb species were rapidly solubilized under the ROP conditions, this being a requisite for achieving good molecular weight control and presumably being attributable to the reaction with the monomer. Accordingly, the number-average molecular weight determined via gel permeation chromatography (GPC), *M*_n_^GPC^, of the polymer products produced in the presence of initiators **3**, **4**, and **7** was generally predictable.

**Scheme 3:**
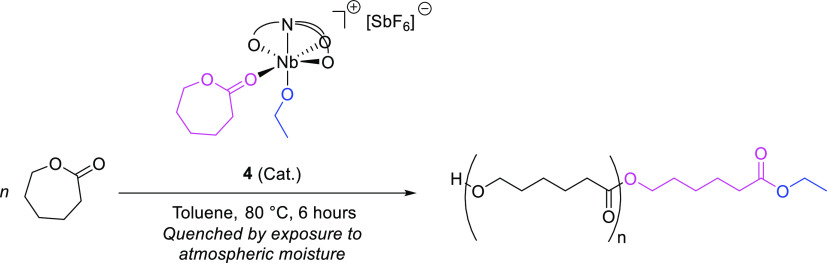
ROP of ε-Caprolactone in the Presence of **4**

When **4** was applied to the catalytic ROP of ε-CL (Scheme 3), good molecular weight control was observed. *M*_n_^GPC^ values were in general agreement with theoretical values, corresponding to approximately one initiation event occurring per molecule of **4** present (Table 1). This is compatible with insertion of the metal-coordinated ε-CL moiety into the adjacent metal–alkoxide bond, in a classical coordination–insertion mechanism. The molecular weight dispersity, *D̵*_M_, of the PCL produced in the presence of **4** was generally ≤1.50, which is characteristic of a controlled polymerization. On addition of co-initiator benzyl alcohol, BnOH, the polymer molecular weight was reduced, and *D̵*_M_ remained low, indicative of a nonrate-determining chain-transfer process characteristic of an immortal kinetic regime (Table 1, entries 5 versus 8, and 2 versus 7). Polymerization studies herein were carried out to obtain mechanistic insights and were therefore not optimized for catalytic efficiency. Accordingly, TON and TOF values have not been reported.

**Table 1:** Polymerization Data for the ROP of ε-Caprolactone in the Presence of **4**^a^

entry	[ε-CL]/[4]/[BnOH]	dconversion,%	e*M*_n_^GPC^	e*D̵*_M_	f*M*_n_^Theo^
b1	30:1:0	>99	4900	1.46	3585
c^,^^g^2	50:1:0	>99	9100	1.46	5867
b3	60:1:0	>99	8150	1.57	7007
b4	90:1:0	>99	12,000	1.54	10,435
c5	100:1:0	>99	16,400	1.42	11,574
c6	250:1:0	>99	20,250	1.50	28,695
c7	50:1:1	>99	4550	1.41	2988
c^,^^g^8	100:1:1	>99	4400	1.46	5841

a Conditions: 0.80 mol dm^–3^ ε-caprolactone solution in toluene, 80 °C for 6 h, in the presence of **4**, and exogenous BnOH where applicable.

b 500 mg ε-CL.

c 200 mg ε-CL.

d Conversion determined via ^1^H NMR spectroscopy, by integration of the monomer and polymer OCH_2_ methylene resonances.

e Determined via GPC analysis in THF using a refractive index detector calibrated against polystyrene standards, and with application of a conversion factor of 0.56.^12,30^

f *M*_n_^Theo^ calculated from conversion and catalyst concentration, .

g Reaction was carried out for 5 h.

Encouraged by the observed formation of **4** on addition of ε-CL to **3** at ambient temperature, we successfully investigated the utility of **3** as a precatalyst for **4**. As anticipated, the molecular weights of polymer samples produced in the presence of various loadings of **3** were commensurate with one initiation event occurring per molecule of the bimetallic, monoalkoxide precatalyst (Table 2). This is compatible with a mechanism proceeding via coordination of the monomer with concurrent cleavage of **3** and elimination of inactive fragment [L^*t*Bu^NbF_2_], followed by insertion of the monomer into the metal–alkoxide bond of the active cationic species. Molecular weight control was generally better with precatalyst **3** than it was with **4**, and *D̵*_M_ of the polymer samples was consistently ≤1.50.

**Table 2:** Polymerization Data for the ROP of ε-Caprolactone in the Presence of **3**^a^

entry	[ε-CL]/[3]/[BnOH]	dconversion, %	e*M*_n_^GPC^	e*D̵*_M_	f*M*_n_^Theo^
b1	30:1:0	>99	5000	1.40	3470
b2	60:1:0	>99	7350	1.50	6894
c3	106:1:0	>99	11,700	1.38	12,145
c4	250:1:0	>99	19,500	1.49	28,581
c5	100:1:1	>99	6450	1.29	5784
c6	250:1:2.5	>99	7750	1.19	8243

a Conditions: 0.80 mol dm^–3^ ε-caprolactone solution in toluene, 80 °C for 6 h, in the presence of **3**, and exogenous BnOH where applicable.

b 500 mg ε-CL.

c 200 mg ε-CL.

d Conversion determined via ^1^H NMR spectroscopy, by integration of the monomer and polymer OCH_2_ methylene resonances.

e Determined via GPC analysis in THF using a refractive index detector calibrated against polystyrene standards and with application of a conversion factor of 0.56.^12,30^

f *M*_n_^Theo^ calculated from conversion and catalyst concentration, .

When exogenous BnOH was present, the polymerization of ε-caprolactone catalyzed by **3** yielded polymer of low *D̵*_M_ and of lower-molecular weight than when BnOH was not present (Table 2 entries 3 and 4 versus entries 5 and 6). Furthermore, the molecular weights of PCL obtained were in good agreement with theoretical values, where each molecule of benzyl alcohol was assumed to initiate growth of one polymer chain, in addition to those initiated by the precatalyst alkoxide moiety.

Although **7** could not be isolated in high purity (see the Supporting Information), a cursory study of its use as a precatalyst in the ROP of ε-CL was nevertheless undertaken. Molecular weight control was good, especially at high catalyst concentrations (Table 3, entries 1–3). In commonality with analogous precatalyst **3**, the molecular weights of polymer samples produced in the presence of **7** appeared to correspond to one initiation event occurring per molecule of the precatalyst. Despite reduced steric bulk, the dispersity of PCL was observed to be generally narrower when produced using **7** than when **3** or **4** were employed, under otherwise identical conditions. This is likely attributable to a faster initiation event, relative to propagation, in the presence of the less sterically encumbered system.

**Table 3:** Polymerization Data for the ROP of ε-Caprolactone in the Presence of **7**^a^

entry	[ε-CL]/[7]	dconversion, %	e*M*_n_^GPC^	e*D̵*_M_	f*M*_n_^Theo^
b1	30:1	>99	3150	1.23	3471
c2	50:1	>99	5200	1.30	5753
b3	60:1	>99	6750	1.59	6894
c4	100:1	>99	8500	1.32	11,460
c5	250:1	>99	15,700	1.33	28,581

a Conditions: 0.80 mol dm^–3^ ε-caprolactone solution in toluene, 80 °C for 6 h, in the presence of **7**.

b 500 mg ε-CL.

c 200 mg ε-CL.

d Conversion determined via ^1^H NMR spectroscopy, by integration of the monomer and polymer OCH_2_ methylene resonances.

e Determined via GPC analysis in THF using a refractive index detector calibrated against polystyrene standards and with application of a conversion factor of 0.56.^12,30^

f *M*_n_^Theo^ calculated from conversion and catalyst concentration, .

Polymer end group analysis was carried out to investigate the proposed coordination–insertion mechanism at the respective alkoxide moieties of **3**, **4**, and **7**. Accordingly, the polymer products of reactions catalyzed by various loadings of each initiator were purified by precipitation from, and copious washing with, methanol to remove catalyst and monomer residues, and other small molecule contaminants, then dried under dynamic vacuum and the end group analyzed via ^1^H NMR spectroscopy. In the cases of initiators **3** and **4**, the ratio of methylene signals corresponding to the polymer backbone and ethoxy (EtO–/H−) end group (appearing at δ = 4.05 and δ = 4.12 ppm, respectively, in CDCl_3_) was consistently in excellent agreement with the precatalyst loading (see Table S1 and Figure S74 in the Supporting Information). Such quantitative retention of ethoxy residues on precipitation of the PCL from methanol at several catalyst loadings, in conjunction with good molecular weight control established via GPC analysis, is characteristic of the ethoxide groups of **3** and **4**, respectively, being the principal initiating groups in a living coordination–insertion ROP pathway. This is significant in confirming that the ε-CL adduct, **4**, is indeed the first intermediate of a coordination–insertion mechanism. Consistent with this, sustained heating of a solution of **4** to 75 °C in chloroform-*d* caused significant changes in the ^1^H NMR spectrum, including a downfield migration of the ethoxymethylene resonance and the appearance of signals corresponding to a metal-coordinated PCL polymer chain end (see Figure S73 in the Supporting Information). This is conclusively attributed to insertion of the coordinated ε-CL molecule into the metal–alkoxide bond. Moreover, as expected, we conclude that on cleavage of the precatalyst **3**, the ionic ethoxide-bearing fragment, **4**, is responsible for all polymerization activity.

In the case of precatalyst **7**, the concentration of ethoxy polymer end groups corresponded to two initiation events occurring per molecule of the precatalyst, suggesting that both of the alkoxide groups present on the bimetallic cationic fragment of species **7** initiated polymer chain growth (whereas the bimetallic cation of **3** bears only one alkoxide moiety). This is compatible with a posited mechanistic scenario, in which the active catalyst and inactive bimetallic species exist in equilibrium during ROP (Supporting Information).

## Computational Analysis

Motivated by the indication provided by NBO calculations, vide supra, that ROP of ε-CL in the presence of **4** may require isomerization to **4′**, a preliminary density functional theory (DFT) study of the initiation event was undertaken. Intermediates and transition states were modeled for two distinct coordination–insertion pathways, proceeding in both cases via intramolecular nucleophilic attack and subsequent ring-opening events starting from coordination product **4**, respectively, with and without first undergoing isomerization to afford **4′** (or a closely related structure, **I′**, arising from rotation of the ε-CL moiety prior to isomerization). The calculated Gibbs free-energy values for **4′** (+14.6, +13.6, and +14.5 kcal mol^–1^ for the ωB97XD, B3LYP-D3BJ, and M06-D3 functionals, respectively, relative to **4**) and **I′** (+13.0, +12.8, and +12.6 kcal mol^–1^ for the ωB97XD, B3LYP-D3BJ, and M06-D3 functionals, respectively, relative to **4**) were consistent with initiation in the ROP of ε-CL proceeding via isomerization of **4**, and with the isomerization product, **4′** or **I′**, being insufficiently stable to permit isolation or detection under experimental conditions. While the results of the DFT calculations were qualitatively supportive of the proposed “coordination–inversion–insertion” mechanism, the transition states corresponding to nucleophilic attack and ring-opening for this pathway being lower in energy than those optimized without a prior inversion event, the absolute Δ*G*^⧧^ values obtained were markedly higher than would be anticipated, or predicted by transition-state theory, for a ROP process proceeding under the conditions described herein. Detailed results and a discussion of the DFT study can be found in the Supporting Information.

It is plausible that an inversion event similar to that by which the activation of **4** (and, presumably, the propagating species) toward intramolecular nucleophilic attack is suggested to occur may underlie the dynamic enantiomorphic site control mechanism through which our previously reported Zr, Hf, and Ge amine tris(phenolate) systems have been proposed to effect the heteroselective ROP of *rac*-LA.^46,47^

## Conclusions

In conclusion, we have prepared seven new Nb(V) complexes, including three cationic species, as the hexafluoroantimonate salts **3**, **4**, and **7**. **3**, **4**, and **7** are all active initiators for the living ROP of ε-caprolactone in toluene at 80 °C, expanding the sparse catalogue of group 5 catalysts for the polymerization of cyclic esters. We have shown that the bimetallic cation of **3** breaks down on addition of the monomer to yield the active catalyst **4**. **4** is also readily prepared in bulk and represents, to the best of our knowledge, the first characterized metal complex bearing both a coordinated cyclic ester and an alkoxide initiating group simultaneously. Furthermore, the polymerization of ε-CL in the presence of **3**, **4**, and **7** occurs in each case by insertion of the monomer into the respective initiator’s metal–alkoxide bond, describing a classical coordination–insertion mechanism. The mechanistic relevance of **4** as the first intermediate in a coordination–insertion pathway is supported by experimental and computational findings, providing new insights into a widely reported mechanism. In particular, NBO calculations demonstrate that polymerization is likely to require the conformational rearrangement of the stable ε-CL adduct, **4**. We tentatively suggest that a similar isomerization process may form the basis of the dynamic enantiomorphic site to which stereocontrol has been attributed in the application of structurally related, neutral amine tris(phenolate)-supported Zr, Hf, and Ge alkoxide initiators to the heteroselective ROP of *rac*-lactide.^46,47^

## Supporting Information Available

Complete synthetic procedures and characterization data for species **1–7**, polymerization procedures, polymer characterization data, and kinetic data (PDF)

## Abbreviations

ROP: ring-opening polymerization
ε-CL: ε-caprolactone
PCL: poly(ε-caprolactone)
LA: lactide
*rac*-LA: racemic lactide
PLA: poly(lactic acid)
THF: tetrahydrofuran
TMSCl: chlorotrimethylsilane
H_3_L^*t*Bu^: tris(2-hydroxy-3,5-di-*tert*-butylbenzyl)amine
H_3_L^Me^: tris(2-hydroxy-3,5-dimethylbenzyl)amine
*D̵*_M_: dispersity (of polymer molecular weight distribution)
*M*_n_: number-average molecular weight
*M*_n_^GPC^: number-average molecular weight determined via GPC
GPC: gel permeation chromatography
*M*_n_^Theo^: theoretical number-average molecular weight
NBO: natural bond orbital
DFT: density functional theory
