# Supplementary material for: Coordination of ε-Caprolactone to a Cationic Niobium(V) Alkoxide Complex: Fundamental Insight into Ring-Opening Polymerization via Coordination–Insertion
Source: Inorg Chem. 2023 Sep 11;62(38):15688–99. doi: 10.1021/acs.inorgchem.3c02491 (PMC10523432; doi:10.1021/acs.inorgchem.3c02491)
Supplement: Supplementary file 1 — ic3c02491_si_001.pdf [file ic3c02491_si_001.pdf]

Supporting Information

Coordination of ε-Caprolactone to a Cationic Niobium(V) Alkoxide Complex: Fundamental Insight into Ring-Opening Polymerization via Coordination-Insertion

Antoine Buchard,[a,b] Matthew G. Davidson,[a,b] Gerrit Gobius du Sart,[c] Matthew D. Jones,[a,b]*

Gabriele Kociok-Köhn,[d] Strachan N. McCormick[a,b] and Paul McKeown[b]*

[a] Institute for Sustainability, University of Bath, Bath BA2 7AY, United Kingdom

[b] Department of Chemistry, University of Bath, Bath BA2 7AY, United Kingdom

[c] TotalEnergies Corbion, Stadhuisplein 70, 4203 NS Gorinchem, The Netherlands

[d] Material and Chemical Characterization and Analysis Facility (MC^2), University of Bath, Bath BA2 7AY, United Kingdom

E-mail: S.N.McCormick@bath.ac.uk, M.G.Davidson@bath.ac.uk

Contents

Materials and Methods 4
Synthetic procedures and analytical data 7
Synthesis of amine tris(phenolate) pro-ligands 7
Synthesis of pro-ligand tris(2-hydroxy-3,5-di-*tert*-butylbenzyl)amine, H_3L^{tBu} 7
Synthesis of pro-ligand tris(2-hydroxy-3,5-dimethylbenzyl)amine, H_3L^{Me} 7
Synthesis of Niobium amine tris(phenolate) complexes 8
Synthesis of Niobium species $[L^{tBu}Nb(OEt)_2]$, 1 8
Synthesis of Niobium species $[L^{tBu}Nb(OEt)Cl]$, 2 13
Synthesis of Niobium species $[\{L^{tBu}Nb(OEt)\}\text{-}\mu_2F\text{-}\{L^{tBu}NbF\}]^+[SbF_6]^-$, 3 17
Synthesis of Niobium species $[L^{tBu}Nb(OEt)(\varepsilon\text{-CL})]^+[SbF_6]^-$, 4 28
Synthesis of Niobium species $[L^{Me}Nb(OEt)_2]$, 5 35
Synthesis of Niobium species $[L^{Me}Nb(OEt)Cl]$, 6 40
Synthesis of Niobium species $[\{L^{Me}Nb(OEt)\}_2\text{-}\mu_2F]^+[SbF_6]^-$, 7 45
Stoichiometric reactions 48
Addition of ε-caprolactone to Nb complex 3 48
Addition of *L*-lactide to Nb complex 3 50
Addition of ε-caprolactone to Nb complex 7 52
Heating Nb complex 4 54
Polymerisation studies 55
General polymerisation procedure 55
End-group analysis 56
Kinetic studies of ε-caprolactone polymerisation 60
General procedure for kinetic studies 60
Polymer characterisation data for kinetic studies 62
Control reactions 62
Attempted polymerisation of *rac*-lactide 62
Attempted polymerisation of ε-CL in the presence of neutral Nb(V) complexes 62
Polymerisation of ε-caprolactone in the presence of *L*-lactide 63
General procedure 63
Kinetic data and analysis 63
Polymerisation of THF 66
Example gel permeation chromatograms 68
Computational details 70
NBO calculations 70
Free enthalpy profile for the initiation step of ε-caprolactone ROP from 4 70

Additional discussion of DFT results 73
Crystallographic Parameters 75
References 76

Materials and Methods

Caution! *All solvents and reagents should be handled in accordance with standard laboratory procedures, and according to their associated GHS Hazard Statements and Precautionary Statements. Reagents presenting GHS category 1 hazards (including sub categories 1A and 1B) are specifically identified below.*

Caution! *Chlorotrimethylsilane presents a GHS category 1 hazard of serious eye damage, and a GHS sub-category 1A hazard of skin corrosion.*

Caution! *Niobium(V) ethoxide presents a GHS category 1B hazard of skin corrosion.*

Caution! *2,4-Dimethylphenol presents a GHS sub-category 1B hazard of skin corrosion.*

Caution! *2,4-di-tert-butylphenol presents GHS category 1 hazards of serious eye damage, short-term aquatic hazard, and long-term aquatic hazard.*

Caution! *Paraformaldehyde presents GHS category 1 hazards of serious eye damage and skin sensitization, and a GHS sub-category 1B hazard of carcinogenicity.*

Caution! *Hexamethylenetetramine presents a GHS sub-category 1B hazard of skin sensitization.*

Caution! *Use of a Schlenk line (vacuum manifold) requires care due to the presence of a liquid nitrogen-cooled solvent trap, and the hazards associated both with the use of a cryogenic fluid and with the potential for condensation of liquid oxygen in the presence of organic solvents.*

Caution! *Transfer, handling and, specifically, syringe filtration of hazardous materials in a glove box with use of hypodermic needles should be undertaken with care to mitigate the risk of personal injury.*

Caution! *All Niobium(V) complexes prepared in the current work should be assumed to be highly toxic by all exposure routes and handled according to best laboratory practice. In particular, hexafluoroantimonate salts **3**, **4** and **7** have been observed to be highly Lewis acidic and, accordingly, may readily react with biological tissues on exposure.*

Manipulations, unless otherwise specified, were carried out under an atmosphere of dry argon using standard Schlenk line and glove box techniques. An MBraun glove box was used, equipped with internal taps supplying dry toluene, dichloromethane and hexane directly from an adjacent MBraun solvent purification system (SPS). The glove box atmosphere was maintained with O_2 and H_2O concentrations <10.0 ppm and <0.1 ppm, respectively.

Tetrahydrofuran, toluene, dichloromethane and hexane were drawn from an MBraun solvent purification system and stored over molecular sieves unless otherwise specified.

Niobium(V) ethoxide, $Nb(OEt)_5$, and silver hexafluoroantimonate, $AgSbF_6$, were purchased from Strem Chemicals (UK) and used under a dry argon atmosphere without further purification. Chlorotrimethylsilane, TMSCl, was purchased from Sigma Aldrich and used under a dry argon atmosphere without further purification.

Anhydrous benzyl alcohol was purchased from Sigma Aldrich and degassed under dynamic vacuum for 20 h before storage and use under a dry argon atmosphere. 2,4-dimethylphenol, 2,4-di-*tert*-butylphenol, hexamethylenetetramine (HMTA), and paraformaldehyde were all purchased from Sigma Aldrich and used without further purification.

rac-lactide was purchased from Sigma Aldrich and recrystallized 3 times from dry toluene. This was carried out under ambient air, and each recrystallization was followed by washing sparingly with dry

toluene over a sintered glass frit. The recrystallized *rac*-lactide was then dried under dynamic vacuum for 16 h and stored under a dry argon atmosphere. *L*-lactide was supplied by Total Corbion and purified in the same manner described for *rac*-lactide. ε-caprolactone was purchased from Sigma Aldrich and dried over calcium hydride, then filtered and vacuum distilled before storage and use under a dry argon atmosphere.

All NMR spectra were acquired using a 400 MHz (^{1}H), 101 MHz (^{13}C), or 500 MHz (^{1}H), 126 MHz (^{13}C), Bruker Avance spectrometer, unless otherwise stated. $CDCl_3$ was purchased from Sigma Aldrich and used as received for polymer analysis. For analysis of metal complexes, $CDCl_3$ was dried over calcium hydride, distilled under vacuum, and stored over 4 Å molecular sieves under a dry argon atmosphere. Toluene-d_8 was purchased from Sigma Aldrich and stored over 4 Å molecular sieves under a dry argon atmosphere. Processing was carried out using Mestrelab Research MestReNova version 11.0.2-18153.

All crystallographic data was collected on a SuperNova, EOS detector diffractometer using radiation Cu-Kα (λ= 1.54184 Å) or Mo-Kα (λ= 0.71073 Å) all recorded at 150(2) K. All structures were solved by direct methods and refined on all F2 data using the SHELXL-2014 suite of programs. All hydrogen atoms were included in idealized positions and refined using the riding model.

Special refinement details:

1: One t-Bu and one OEt-group in the Nb complex show disorder in the ratio 80:20 involving atoms (C32-C34/C32A-C34A) and atoms (C1-C2/C1A-C2A). C1A and C2A have been refined with ADP restraints. Half a solvent molecule of toluene is located near a 4-fold axis with its methyl group sitting right on it which leads to heavy disorder.

Bond length constraints and PART-1 instructions have been applied for the phenyl part of the molecule.

3: The main molecule forms a dimer linked via a F-atom. Two ligands attached to the Nb atoms share the same site (F/OEt=1:1). SbF_6 and one toluene are disordered over a two-fold axis and have been refined with 50% occupation. Another toluene molecule is disordered over a two sites in the ratio 1:1. However, it was refined with 40% occupation each for both sites.All atoms involved in the disorders except for Sb have been refined with ADP restraints.

3a: The methyl groups of one tBu (C35) were disordered over two positions in a 70:30 ratio; three molecules of disordered toluene and one benzene also present in unit cell.

5: One CH_2CH_3 of an ethoxide (O(1)) is disordered over two positions C(1)/C(2) and C(1A)/C(2A) in a ratio of 40:60.

6: The asymmetric unit contains a solvent mixture of THF and toluene in the ratio 1:1 which was refined with 40% occupation each. Both molecules share the same site and were refined with ADP restraints. The toluene was refined with geometric constraints.

Polymer molecular weight data was acquired using an Agilent 1260 Gel Permeation Chromatography (GPC) system with refractive index detector, calibrated against 12 polystyrene standards. A PLgel 5 µm MIXED-D 300 x 7.5 mm column was used, with a PLgel 5 µm MIXED Guard 50 x 7.5 mm guard column. The mobile phase was THF, at a flow rate of 1 ml min^{-1}. Columns and detectors were maintained at 35 °C. Data was processed using Agilent's GPC/SEC Software, Revision A.02.01.

High-resolution mass spectra were acquired using a MaXis HD quadrupole electrospray time-of-flight (ESI-QTOF) mass spectrometer (Bruker Daltonik GmbH, Bremen, Germany) for infusions. Analyses were performed in ESI positive mode. The capillary voltage was set to 4500 V, nebulizing gas at 0.4

bar, drying gas at 4 L/min at 180°C. The TOF scan range was from 300 – 2500 mass-to-charge ratio (m/z). Infusions were performed at 3µL/min. The MS instrument was calibrated using sodium formate calibrant solution. The calibrant solution consisted of 3 parts of 1 M NaOH to 97 parts of 50:50 water:isopropanol with 2% formic acid. The observed mass and isotope pattern matched the corresponding theoretical values as calculated from the expected elemental formula within 2 ppm mass accuracy. Mass features were detected as [M]+ ions. Data processing was performed using the Compass Data Analysis software version 4.3 (Bruker Daltonik GmbH, Bremen, Germany). All other mass spectrometry was carried out with a Bruker Daltonik microToFelectrospray time-of-flight (ESI-ToF) mass spectrometer. Samples were dissolved in acetonitrile at a concentration of 10 µg mL^{-1}, and positive ionisation mode was used.

Synthetic procedures and analytical data

Synthesis of amine tris(phenolate) pro-ligands

Synthesis of pro-ligand tris(2-hydroxy-3,5-di-*tert*-butylbenzyl)amine, H_3L^{tBu}

Pro-ligand H_3L^{tBu} was synthesised according to the following adapted literature procedure.[1] To hexamethylenetetramine (97 mmol, 13.5 g, 1 equivalent), was added 2,4-di-*tert*-butylphenol (1163 mmol, 40 g, 12 equivalents) paraformaldehyde (775 mmol, 23.3 g, 8 equivalents), and deionised water (3.89 mol, 71.1 ml, 40 equivalents). The mixture was then refluxed with vigorous stirring for 120 hours in an oil bath at 150 °C, with further 10 ml aliquots of 2,4-dimethylphenol added daily. The reaction mixture was cooled and the resulting yellow-white solid washed over a glass frit with MeOH (5 x 800 ml), to yield a white powder. The powder was then dried under dynamic vacuum for 24 hours. ^{1}H and $^{13}C\{^{1}H\}$ NMR data in $CDCl_3$ was in agreement with the literature. Yield: 171 g, 65 %

Synthesis of pro-ligand tris(2-hydroxy-3,5-dimethylbenzyl)amine, H_3L^{Me}

Pro-ligand H_3L^{Me} was synthesised according to the following adapted literature procedure.[1] To hexamethylenetetramine (68.18 mmol, 9.5 g, 1 equivalent) was added 2,4-dimethylphenol (818.00 mmol, 100 g, 12 equivalents), paraformaldehyde (545 mmol, 16.4 g, 8 equivalents), and deionised water (2.72 mol, 50 ml, 40 equivalents). The mixture was then refluxed with vigorous stirring for 120 hours in an oil bath at 150 °C, with two further 10 ml aliquots of 2,4-dimethylphenol added after 72 hours and 90 hours respectively. The reaction mixture was cooled and the resulting orange solid washed over a glass frit with MeOH (5 x 400 ml), to yield a white powder. The powder was then dried under dynamic vacuum for 24 hours. ^{1}H and $^{13}C\{^{1}H\}$ NMR data in $CDCl_3$ was in agreement with the literature. Yield: 63 g, 55 %

Synthesis of Niobium amine tris(phenolate) complexes

Synthesis of Niobium species [$L^{tBu}Nb(OEt)_2$], 1

To a solution of H_3L^{tBu} (37.3 mmol, 25.0 g, 1 equivalent) in THF was added by cannula transfer a solution of $Nb(OEt)_5$ (37.3 mmol, 11.9 g, 1 equivalent) in THF at 25 °C. A yellow colour was immediately observed. After stirring for two hours at ambient temperature, the solvent was removed under dynamic vacuum to yield an oily yellow solid. Washing sparingly with hexane (2 x 50 ml) yielded a pale yellow powder which, after filtration, was dried under dynamic vacuum. Yield: 25.0 g, 79 %. Crystals suitable for diffraction were obtained by recrystallizing from a mixture of hexane and toluene.

^{1}H NMR (400 MHz, Toluene-*d_8*, 298 K, δ_H, ppm); 7.49 (1H, *d*, *J* = 2.5 Hz, *Ar*), 7.43 (2H, *d*, *J* = 2.5 Hz, *Ar*), 6.97 (2H, *d*, *J* = 2.5 Hz, *Ar*, *overlapping with toluene-d_8*), 6.84 (1H, *d*, *J* = 2.5 Hz, *Ar*), 4.81 (2H, *q*, *J* = 7.0 Hz, OCH_2CH_3), 3.55 (6H, *s*, broad, NCH_2), 3.49 (2H, *q*, *J* = 7.0 Hz, OCH_2CH_3), 1.73 (9H, s, $C(CH_3)_3$), 1.57 (18H, *s*, $C(CH_3)_3$), 1.47 (3H, *t*, *J* = 7.0 Hz, OCH_2CH_3), 1.35 (9H, *s*, $C(CH_3)_3$), 1.34 (18H, *s*, $C(CH_3)_3$), 0.53 (3H, *t*, *J* = 7.0 Hz, OCH_2CH_3). **^{1}H NMR** (400 MHz, $CDCl_3$, 298 K, δ_H, ppm); 7.24 (1H, *d*, *J* = 2.2 Hz, Ar), 7.21 (2H, *d*, *J* = 2.2 Hz, Ar), 6.96 (2H, *d*, *J* = 2.2 Hz, Ar), 6.90 (1H, *d*, *J* = 2.2 Hz, Ar), 4.78 (2H, *q*, *J* = 7.0 Hz, OCH_2CH_3), 3.69 (6H, *s*, broad, NCH_2, *overlapping with residual THF*), 3.41 (2H, *q*, *J* = 7.0 Hz, OCH_2CH_3), 1.54 (9H, *s*, $C(CH_3)_3$), 1.45 (3H, *t*, *J* = 7.0 Hz, OCH_2CH_3), 1.39 (18H, *s*, $C(CH_3)_3$), 1.29 (18H, *s*, $C(CH_3)_3$), 1.24 (9H, *s*, $C(CH_3)_3$), 0.53 (3H, *t*, *J* = 7.0 Hz, OCH_2CH_3). **^{13}C{^{1}H} NMR** (101 MHz, Toluene-*d_8*, 298 K, δ_c, ppm); 157.3 (*Ar*O), 156.8 (*Ar*O), 141.7 (*Ar*), 141.5 (*Ar*), 136.7 (*Ar*), 125.8 (*Ar*), 124.8 (*Ar*, *partially obscured by toluene-d_8*), 124.5 (*Ar*H), 124.0 (*Ar*H), 123.7 (*Ar*H), 123.6 (*Ar*H), 72.1 (OCH_2), 67.6 (OCH_2), 62.0 (NCH_2), 61.9 (NCH_2), 35.4 ($C(CH_3)_3$), 35.4 ($C(CH_3)_3$), 34.5 ($C(CH_3)_3$), 34.4 ($C(CH_3)_3$), 32.0 ($C(CH_3)_3$), 32.0 ($C(CH_3)_3$), 30.3 ($C(CH_3)_3$), 30.3 ($C(CH_3)_3$), 19.0 (OCH_2CH_3), 18.6 (OCH_2CH_3). **^{13}C{^{1}H} NMR** (101 MHz, $CDCl_3$, 298 K, δ_c, ppm); 156.6 (*Ar*O), 141.5 (*Ar*), 141.3 (*Ar*), 137.3 (*Ar*), 136.3 (*Ar*), 125.7 (*Ar*), 124.6 (*Ar*), 124.0 (*Ar*H), 123.8 (*Ar*H), 123.5 (*Ar*H), 123.3 (*Ar*H), 71.9 (OCH_2), 67.5 (OCH_2), 62.1 (NCH_2), 61.9 (NCH_2), 35.2 ($C(CH_3)_3$), 35.1 ($C(CH_3)_3$), 34.4 ($C(CH_3)_3$), 34.4 ($C(CH_3)_3$), 31.9 ($C(CH_3)_3$), 31.8 ($C(CH_3)_3$), 30.1 ($C(CH_3)_3$), 30.0 ($C(CH_3)_3$), 18.8 (OCH_2CH_3), 18.4 (OCH_2CH_3). **Elemental (CHN) Analysis** (Calculated, for $C_{49}H_{76}NNbO_5$); C: 69.07 %, H: 8.99 %, N: 1.64 %, (Experimental); C: 68.94 %, H: 9.17 %, N: 1.73 %.

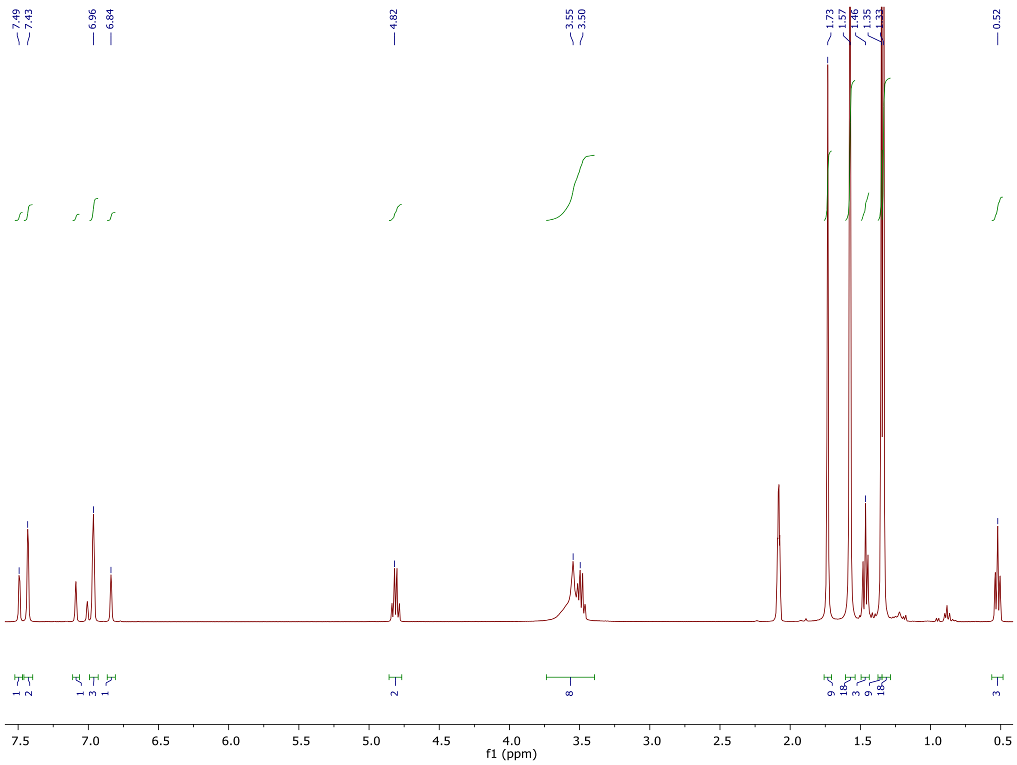

Figure S1. ^{1}H NMR spectrum of **1** in toluene-*d₈* at 298 K

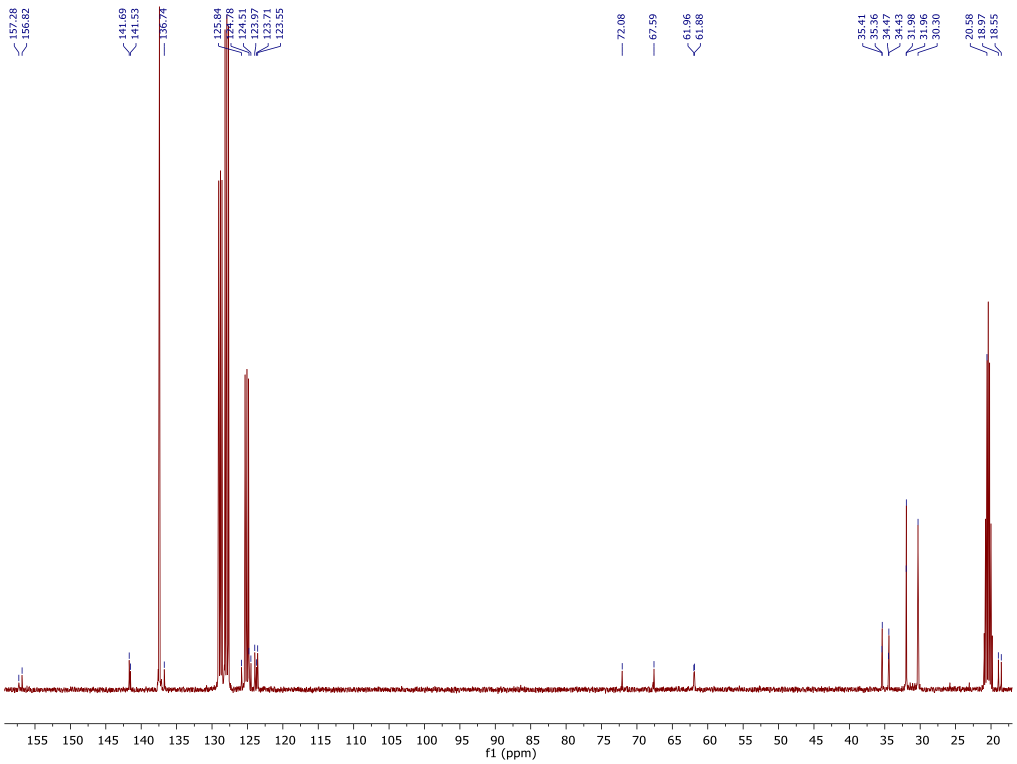

Figure S2. ^{13}C{^{1}H} NMR spectrum of **1** in toluene-*d₈* at 298 K

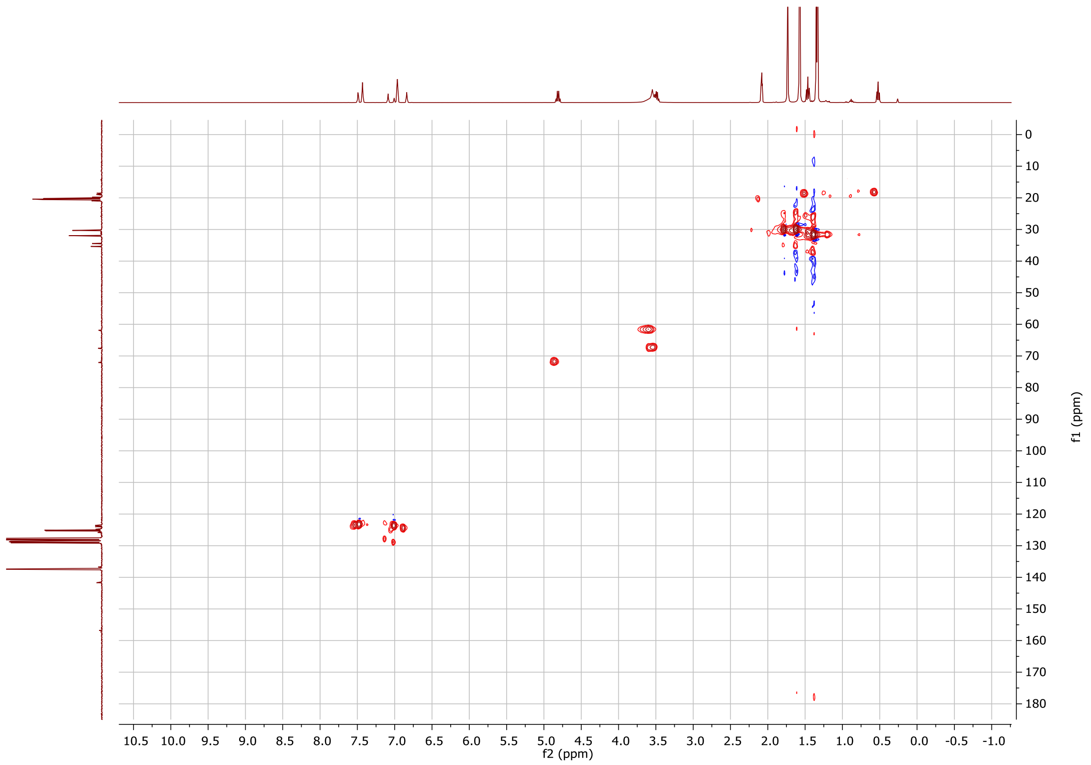

Figure S3. HSQC NMR spectrum of **1** in toluene-d_8 at 298 K

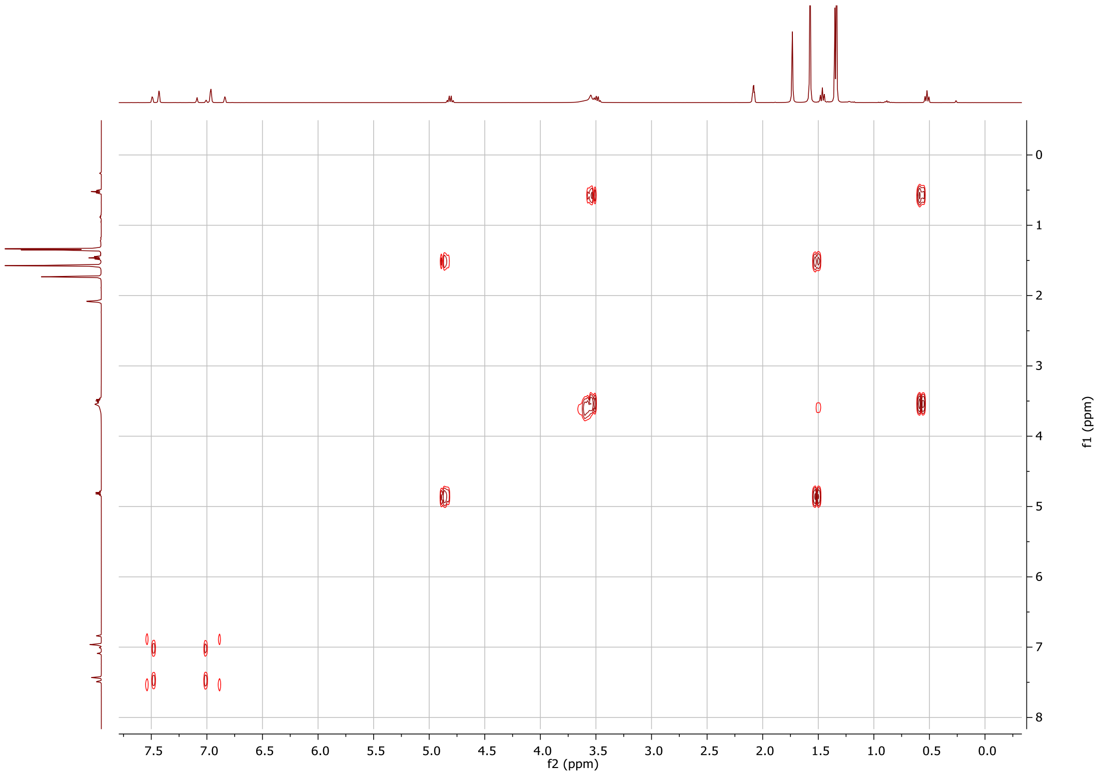

Figure S4. COSY NMR spectrum of **1** in toluene-d_8 at 298 K, allowing correct assignment of alkoxide CH_3 and CH_2 resonances

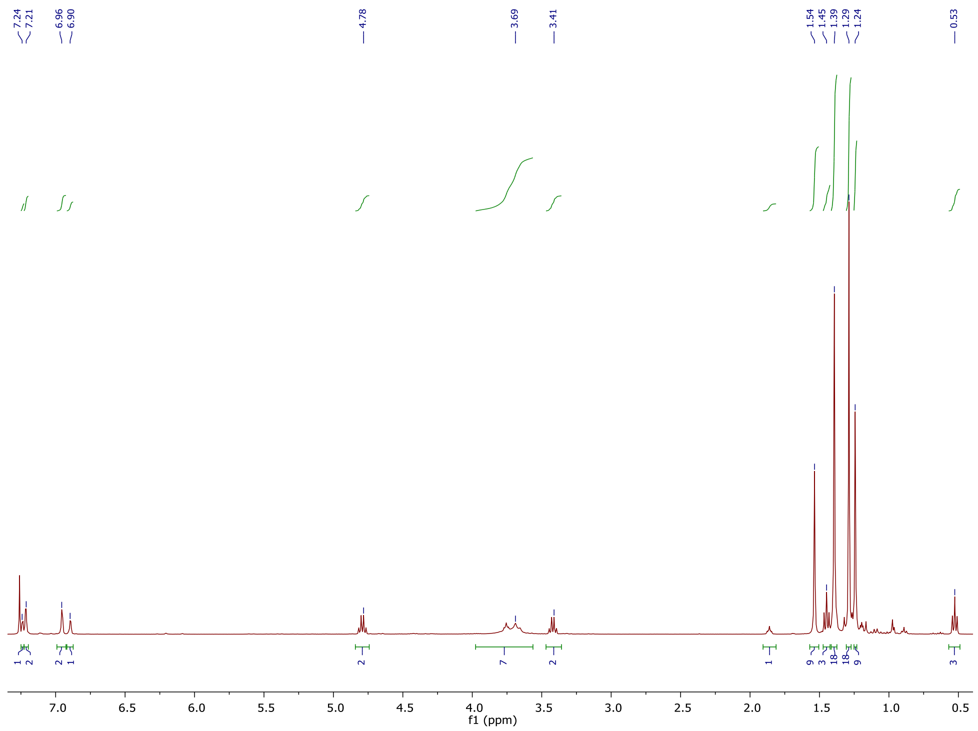

Figure S5. ^{1}H NMR spectrum of **1** in $CDCl_3$ at 298 K

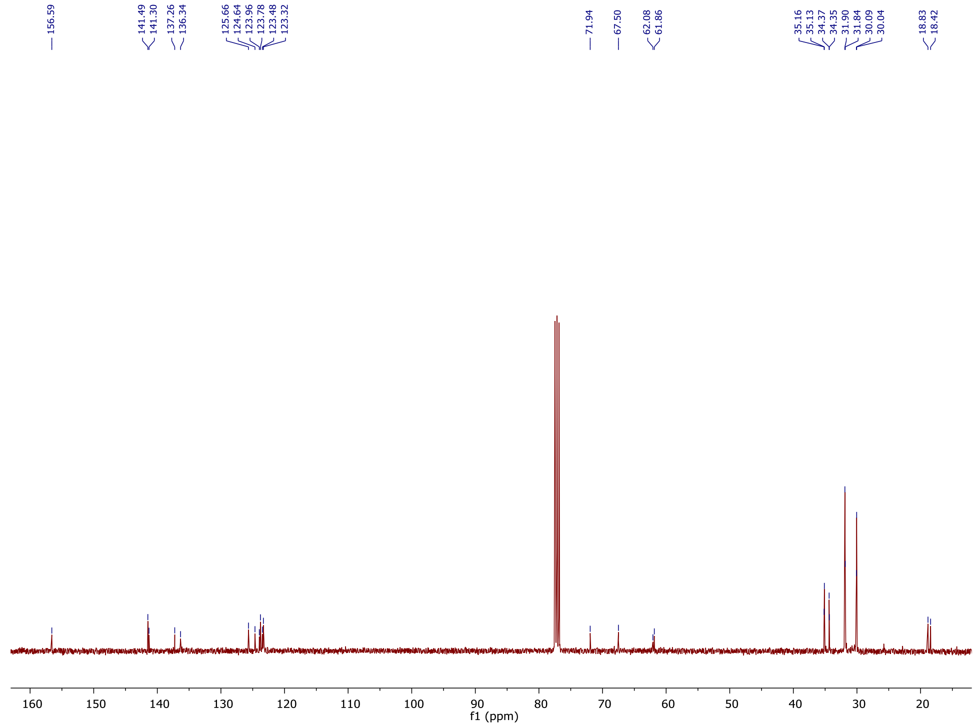

Figure S6. $^{13}C\{^{1}H\}$ NMR spectrum of **1** in $CDCl_3$ at 298 K

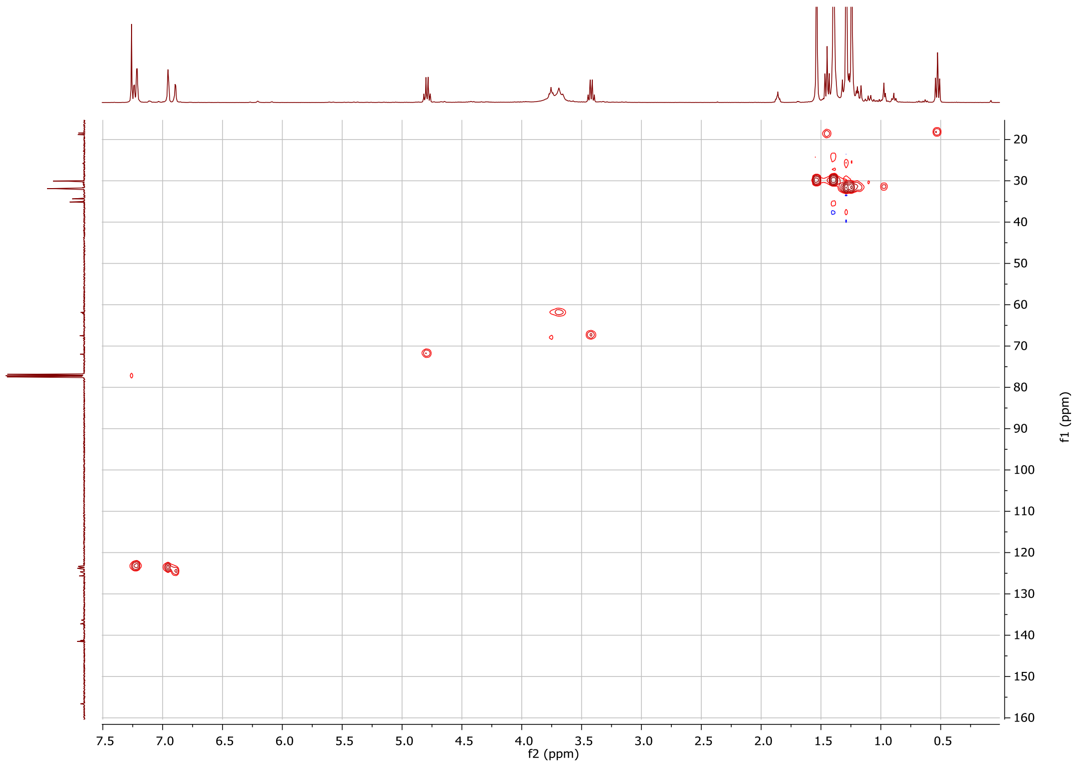

Figure S7. HSQC NMR spectrum of **1** in $CDCl_3$ at 298 K

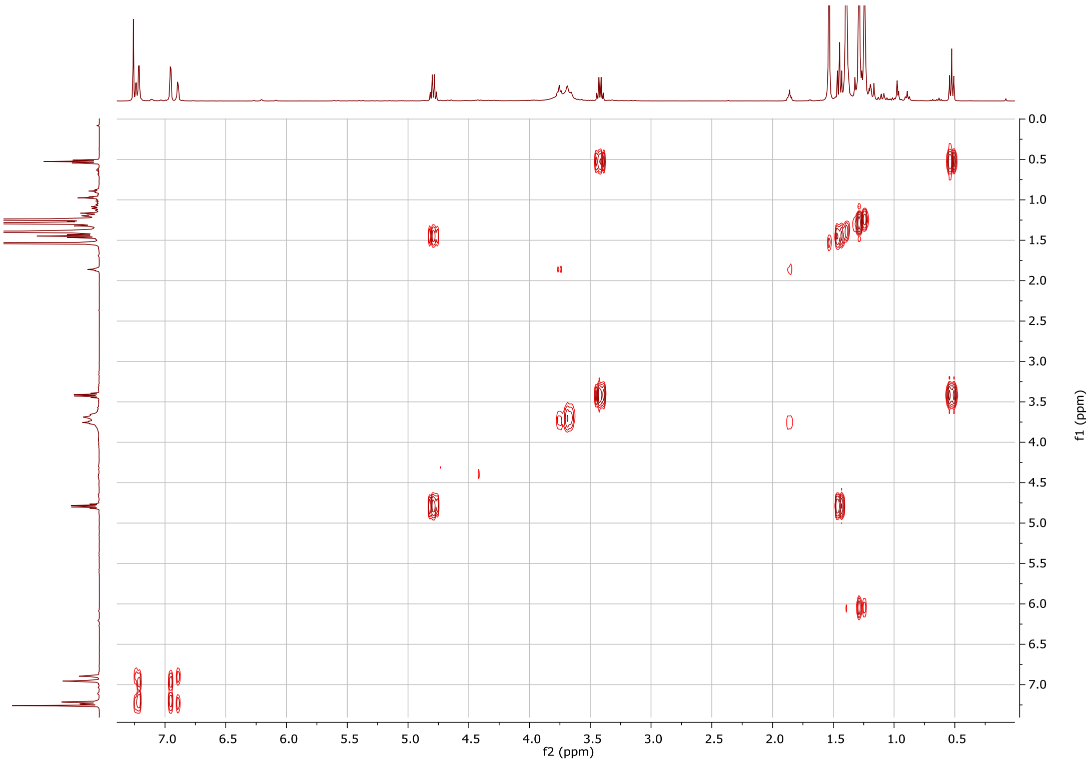

Figure S8. COSY NMR spectrum of **1** in $CDCl_3$ at 298 K, allowing correct assignment of alkoxide CH_3 and CH_2 resonances

Synthesis of Niobium species [L^{tBu}Nb(OEt)Cl], 2

To a solution of **1** (23.5 mmol, 20.0 g, 1 equivalent) in dichloromethane was added excess chlorotrimethylsilane (50 mmol, 6.4 ml, 2 equivalents). Stirring for 24 hours yielded a dark red-brown solution. Solvent and siloxane by-products were removed under dynamic vacuum to yield a yellow solid. The solid was washed sparingly (2 x 30 ml) with hexane and after cannula filtration was dried under dynamic vacuum. Yield: 17.0 g, 86 %. Crystals suitable for diffraction were obtained by recrystallizing from a mixture of THF and hexane.

^{1}H NMR (400 MHz, Toluene-d_8, 298 K, δ_H, ppm); 7.44 (2H, *d*, *J* = 2.5 Hz, *Ar*), 7.36 (1H, *d*, *J* = 2.5 Hz, *Ar*), 6.94 (2H, *d*, *J* = 2.0 Hz, *Ar*), 6.68 (1H, *d*, *J* = 2.0 Hz, *Ar*), 4.82 (2H, *q*, *J* = 7.0 Hz, OCH_2CH_3), 4.26 (2H, *d*, *J* = 13.5 Hz, NCH*H*), 3.42 (2H, *s*, NCH_2), 3.32 (2H, *d*, *J* = 13.5 Hz, NCH*H*), 1.56 (18H, *s*, C(CH_3)$_3$), 1.55 (9H, *s*, C(CH_3)$_3$), 1.44 (3H, *t*, *J* = 7.0 Hz, OCH_2CH_3, *obscured by THF signal*) 1.32 (18H, *s*, C(CH_3)$_3$), 1.25 (9H, *s*, C(CH_3)$_3$). **^{1}H NMR** (400 MHz, $CDCl_3$, 298 K, δ_H, ppm); 7.24 (3H, *m*, *broad*, *Ar*), 6.97 (2H, *s*, *Ar*), 6.91 (1H, *s*, *Ar*), 4.92 (2H, *q*, *J* = 7.1 Hz, OCH_2), 4.25 (2H, *d*, *broad*, *J* = 13.0 Hz, NC*H*H), 3.72 (2H, *s*, NCH_2), 3.63 (2H, *d*, *J* = 13.8 Hz, NC*H*H), 1.56 (3H, *t*, *J* = 7.1 Hz, OCH_2CH_3), 1.47 (9H, *s*, C(CH_3)$_3$), 1.41 (18H, *s*, C(CH_3)$_3$), 1.28 (18H, *s*, C(CH_3)$_3$), 1.22 (9H, *s*, C(CH_3)$_3$). **^{13}C{^{1}H} NMR** (101 MHz, Toluene-d_8, 298 K δ_c, ppm); 156.7 (*Ar*O), 143.6 (*Ar*), 143.1 (*Ar*), 137.1 (*Ar*), 136.7 (*Ar*), 126.1 (*Ar*), 126.1 (*Ar*), 124.6 (*Ar*), 124.3 (*Ar*), 124.0 (*Ar*), 123.9 (*Ar*), 75.5 (OCH_2), 63.7 (NCH_2), 61.0 (NCH_2), 35.5 (*C*(CH_3)$_3$), 35.3 (*C*(CH_3)$_3$), 34.6 (*C*(CH_3)$_3$), 34.5 (*C*(CH_3)$_3$), 31.9 (C(CH_3)$_3$), 31.9 (C(CH_3)$_3$), 30.8 (C(CH_3)$_3$), 30.4 (C(CH_3)$_3$), 18.2 (OCH_2CH_3). **^{13}C{^{1}H} NMR** (101 MHz, $CDCl_3$, 298 K δ_c, ppm); 156.2 (*Ar*O), 156.0 (*Ar*O), 143.6 (*Ar*), 143.5 (*Ar*), 136.8 (*Ar*), 136.7 (*Ar*), 125.7 (*Ar*), 125.3 (*Ar*), 124.6 (*Ar*H), 124.1 (*Ar*H), 123.8 (*Ar*H), 75.4 (OCH_2), 63.4 (NCH_2), 61.4 (NCH_2), 35.2 (*C*(CH_3)$_3$), 35.1 (*C*(CH_3)$_3$), 34.5 (*C*(CH_3)$_3$), 34.5 (*C*(CH_3)$_3$), 31.8 (C(CH_3)$_3$), 31.7 (C(CH_3)$_3$), 30.4 (C(CH_3)$_3$), 30.2 (C(CH_3)$_3$), 18.2 (OCH_2CH_3). **Elemental (CHN) Analysis** (Calculated, for $C_{47}H_{71}NNbO_4Cl$); C: 67.01 %, H: 8.50 %, N: 1.66 %, (Experimental); C: 66.30 %, H: 8.40 %, N: 1.70 %.

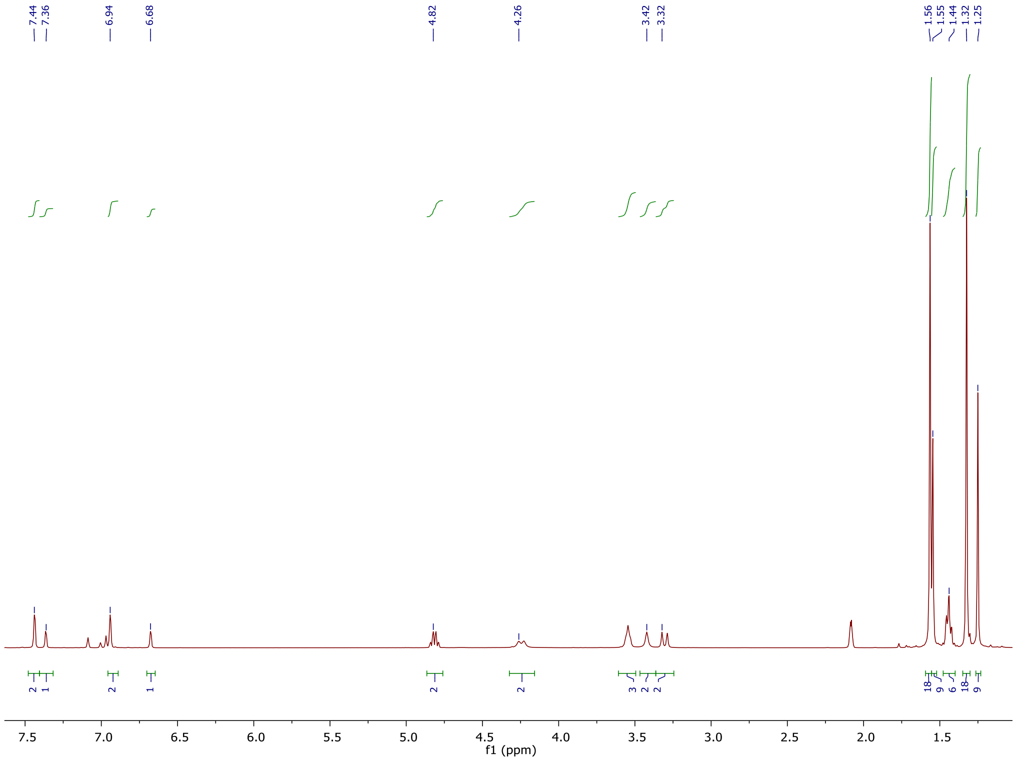

Figure S9. ^{1}H NMR spectrum of **2** in toluene-d_8 at 298 K

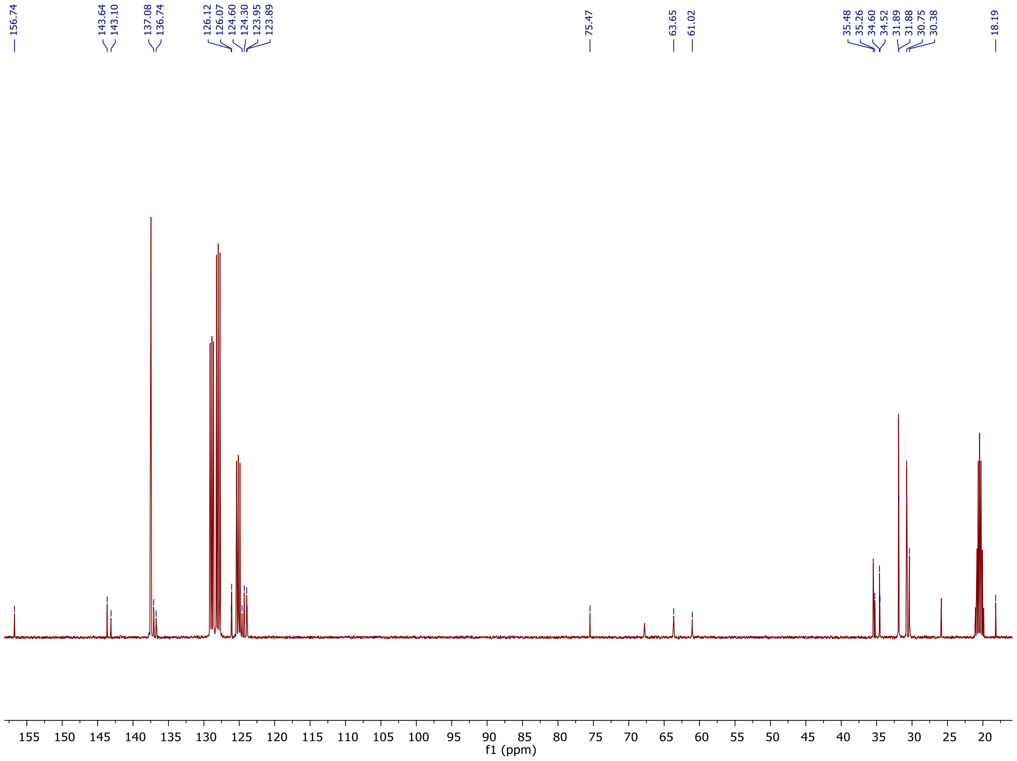

Figure S10. $^{13}C\{^{1}H\}$ NMR spectrum of **2** in toluene-d_8 at 298 K

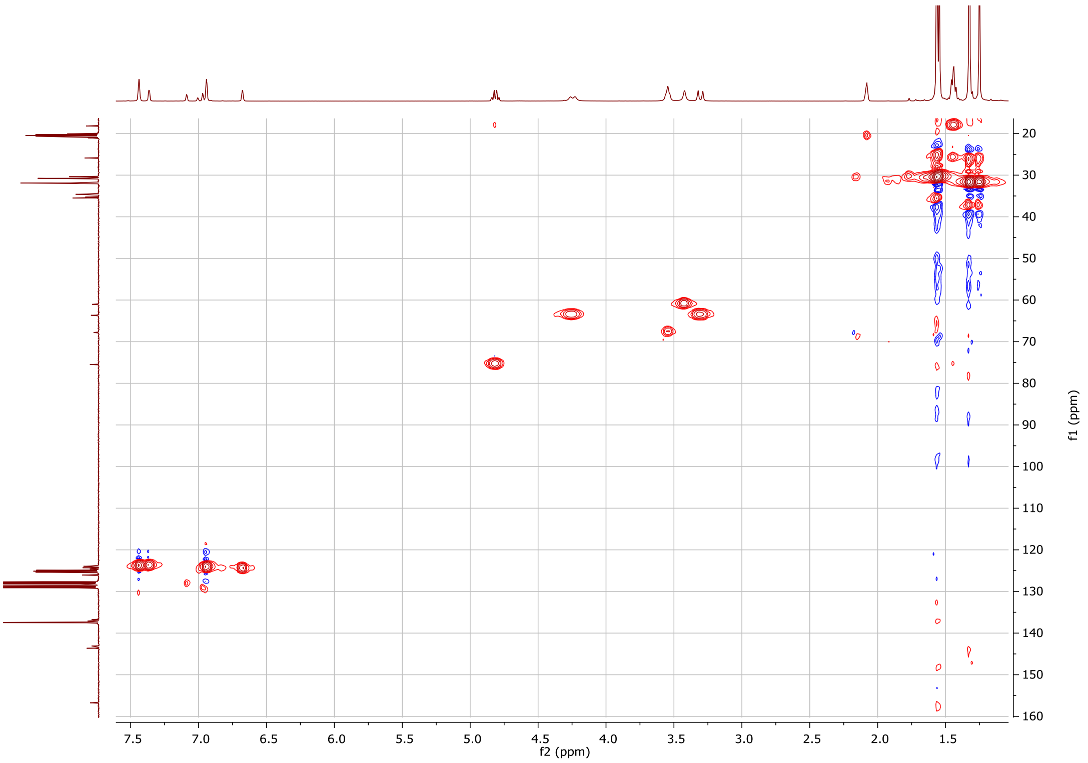

Figure S11. HSQC NMR spectrum of **2** in toluene-d_8 at 298 K

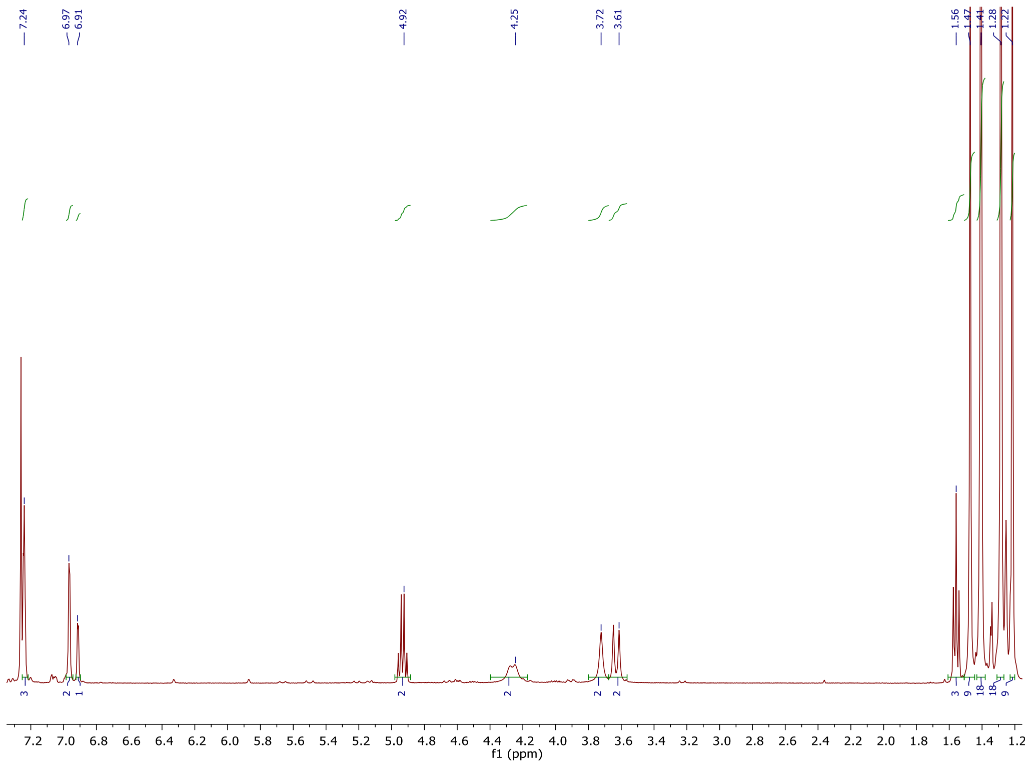

Figure S12. ^{1}H NMR spectrum of **2** in $CDCl_3$ at 298 K

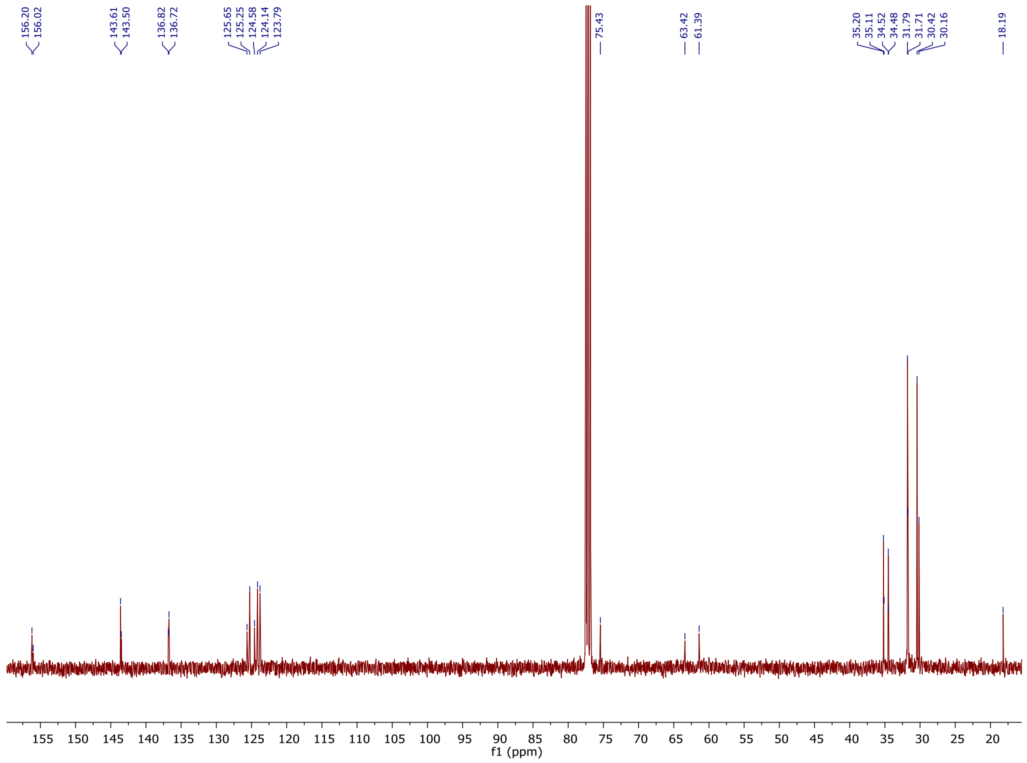

Figure S13. $^{13}C\{^{1}H\}$ NMR spectrum of **2** in $CDCl_3$ at 298 K

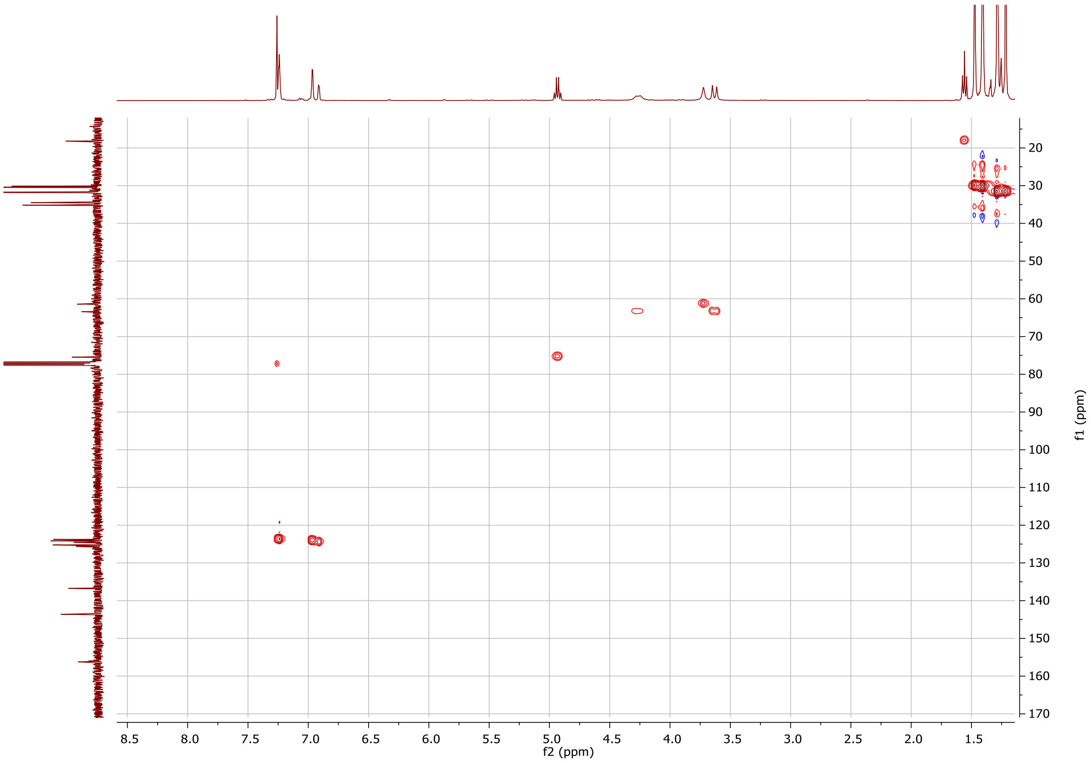

Figure S14. HSQC NMR spectrum of **2** in $CDCl_3$ at 298 K

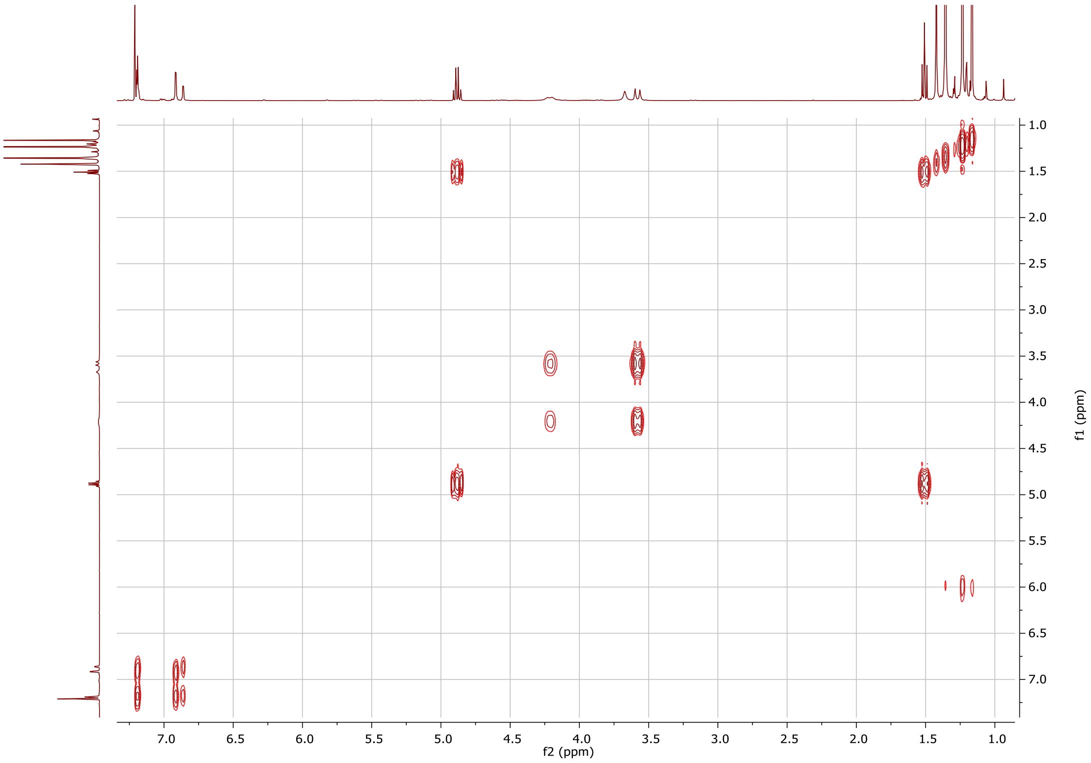

Figure S15. COSY NMR spectrum of **2** in $CDCl_3$ at 298 K

Synthesis of Niobium species $[\{L^{tBu}Nb(OEt)\}\text{-}\mu_2F\text{-}\{L^{tBu}NbF\}]^+[SbF_6]^-$, 3

In a 30 ml vial in the glove box, a sample of complex **2** (0.60 mmol, 0.5 g, 1 equivalent), was dissolved in dry toluene (10 ml). To this was added $AgSbF_6$ (0.90 mmol, 0.31 g, 1.5 equivalents). The vial was sealed and shaken vigorously for 30 seconds, precipitating AgCl. The mixture was then filtered twice through a 0.2 µm PTFE syringe filter, into a clean 30 ml vial, which was then sealed. The resulting orange-red solution was allowed to stand in the glove box for 20 hours, after which a deep red crystalline solid had formed. The solution phase was removed using a syringe and hypodermic needle, and the solid washed with hexane (2 x 20 ml), which was also removed with a syringe and hypodermic needle. The solid was then transferred to a Schlenk flask and dried under dynamic vacuum for 1 hour. Yield: 0.43 g, 78 %. Crystals suitable for diffraction were obtained on reaction of **2** and $AgSbF_6$ in toluene-d_8 following an analogous procedure. Efforts to obtain NMR data in THF-d_8 were unsuccessful due to polymerisation of the solvent on addition to the crystalline material.

*The synthesis of the trifluorinated analogue of **3**, $[\{L^{tBu}NbF\}_2\text{-}\mu_2F]^+[SbF_6]^-$, **3a**, was attempted. The synthetic procedure was identical to that described for the preparation of **3**, except 2.33 equivalents of $AgSbF_6$ were used, instead of 1.5 equivalents. Although a crystal structure of **3a** was obtained (Figure S35), the bulk material appeared to consist primarily of **3**. High resolution mass spectrometry (not carried out under air- or moisture-free conditions) confirmed that **3a**, which lacks an alkoxide initiating group, was not a significant contaminant of **3**. The spectra contained many unidentifiable species, attributed to decomposition of **3** due to use of 'wet' solvent (MeCN) under ambient air. However, qualitative comparison of the residual concentrations of **3**, bis-fluoro complex **3a** and a bis-alkoxide species, **3b**, which was also detectable, was possible. In conjunction with satisfactory elemental (CHN) analysis data, this is indicative of a satisfactorily selective synthetic route to **3**.*

^{1}H NMR (400 MHz, Toluene-d_8, 223 K, 128 Scans, δ_H, ppm);* 7.61 (2H, *s*, *Ar*), 7.58 (2H, *s*, *Ar*), 7.41 (2H, *s*, *Ar*), 6.85 (4H, *broad*, *Ar*), 6.79 (2H, *broad*, *Ar*), 4.89 (2H, *d*, *broad*, *J* = 13.5 Hz, NC*H*H), 4.29 (2H, *d*, *broad*, *J* = 13.0 Hz, NC*H*H), 3.76 (2H, *d*, *broad*, *J* = 13.0 Hz, NC*H*H), 3.41 (2H, *broad*, OCH_2CH_3), 2.83 (2H, *d*, *broad*, *J* = 13.5 Hz, NC*H*H), 2.42 (2H, *d*, *broad*, *J* = 13.0 Hz, NC*H*H), 2.30 (2H, *d*, *broad*, *J* = 13.0 Hz, NC*H*H), 1.66 (36H, *s*, $C(CH_3)_3$), 1.51 (18H, *s*, $C(CH_3)_3$), 1.43 (18H, *s*, $C(CH_3)_3$), 1.39 (18H, *s*, $C(CH_3)_3$), 1.31 (18H, *s*, $C(CH_3)_3$).** **^{1}H NMR** (400 MHz, C_6D_6, 283 K, δ_H, ppm); 7.55 (2H, *d*, *J* = 2.0 Hz, *Ar*), 7.50 (4H, *s*, *Ar*), 6.85 (4H, *s*, *Ar*), 6.82 (2H, *d*, *J* = 2.0 Hz, *Ar*), 3.56 (2H, *q*, *J* = 7.0 Hz, OCH_2CH_3), 3.34 (8H, *broad*, NCH_2), 2.98 (4H, *broad*, NCH_2) 1.62-1.69 (21H, *m*, *broad*, $C(CH_3)_3$ *and* OCH_2CH_3), 1.57 (27H, *m*, $C(CH_3)_3$), 1.34-1.39 (18H, *m*, *broad*, $C(CH_3)_3$), 1.32 (36H, *s*, $C(CH_3)_3$), 1.21(18H, *s*, $C(CH_3)_3$).**^{1}H NMR** (400 MHz, $CDCl_3$, 233 K, 64 Scans, δ_H, ppm); 7.39-6.98 (12H, *Ar*), 4.76 (2H, *broad*, NC*H*H), 4.48 (2H, *broad*, NC*H*H), 3.99 (4H, *broad*, NC*H*H *and* OCH_2CH_3), 3.46 (2H, *broad*, NC*H*H), 3.32 (2H, *broad*, NC*H*H), 3.15 (2H, *broad*, NC*H*H), 1.05-1.60 (111H, *m*, *broad*, $C(CH_3)_3$ *and* OCH_2CH_3).*** **^{1}H NMR** (500 MHz, $CDCl_3$, 298 K, 128 Scans, δ_H, ppm); 7.35 (2H, *d*, *J* = 2.0 Hz, *Ar*), 7.28 (4H, *d*, *J* = 2.0 Hz, *Ar*), 7.06 (2H, *d*, *J* = 2.0 Hz, *Ar*), 7.00 (4H, *d*, *J* = 2.0 Hz, *Ar*), 5.07 (2H, *q*, *J* = 7.0 Hz, OCH_2CH_3), 3.89 (6H, *broad*, NCH*H*), 3.71 (6H, *broad*, NCH*H*), 1.61 (3H, *t*, *J* = 7.0 Hz, OCH_2CH_3), 1.54 (18H, *s*, $C(CH_3)_3$), 1.38 (36H, *s*, $C(CH_3)_3$), 1.27 (18H, *s*, $C(CH_3)_3$), 1.27 (36H, *s*, $C(CH_3)_3$).**** **^{13}C{^{1}H} NMR**; Acquisition of ^{13}C{^{1}H} NMR data was precluded by the extremely low solubility of **3** in the all solvents that were used, and the need for low temperature conditions. **^{19}F NMR** (376 MHz, $CDCl_3$, 233 K, δ_F, ppm); -117.19 (1F, *broad*, Nb*F* *or* Nb*F*Nb), -117.49 (1F, *broad*, Nb*F* *or* Nb*F*Nb), -122.54 (5F, *broad*, SbF_6).***** **Elemental (CHN) Analysis** (Calculated, for $C_{92}H_{137}F_8N_2Nb_2O_7Sb$); C: 59.97 %, H: 7.49 %, N: 1.52 %, (Experimental); C: 60.20 %, H: 7.63 %, N: 1.49 %.

ESI-MS (m/z): 1605.8555; calc. for $[C_{92}H_{137}F_2N_2Nb_2O_7]^+$ (***3***): 1605.8516.

** Large residual solvent signals, visible due to the extremely low solubility of **3**, and the presence of lattice toluene, have not been reported*

*** Ethoxide CH_3 ^{1}H NMR resonances not detected in toluene-d_8. A broad signal corresponding to the ethoxide CH_2 protons was detected, and a cross-peak in the relevant COSY spectrum suggested the CH_3*

protons had a chemical shift of δ~0.38 ppm, suggesting the ethoxide environment is highly fluxional. However, the acquisition of spectra at low temperature allowed resolution of NCH_2 resonances. 6 NCH*H environments were distinguished, which we suggest correspond to three of each axial and equatorial proton environments, with the NCH_2 environments of the two ligand residues of the dinuclear complex **3** being sufficiently similar as to be indistinguishable from their respective chemical shifts. The COSY spectrum and the magnitude of the coupling constant, J, for each* NCH*H signal support the presence of axial-equatorial germinal coupling. The fluxionality of ^1H environments may correspond to the bimetallic cation of **3** being in equilibrium with other bimetallic cationic species in solution, $[\{L^{tBu}Nb(OEt)\}_2\text{-}\mu_2F]^+$ and $[\{L^{tBu}NbF\}_2\text{-}\mu_2F]^+$ due to lability of the two Nb-μ_2F bonds permitting dissociation of **3**, into cationic and neutral fragments, $[L^{tBu}Nb(OEt)]^+$, $[L^{tBu}NbF]^+$, $[L^{tBu}Nb(OEt)F]$, $[L^{tBu}NbF_2]$. However, only very small amounts of $[\{L^{tBu}Nb(OEt)\}_2\text{-}\mu_2F]^+$ and $[\{L^{tBu}NbF\}_2\text{-}\mu_2F]^+$ were detected by ESI-MS, relative to $[\{L^{tBu}Nb(OEt)\}\text{-}\mu_2F\text{-}\{L^{tBu}NbF\}]^+$.*

**** Aromatic and methyl regions poorly resolved, and therefore could not be assigned in full for the low-temperature spectrum in $CDCl_3$, attributed to fluxional behaviour and low solubility.*

***** At 298 K, the ethoxide CH_2 and CH_3 protons could be clearly distinguished by ^1H NMR in $CDCl_3$, and confirmed by COSY. However, the appearance of additional signals in the $C(CH_3)_3$ and NCH_2 regions of the spectrum suggests **3** is highly fluxional in solution. Additional $C(CH_3)_3$ signals were also present in the ^1H NMR spectrum of **3** in C_6D_6 at 298 K. The appearance of the ethoxide CH_2 shift at much higher shift than at low temperature, or in less polar solevnts, suggests reversible Nb-F bond cleavage occurred in $CDCl_3$ at ambient temperature.*

****** Accurate signal integration prevented by broad, weak ^{19}F NMR signals.*

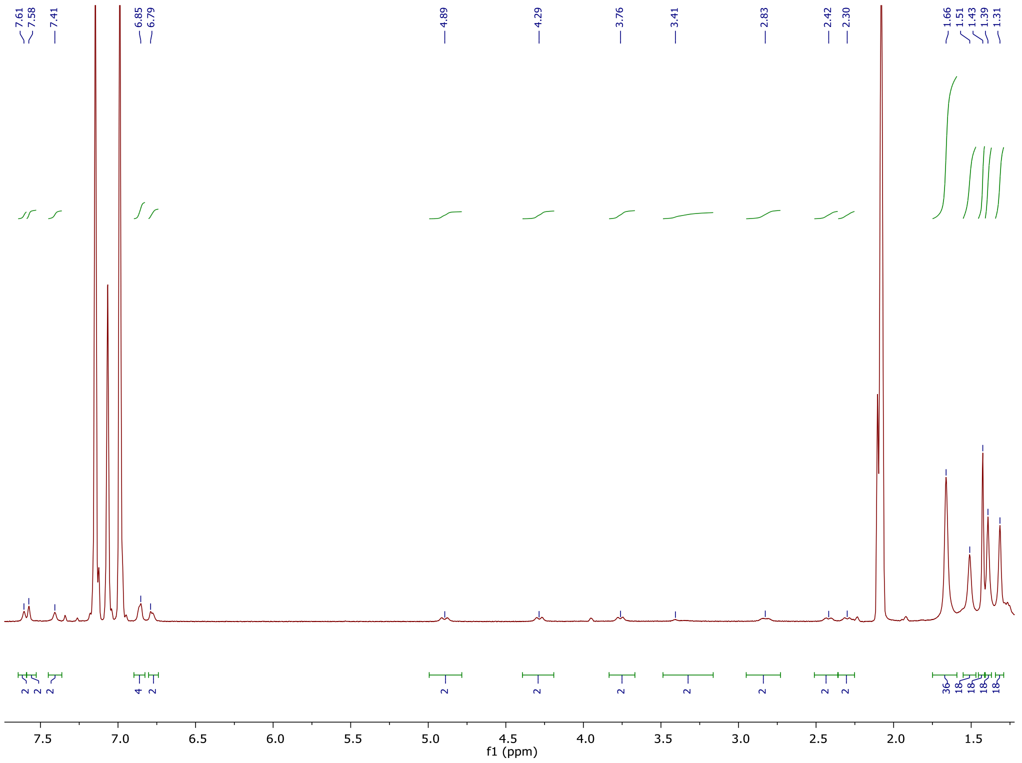

Figure S16. ^1H NMR spectrum of **3** in toluene-d_8 at 223 K

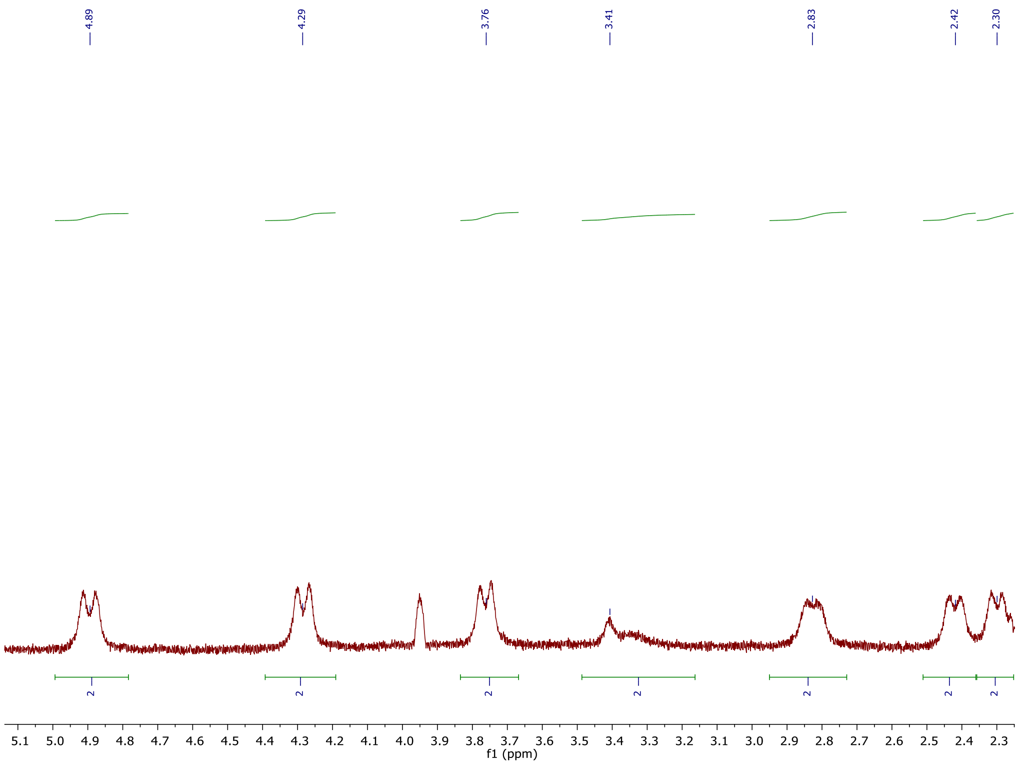

Figure S17. The methylene region of the ^{1}H NMR spectrum of **3** in toluene-d_8 at 223 K

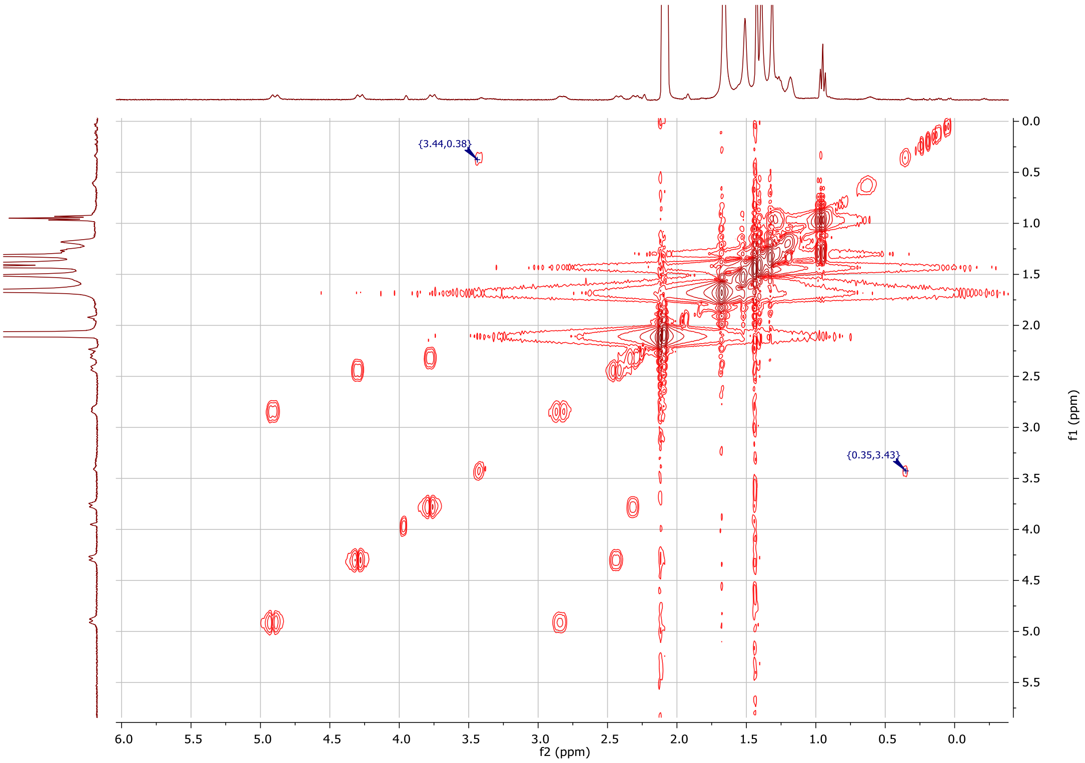

Figure S18. COSY NMR spectrum of **3** in toluene-d_8 at 223 K

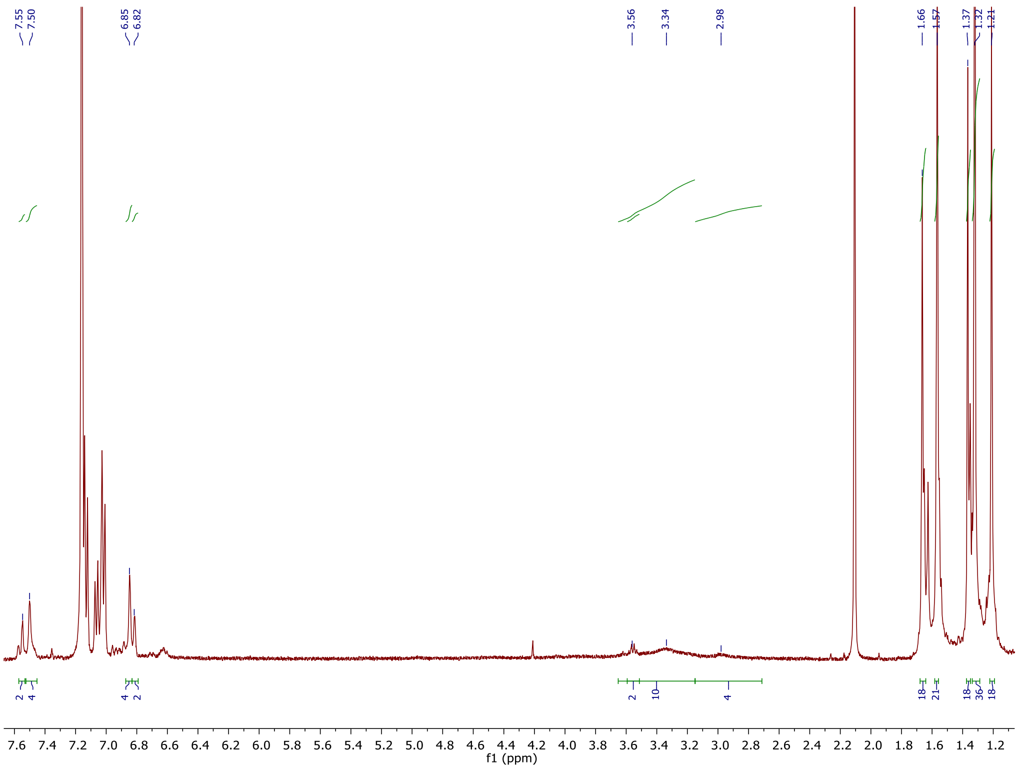

Figure S19. ^{1}H NMR spectrum of **3** in C_6D_6 at 283 K

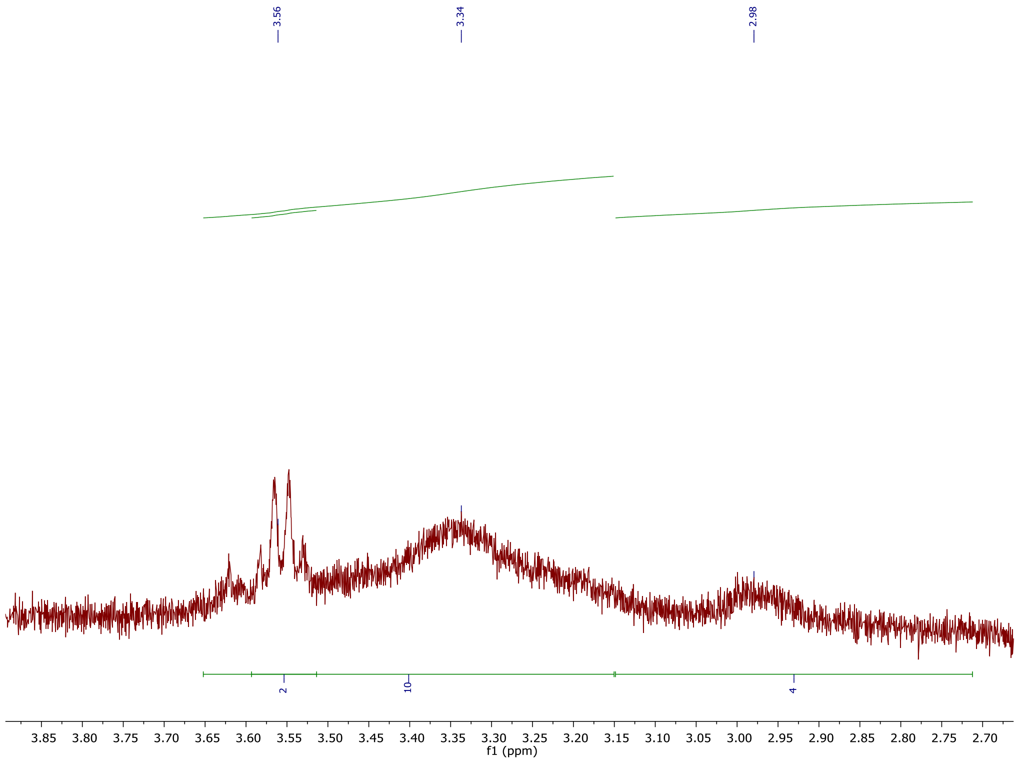

Figure S20. The methylene region of the ^{1}H NMR spectrum of **3** in C_6D_6 at 283 K, showing coalescence of $NC\mathit{H_2}$ signals, and resolution of alkoxide $OC\mathit{H_2}$ quartet resonance

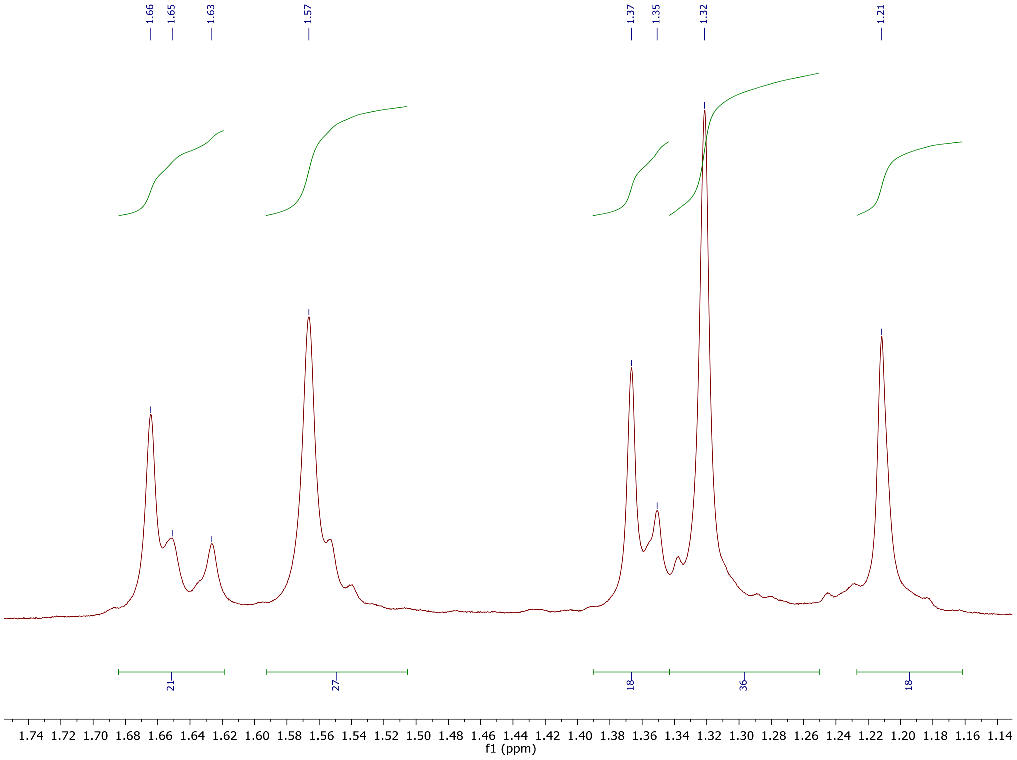

Figure S21. Methyl region of the ^1H NMR spectrum of **3** in C_6D_6 at 283 K

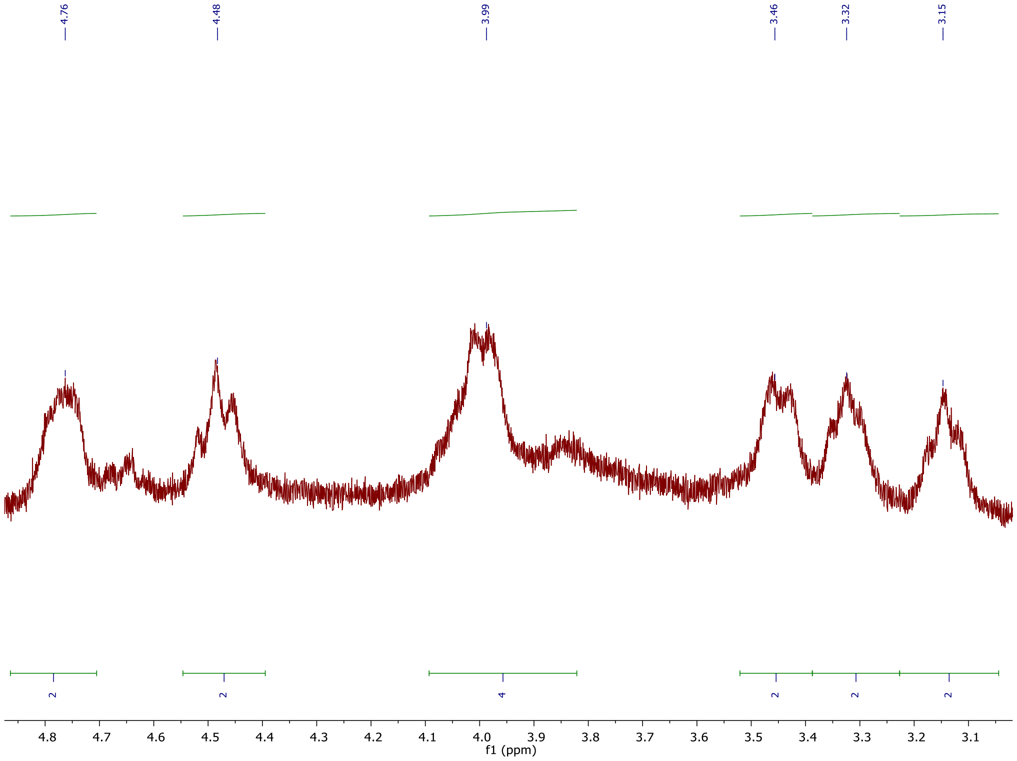

Figure S22. Methylene region of the ^1H NMR spectrum of **3** in $CDCl_3$ at 233 K

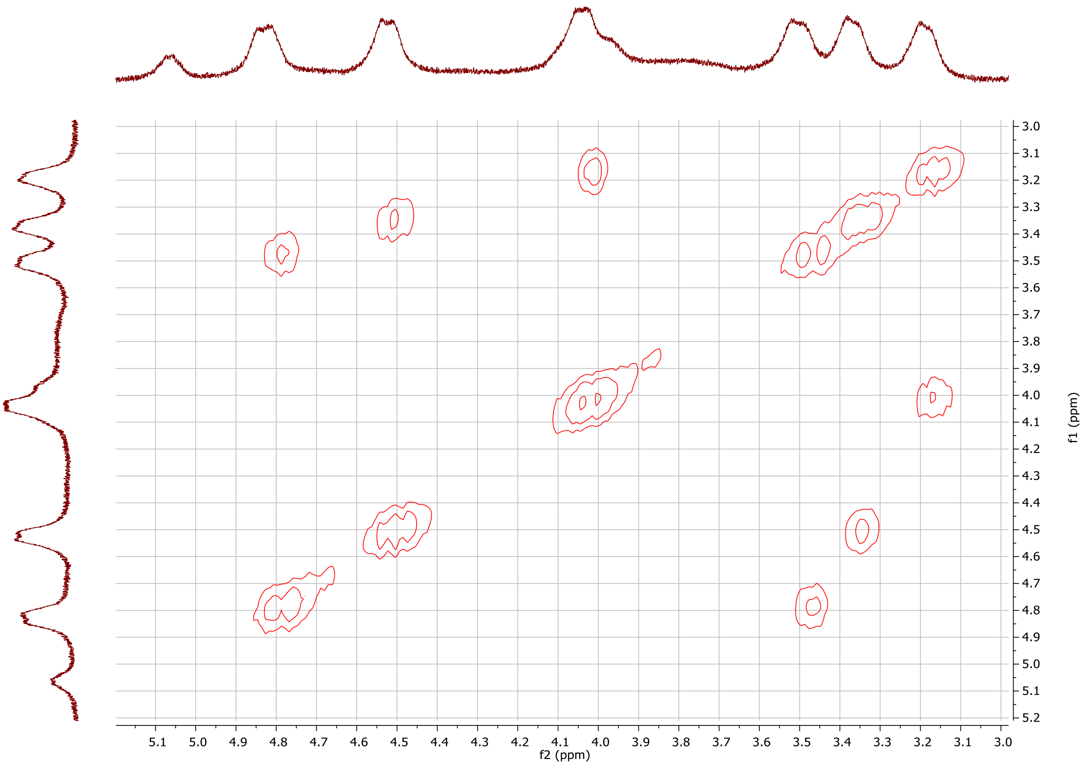

Figure S23. Methylene region of the COSY NMR spectrum of **3** in $CDCl_3$ at 233 K

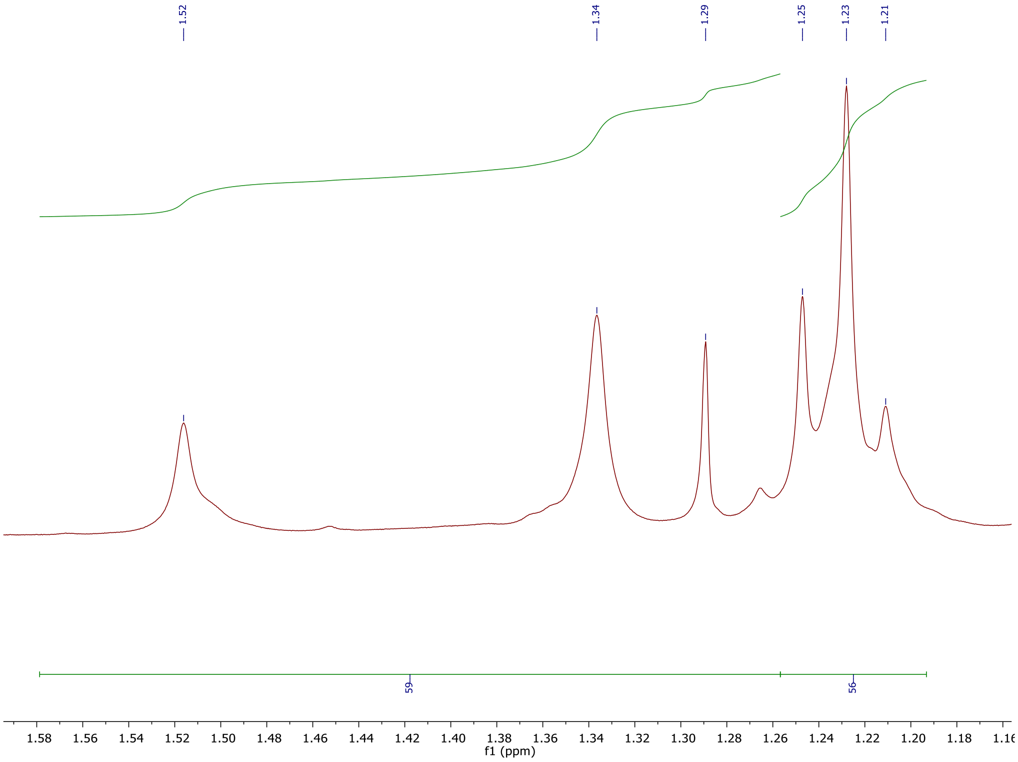

Figure S24. Methyl region of the ^1H NMR spectrum of **3** in $CDCl_3$ at 233 K

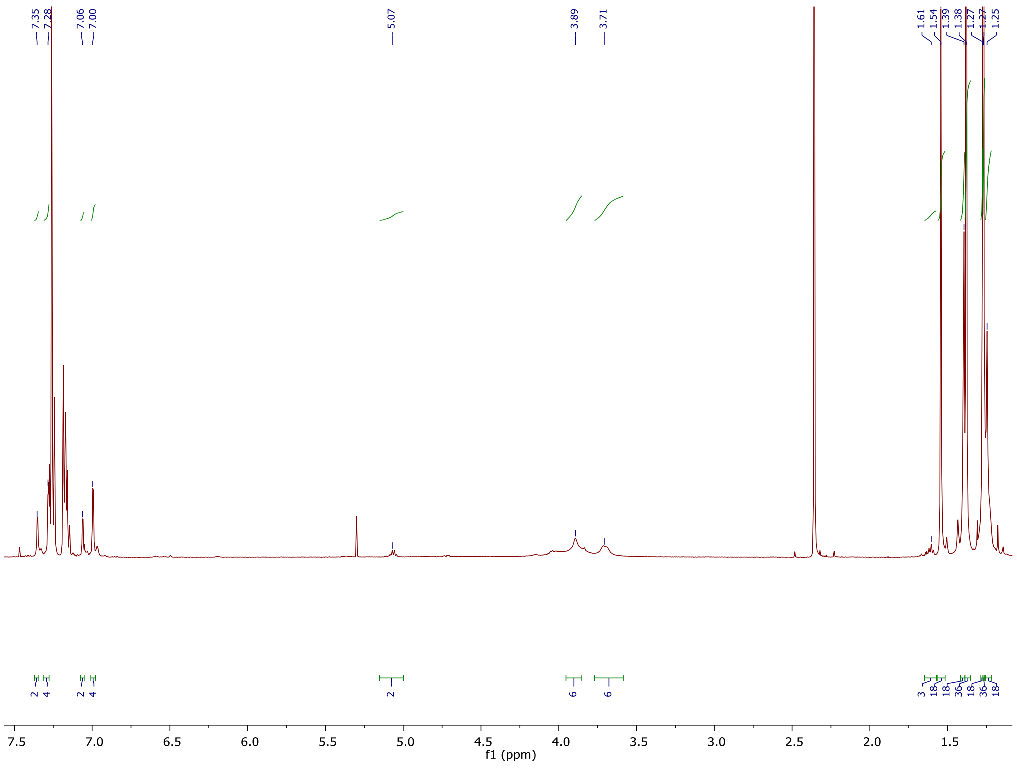

Figure S25. ^{1}H NMR spectrum of **3** in $CDCl_3$ at 298 K

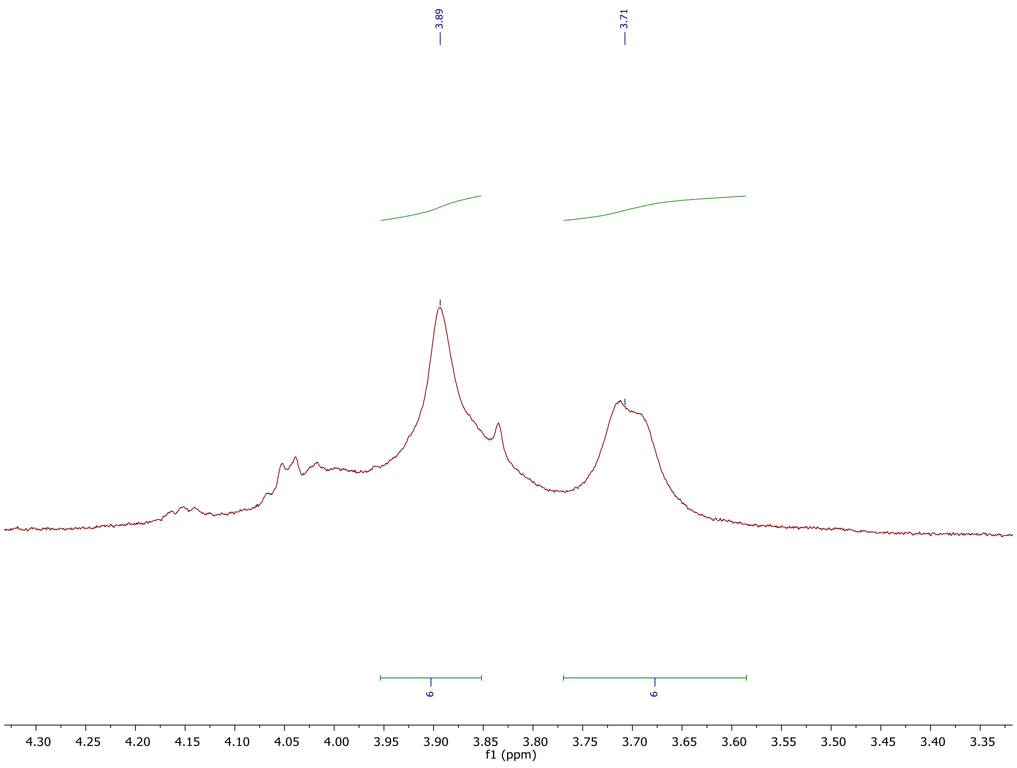

Figure S26. Methylene region of the ^{1}H NMR spectrum of **3** in $CDCl_3$ at 298 K

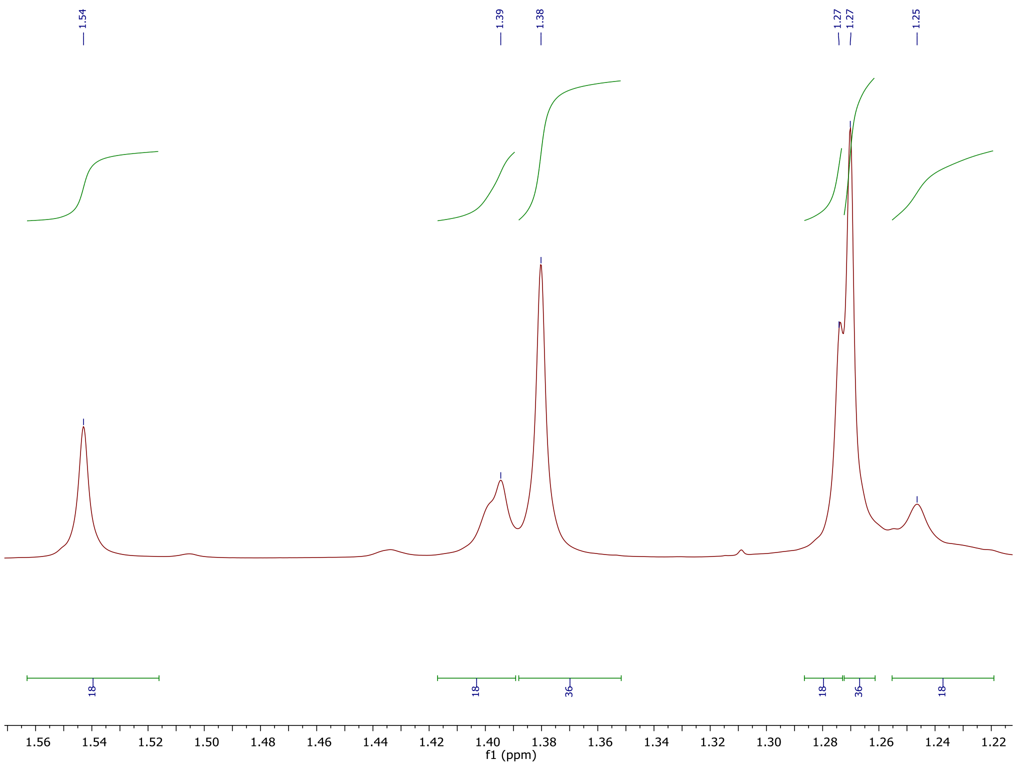

Figure S27. Methyl region of the ^{1}H NMR spectrum of **3** in $CDCl_3$ at 298 K

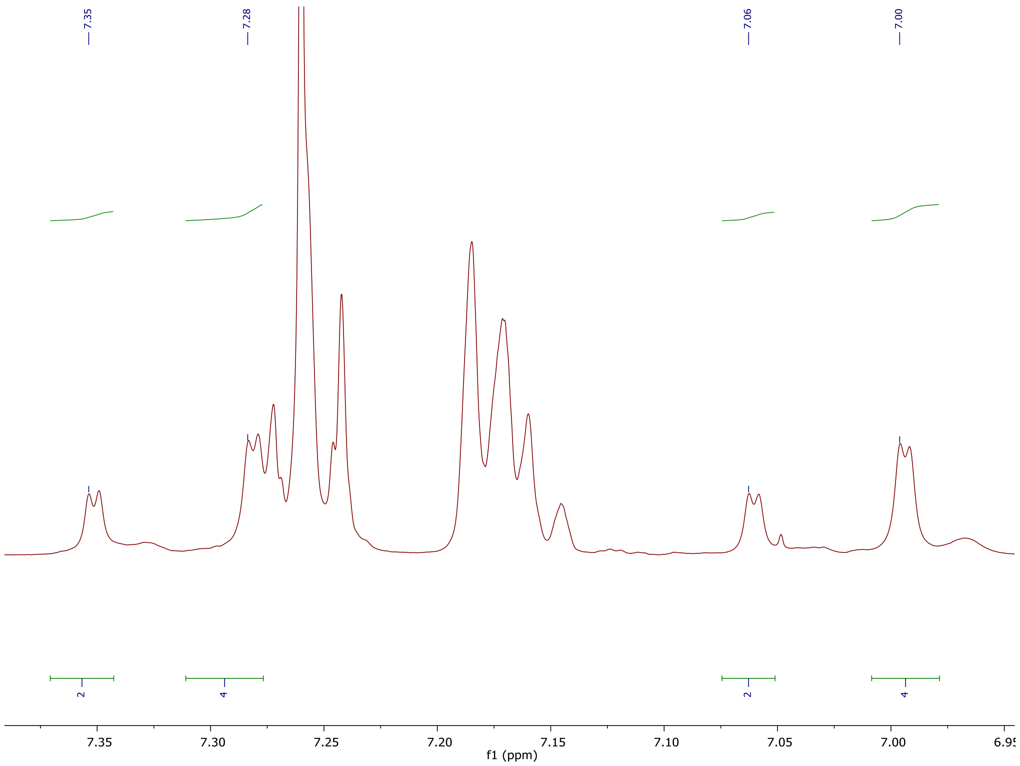

Figure S28. Aromatic region of the ^{1}H NMR spectrum of **3** in $CDCl_3$ at 298 K

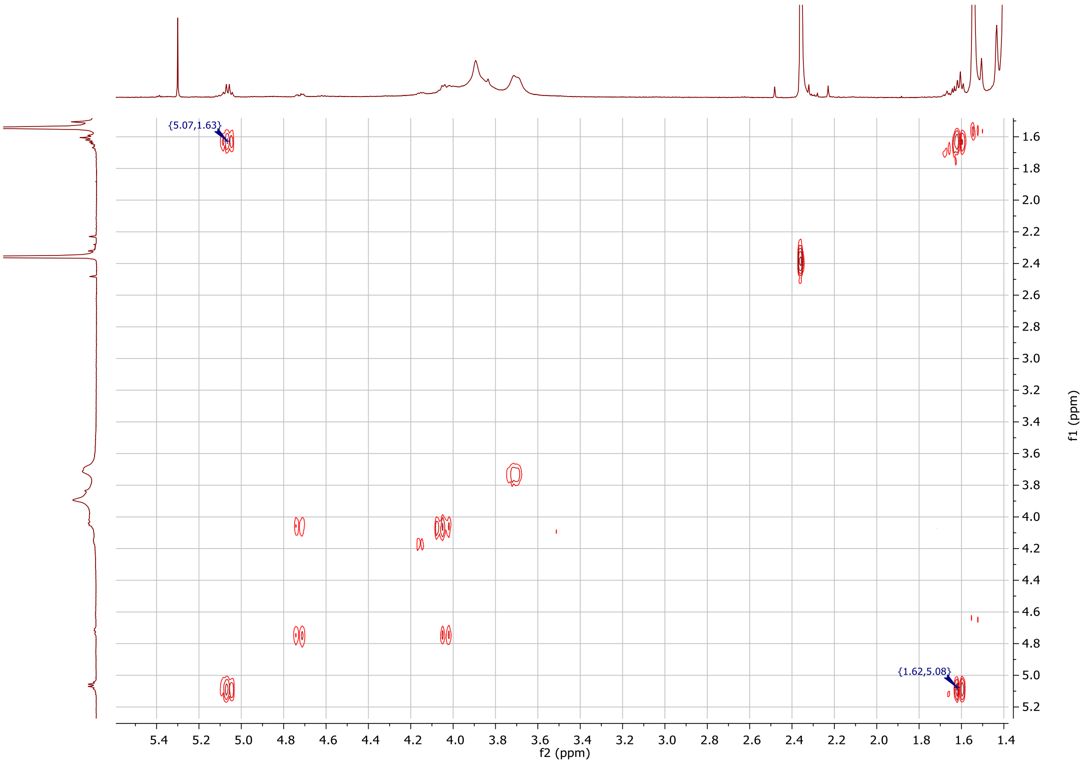

Figure S29. COSY NMR spectrum of **3** in $CDCl_3$ at 298 K

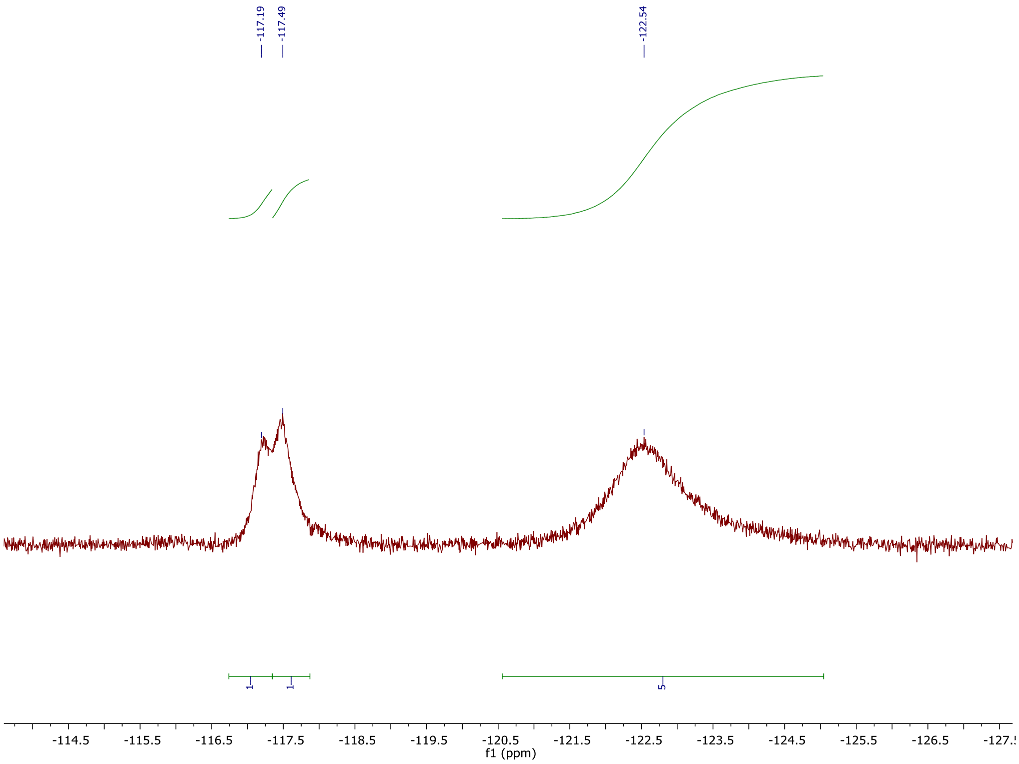

Figure S30. ^{19}F NMR spectrum of **3** in $CDCl_3$ at 233 K

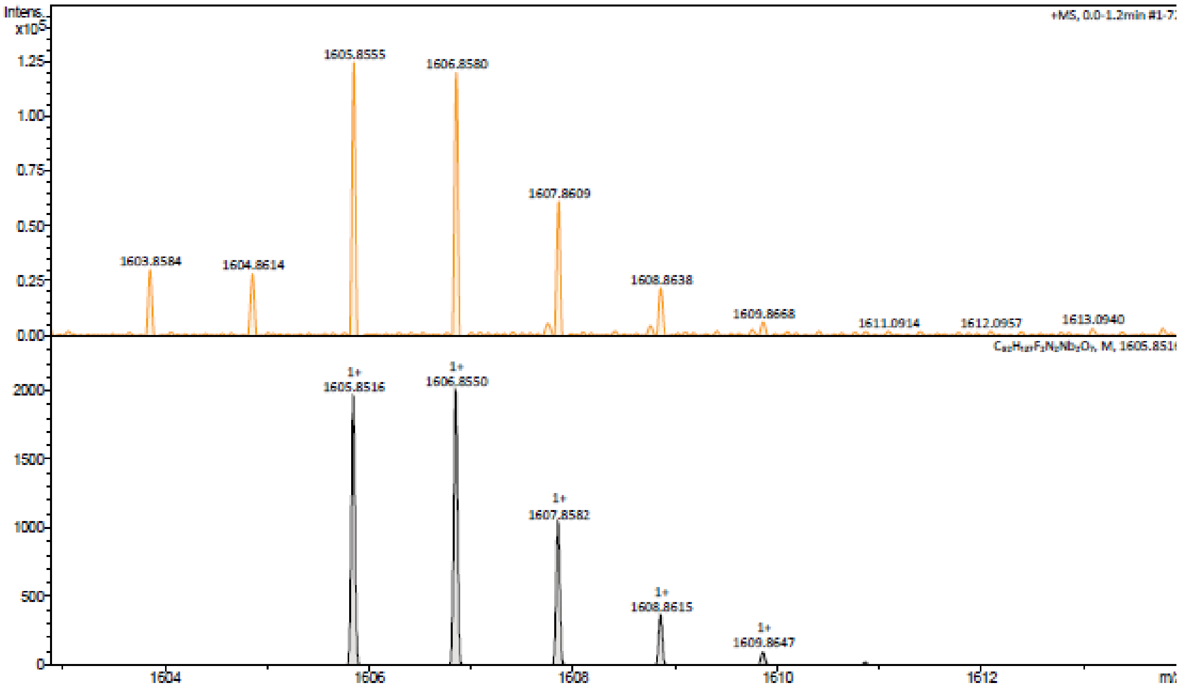

Figure S31. High-resolution mass spectrum isotope pattern (top) confirming presence of the cation of **3**, $[\{L^{tBu}Nb(OEt)\}\text{-}\mu_2F\text{-}\{L^{tBu}NbF\}]^+$ against simulated spectrum (bottom).

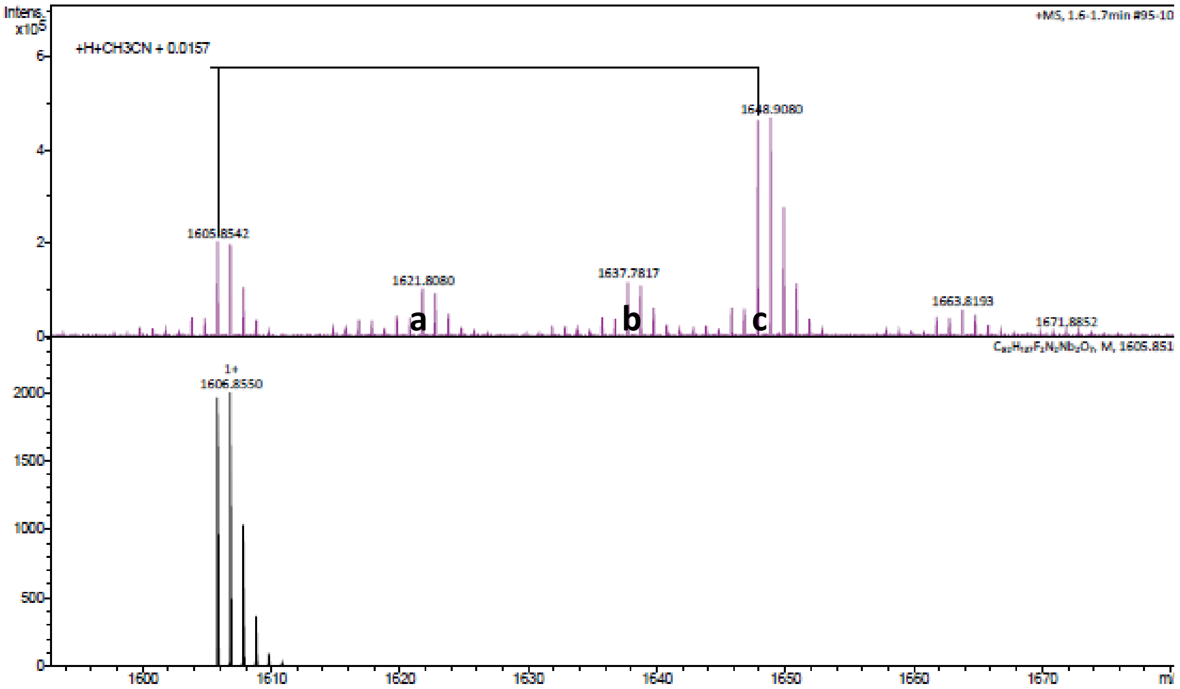

Figure S32. High-resolution mass spectrum detail (top) confirming presence of $[\{L^{tBu}Nb(OEt)\}\text{-}\mu_2F\text{-}\{L^{tBu}NbF\}]^+$ adducts $[\{L^{tBu}Nb(OEt)\}\text{-}\mu_2F\text{-}\{L^{tBu}NbF\}]^+[O]$, **a**, $[\{L^{tBu}Nb(OEt)\}\text{-}\mu_2F\text{-}\{L^{tBu}NbF\}]^+[O]_2$, **b**, and $[\{L^{tBu}Nb(OEt)\}\text{-}\mu_2F\text{-}\{L^{tBu}NbF\}]^+[H][CH_3CN]$, **c**, against simulated spectrum (bottom).

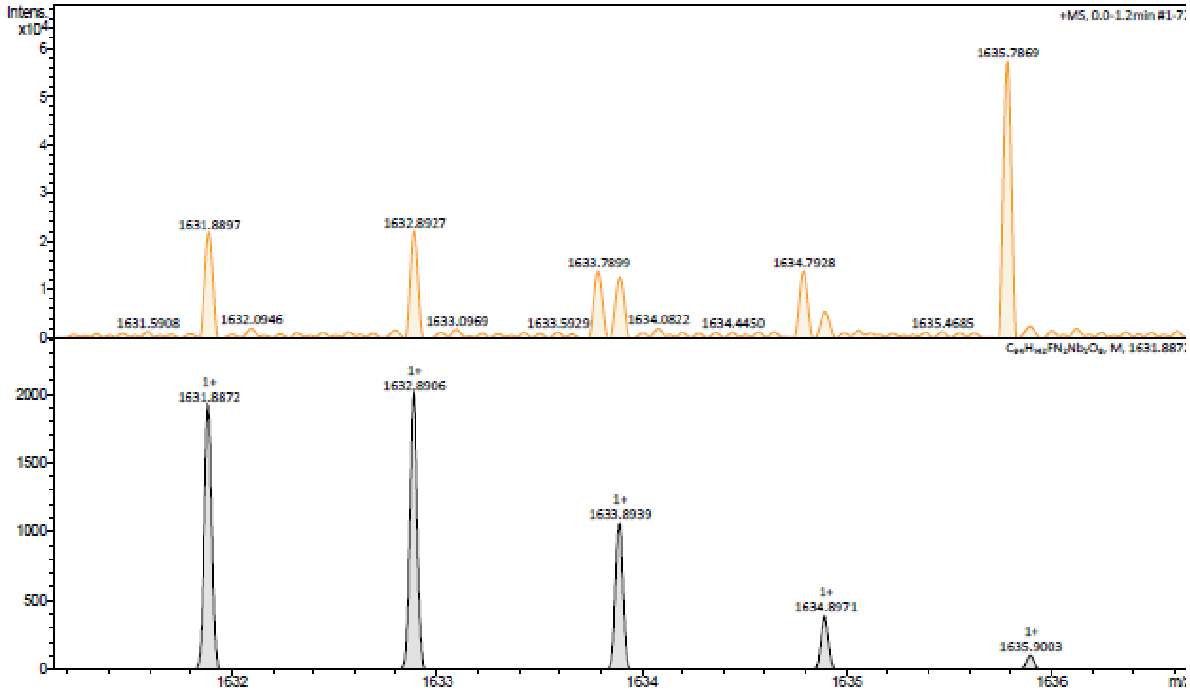

Figure S33. High-resolution mass spectrum detail (top) showing low relative abundance of [{L^{tBu}Nb(OEt)}$_2$-μ$_2$F]$^+$ in sample of **3**, indicating selectivity of the synthetic route with respect to fluorination of the dinuclear monocationic species. Simulated spectrum of [{L^{tBu}Nb(OEt)}$_2$-μ$_2$F]$^+$ at bottom.

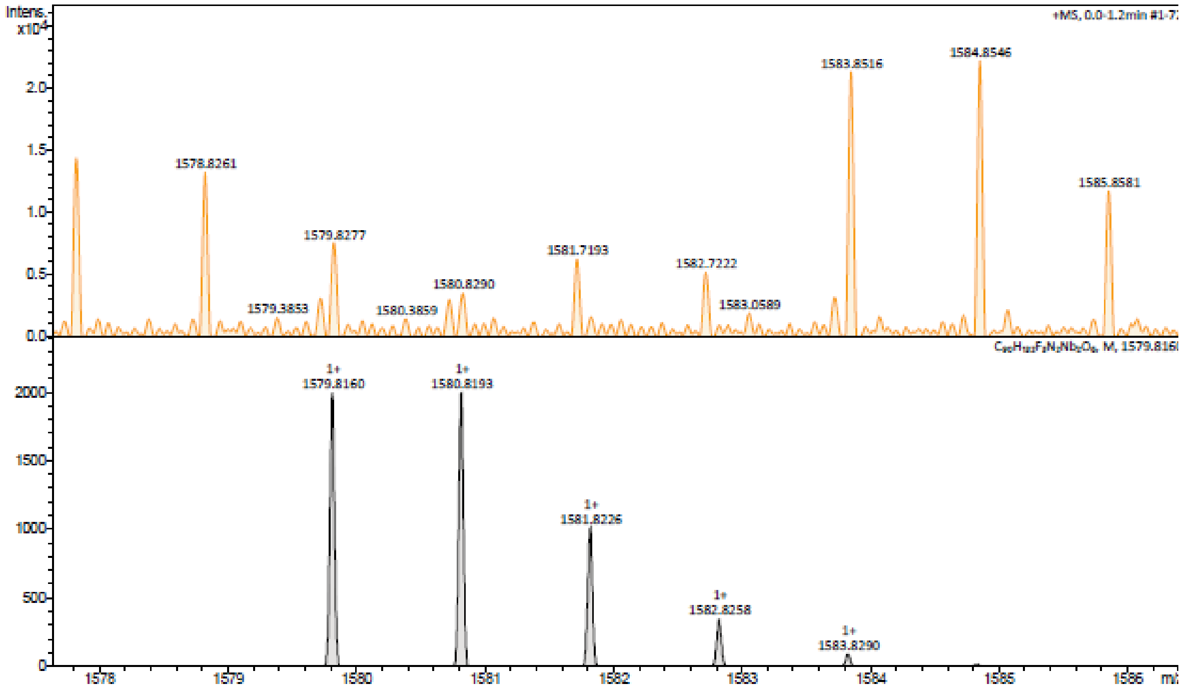

Figure S34. High-resolution mass spectrum detail (top) showing very low relative abundance of [{L^{tBu}NbF}$_2$-μ$_2$F]$^+$, which doesn't bear an alkoxide initiating group, in sample of **3**, indicating selectivity of synthetic route with respect to fluorination of the dinuclear monocationic species. Simulated spectrum of [{L^{tBu}NbF}$_2$-μ$_2$F]$^+$ at bottom.

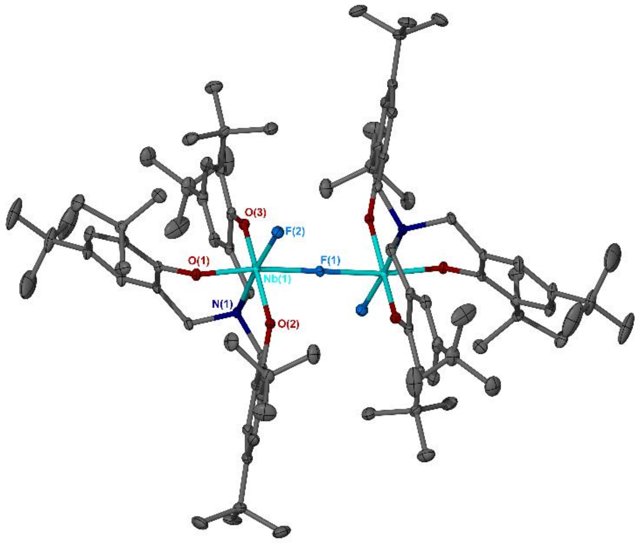

Figure S35. The solid-state structure of the bimetallic, monocationic fragment of Nb(V) species $[\{L^{tBu}NbF\}_2\text{-}\mu_2F]^+[SbF_6]^-$, **3a**. Ellipsoids shown at 30% probability level. Hydrogen atoms, lattice solvent, and SbF_6^- anion have been omitted for clarity. The complete molecule was generated by symmetry. Selected bond lengths (Å) and angles (°): Nb(1)-F(1) 2.0998(3), Nb(1)-F(2) 1.871(2), Nb(1)-N(1) 2.323(3), N(1)-Nb(1)-F(2) 173.07(9), O(1)-Nb(1)-F(1) 170.14(8), O(1)-Nb(1)-F(2) 100.91(10), O(2)-Nb(1)-F(1) 85.20(7), O(2)-Nb(1)-F(2) 98.24(10), O(3)-Nb(1)-F(1) 86.30(7), O(3)-Nb(1)-F(2) 97.84(10), F(1)-Nb(1)-F(2) 88.76(7), F(1)-Nb(1)-N(1) 84.43(6).

*Note: The successful isolation of the hexafluoroantimonate salt, **3**, of a monocationic monoalkoxide complex, affording molecular weight control consistent with the initiation of one polymer chain per dinuclear initiator molecule, with quantitative incorporation of the ethoxy end group (and thus quantitative reactivity of the ethoxide moiety), was extremely suitable for the current study. Given our subsequent direct preparation of ε-CL adduct **4** (i.e. not via **3**), which is the focus of the current work, and demonstration of the relationship between these species, the authors did not consider it necessary to further explore the role of $AgSbF_6$ stoichiometry in the preparation of **3**. (Please see Page 58, below, for a putative mechanism, by which we propose all ethoxide moieties could enter the catalytic regime irrespective of which Nb-μ_2-F bond of **3** is initially cleaved.)*

Synthesis of Niobium species $[L^{tBu}Nb(OEt)(\varepsilon\text{-CL})]^+[SbF_6]^-$, 4

In a 30 ml vial in the glove box, a sample of complex **2** (0.43 mmol, 0.36 g, 1 equivalent) was dissolved in dry toluene (7.2 ml). To this was added ε-caprolactone (1.75 mmol, 200 µL, 4 equivalents) followed by $AgSbF_6$ (0.43 mmol, 0.15 g, 1 equivalent). The vial was sealed and shaken vigorously for 30 seconds, precipitating AgCl. The mixture was then filtered twice through a 0.2 µm PTFE syringe filter, into a clean 30 ml vial, which was then sealed. The resulting yellow solution was allowed to stand in the glove box for 20 hours, after which a bright yellow crystalline solid had formed. The solution phase was removed using a syringe and hypodermic needle, and the solid washed with hexane (2 x 20 ml), which was also removed with a syringe and hypodermic needle. The solid was then transferred to a Schlenk flask and dried under dynamic vacuum for 1 hour. The solid material contained 1.33 equivalents of toluene, determined by NMR. This could not be removed by heating under dynamic vacuum due to the thermal instability of the complex **4**. Accordingly, this has been accounted for in calculating the yield, and in elemental analysis. Yield: 0.37 g, 68 %. Crystals suitable for diffraction were obtained on reaction of **2**, ε-caprolactone and $AgSbF_6$ in toluene-d_8 following an analogous procedure.

^{1}**H NMR** (500 MHz, $CDCl_3$, 298 K, δ_H, ppm); 7.37 (1H, *s*, *broad*, *Ar*), 7.30 (2H, *s*, *broad*, *Ar*), 7.18 (1H, *s*, *Ar*), 7.13 (2H, *s*, *Ar*), 4.95 (2H, *q*, *J* = 7.0 Hz, OCH_2CH_3), 4.39 (2H, *broad*, Nb-ε-CL OCH_2), 4.23 (<1H, un-coordinated ε-CL OCH_2)*, 4.06 (1H, PCL OCH_2)**, 3.40-4.26 (6H, *broad*, NCH_2), 2.64 (<1H, un-coordinated ε-CL C(O)CH_2)*, 2.31 (1H, PCL C(O)CH_2)**, 2.10 (2H, *broad*, Nb-ε-CL C(O)CH_2), 1.87 (<1H, un-coordinated ε-CL CH_2)*, 1.77 (<1H, un-coordinated ε-CL 2CH_2)*, 1.65 (2H, PCL CH_2)**, 1.61 (3H, *t*, *J* = 7.0 Hz, OCH_2CH_3), 1.54 (2H, *obscured by* CCH_3 *signal*, Nb-ε-CL CH_2), 1.52 (9H, *s*, CCH_3), 1.39 (PCL CH_2)**, 1.35 (20H, *broad*, Nb-ε-CL CH_2 *and* CCH_3), 1.28 (29H, *s*, Nb-ε-CL CH_2 *and* CCH_3). ^{1}**H NMR** (400 MHz, $CDCl_3$, 233 K, δ_H, ppm); 7.27-7.35 (4H, *m*, *broad*, *Ar*), 7.13-7.18 (2H, *broad*, *Ar*), 4.91 (2H, *m*, OCH_2CH_3), 4.44 (4H, *m*, Nb-ε-CL OCH_2 *and* NC*H*H *and* NC*H*H), 4.27 (<1H, un-coordinated ε-CL OCH_2)* , 4.02 (2H, *m*, PCL OCH_2** *and* NC*H*H), 3.58 (1H, *d*, *J* = 16.0 Hz, NC*H*H), 3.39 (1H, *d*, *J* = 13.5 Hz, NC*H*H), 3.27 (1H, *d*, *J* = 13.5 Hz, NC*H*H), 2.66 (<1H, un-coordinated ε-CL C(O)CH_2)*, 2.48 (1H, *broad*, ε-CL CH*H*) 2.32 (1H, PCL C(O)CH_2)**, 1.61-1.95 (4H, *broad*, Nb-ε-CL CH*H*, *and* uncoordinated ε-CL CH_2*, *and* PCL CH_2**), 1.60 (3H, *t*, *J* = 7.0 Hz, OCH_2CH_3), 1.49 (9H, *s*, CCH_3), 1.45 (2H, *broad*, Nb-ε-CL CH_2), 1.35 (9H, *s*, $C(CH_3)_3$), 1.25 (27H, *s*, $C(CH_3)_3$), 1.22 (9H, *s*, $C(CH_3)_3$).*** ^{13}**C{^{1}H} NMR** (101 MHz, $CDCl_3$, 298 K δ_c, ppm); 138.0 (*Ar*), 129.2 (*Ar*H), 128.4 (*Ar*H), 125.5 (*Ar*H), 76.9 (OCH_2CH_3, *obscured by* $CDCl_3$ *signal*), 75.2 (Nb-ε-CL OCH_2), 35.2 (PCL CH_2)**, 34.7 (Nb-ε-CL CH_2), 31.6 ($C(CH_3)_3$), 30.1 ($C(CH_3)_3$), 30.1 ($C(CH_3)_3$), 30.0 ($C(CH_3)_3$), 18.6 (OCH_2CH_3). ^{13}**C{^{1}H} NMR** (101 MHz, $CDCl_3$, 233 K δ_c, ppm); 186.9 (Nb-ε-CL O=*C*), 155.9 (*Ar*O), 145.8 (*Ar*), 136.9 (*Ar*), 124.7 (*Ar*), 124.5 (*Ar*), 76.8 (OCH_2CH_3), 75.0 (Nb-ε-CL OCH_2), 64.4 (PCL CH_2)**, 61.4 (NCH_2), 61.4 (NCH_2), 58.6 (NCH_2), 35.3 ($C(CH_3)_3$), 35.1 ($C(CH_3)_3$), 34.7 ($C(CH_3)_3$), 34.6 ($C(CH_3)_3$), 34.1 (Nb-ε-CL CH_2), 33.9 ($C(CH_3)_3$), 31.5 ($C(CH_3)_3$), 31.4 ($C(CH_3)_3$), 29.7 ($C(CH_3)_3$), 29.4 ($C(CH_3)_3$), 28.3 (PCL CH_2)**, 27.3 (Nb-ε-CL CH_2), 26.8 (uncoordinated ε-CL CH_2)*, 24.6 (PCL CH_2)**, 22.9 (Nb-ε-CL CH_2), 18.6 (OCH_2CH_3). **Elemental (CHN) Analysis** (Calculated, for $C_{53}H_{81}F_6NO_6NbSb$ + 1.33x{C_7H_8}); C: 58.47 %, H: 7.22 %, N: 1.10 %, (Experimental); C: 58.59 %, H: 7.98 %, N: 1.27 %.****

ESI-MS (m/z): 920.5136; calc. for $[C_{53}H_{81}NO_6Nb]^+$ (***4***): 920.5122.

(m/z): 806.4459; calc. for $[C_{47}H_{71}NO_4Nb]^+$ (***4*** *after loss of ε-CL*): 806.4441.

** Residual ε-CL impurity, not removed by washing with apolar solvents (toluene, hexane)*

*** PCL formed in-situ in $CDCl_3$ solution via ROP of residual ε-CL. Absence of visible ethoxy end-group signals indicates only a negligible fraction of the sample of **4** has undergone initiation (intramolecular nucleophilic attack)*

**** Large residual signals corresponding to 1.33 equivalents of lattice toluene have been omitted from reported ^{1}H NMR data*

***** Elemental composition was calculated inclusive of 1.33 equivalents of lattice toluene, determined via integration of the toluene methyl signal of the ^{1}H NMR spectrum of **4** in $CDCl_3$.*

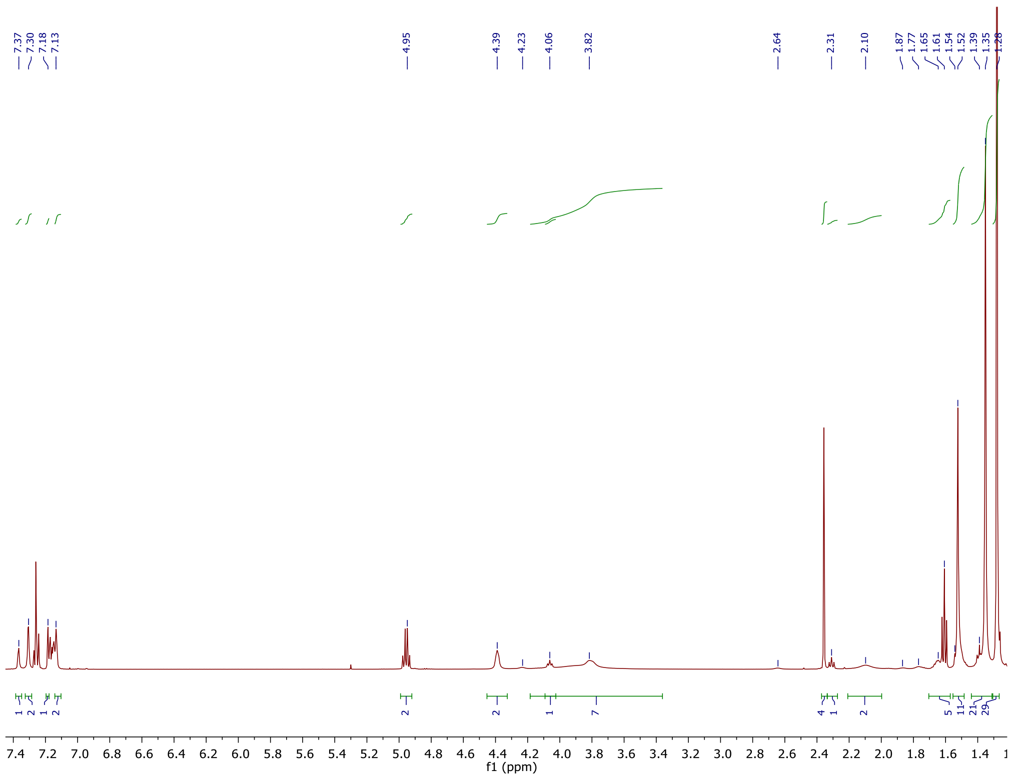

Figure S36. ^{1}H NMR spectrum of **4** in $CDCl_3$ at 298 K

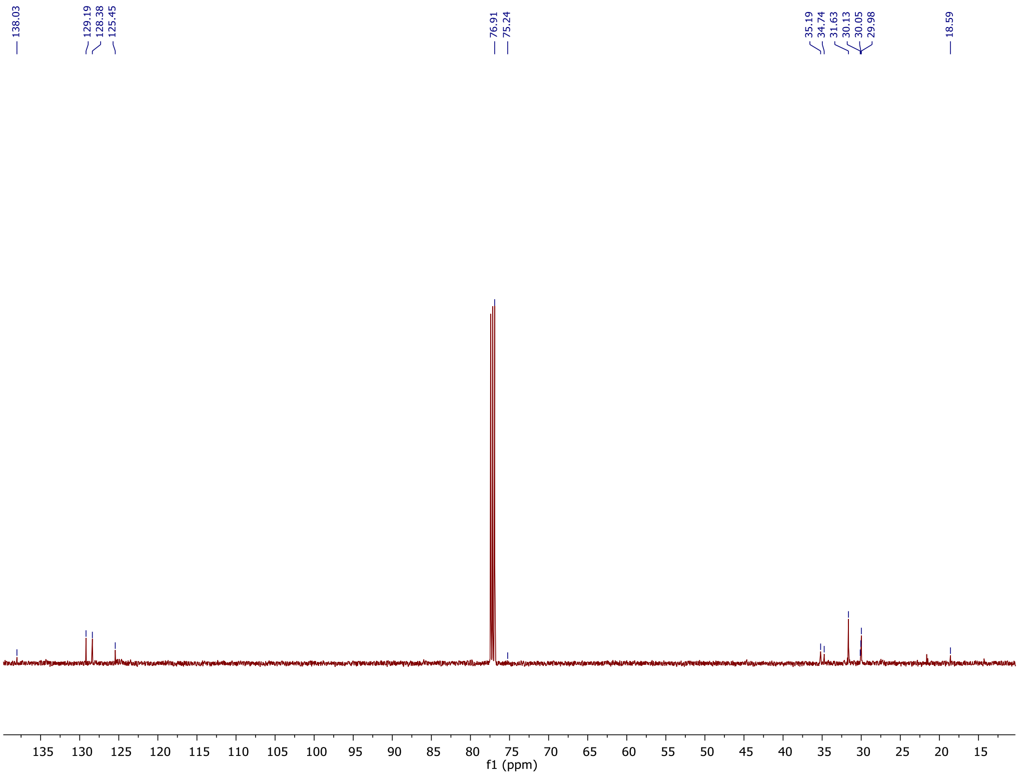

Figure S37. $^{13}C\{^{1}H\}$ NMR spectrum of **4** in $CDCl_3$ at 298 K

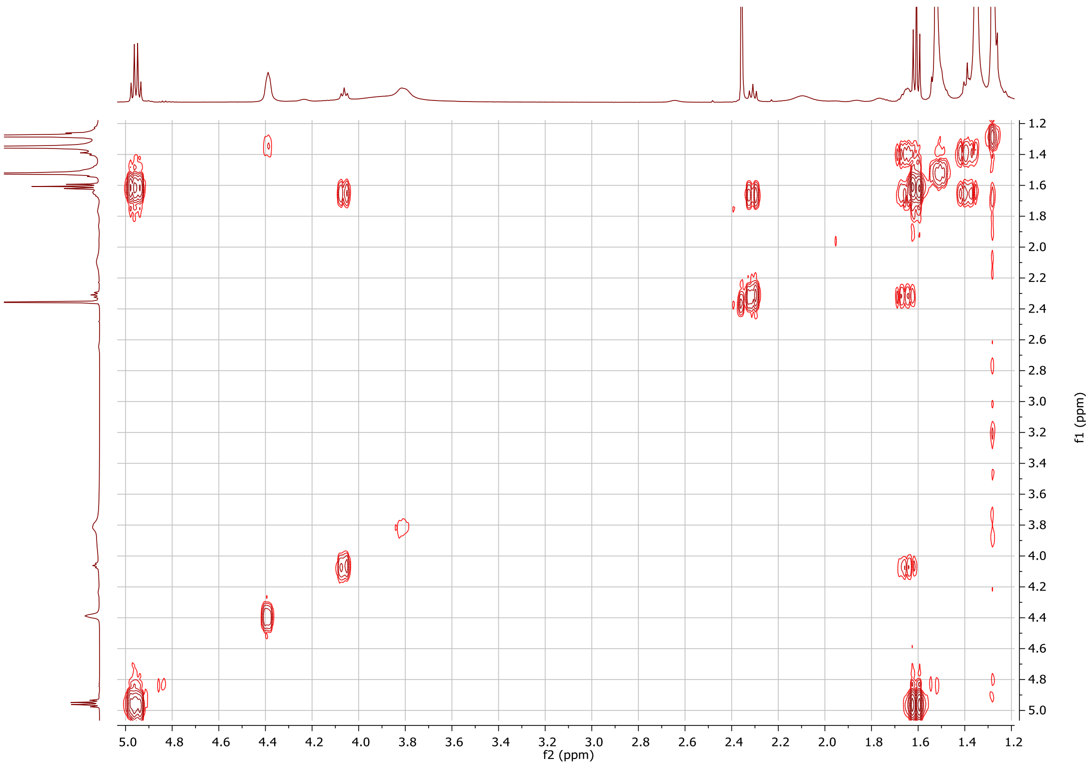

Figure S38. COSY NMR spectrum of **4** in $CDCl_3$ at 298 K

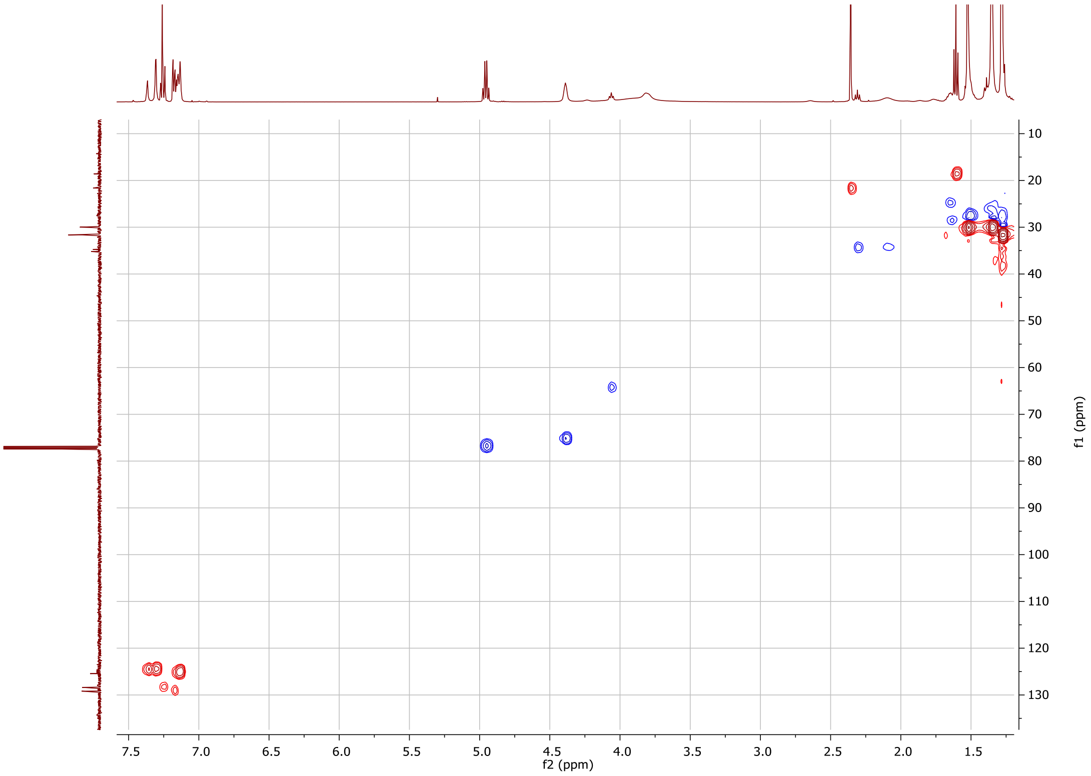

Figure S39. HSQC NMR spectrum of **4** in $CDCl_3$ at 298 K

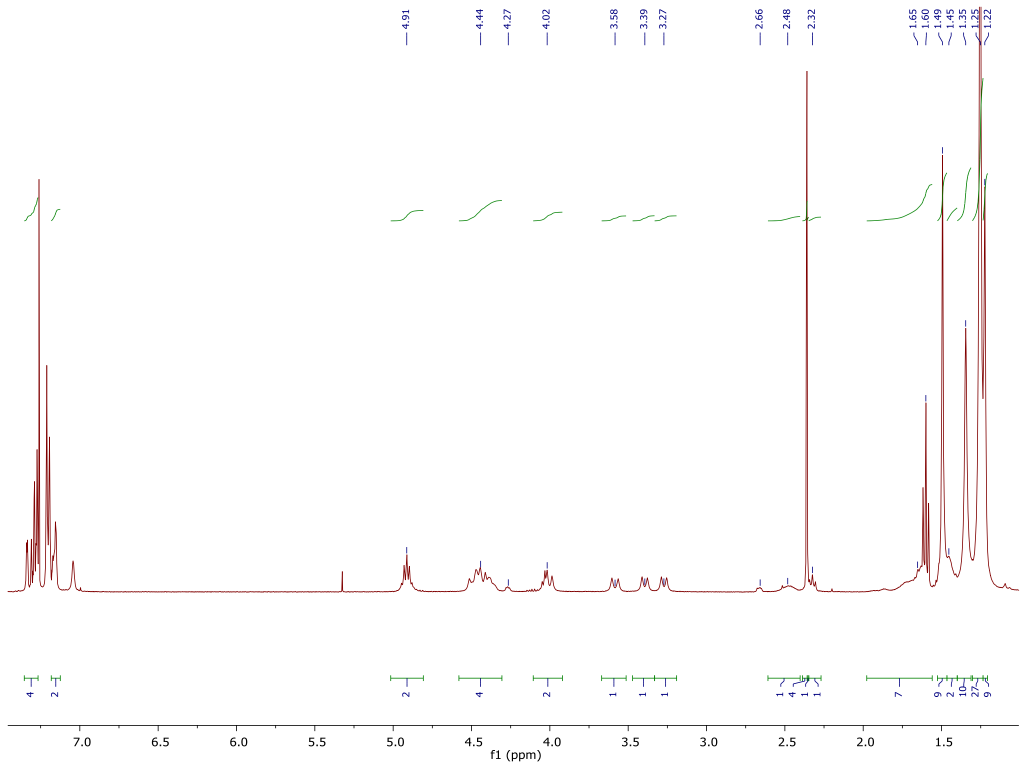

Figure S40. ^{1}H NMR spectrum of **4** in $CDCl_3$ at 233 K

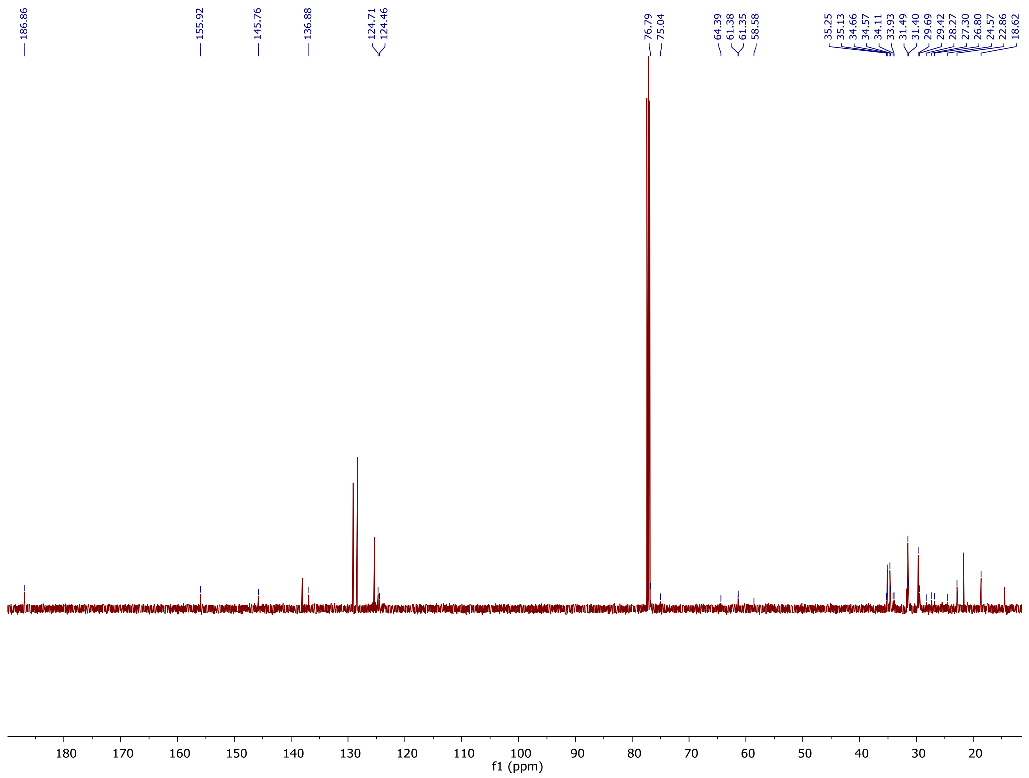

Figure S41. $^{13}C\{^{1}H\}$ NMR spectrum of **4** in $CDCl_3$ at 233 K

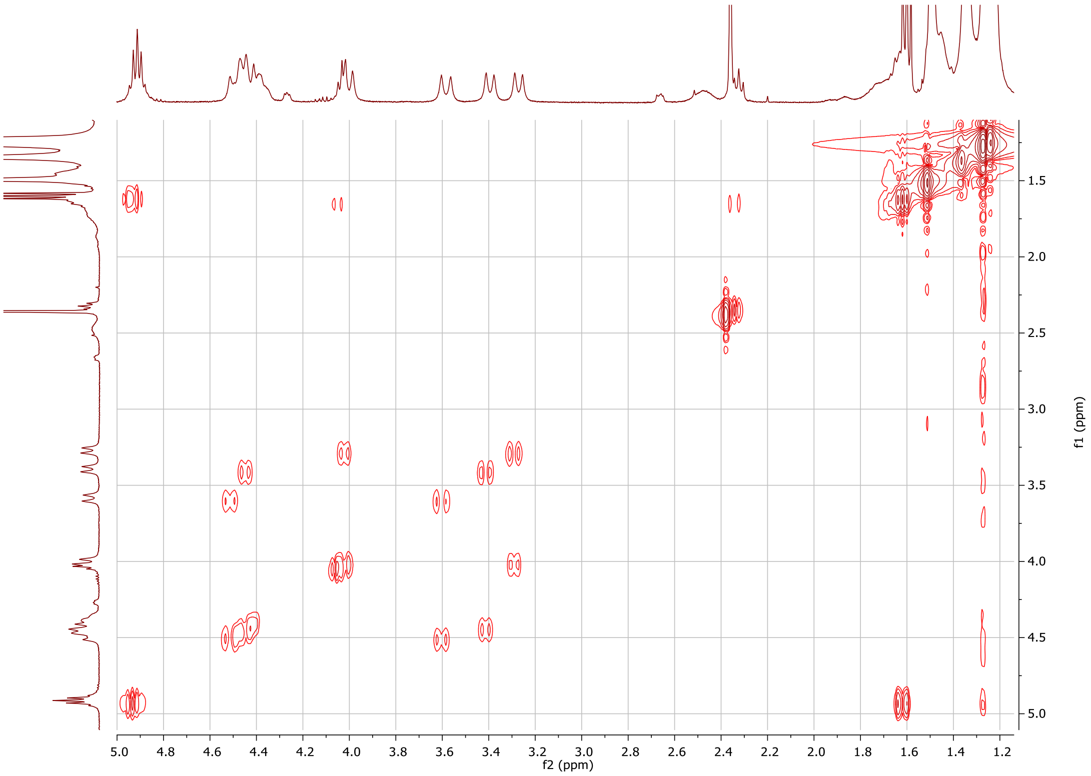

Figure S42. COSY NMR spectrum of **4** in $CDCl_3$ at 233 K

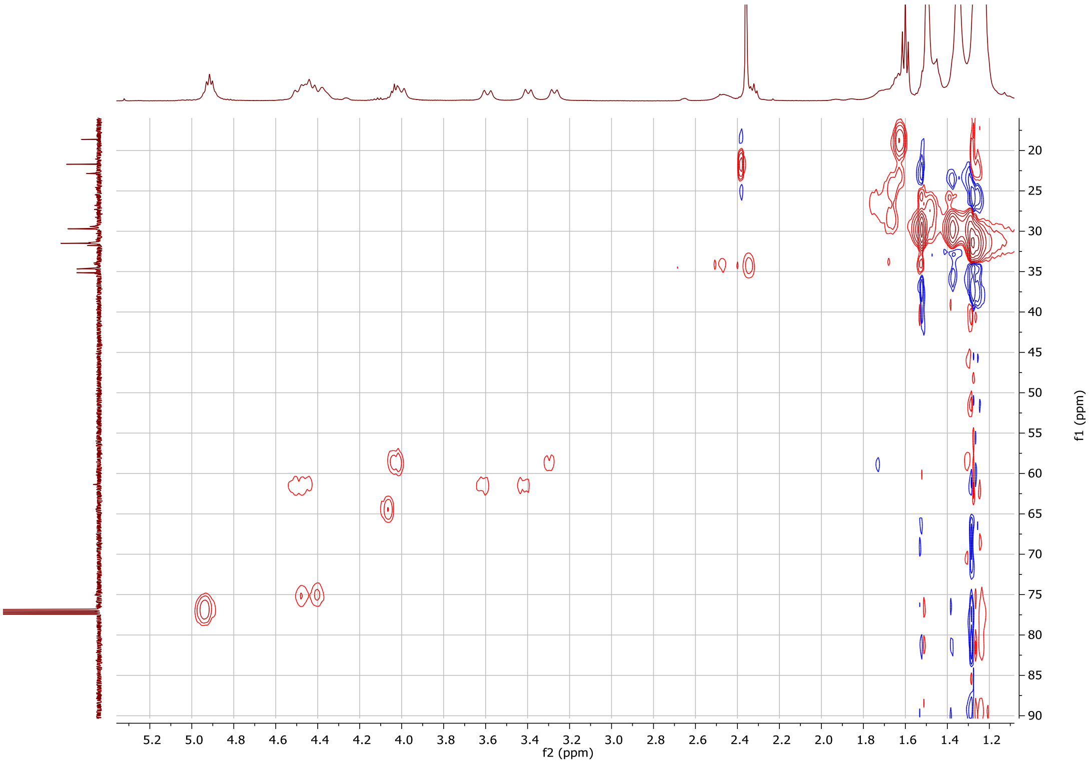

Figure S43. HSQC NMR spectrum of **4** in $CDCl_3$ at 233 K

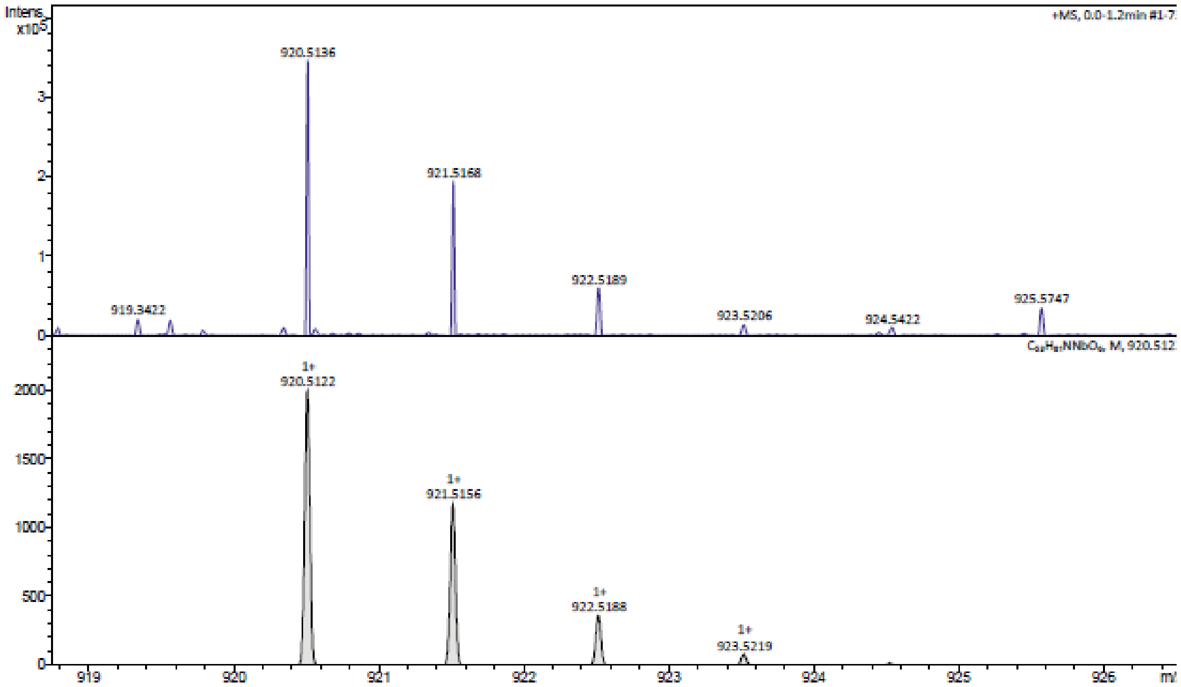

Figure S44. High-resolution mass spectrum detail (top) confirming presence of cation of **4**, [L^{tBu}Nb(OEt)(ε-CL)]$^+$ against simulated spectrum (bottom)

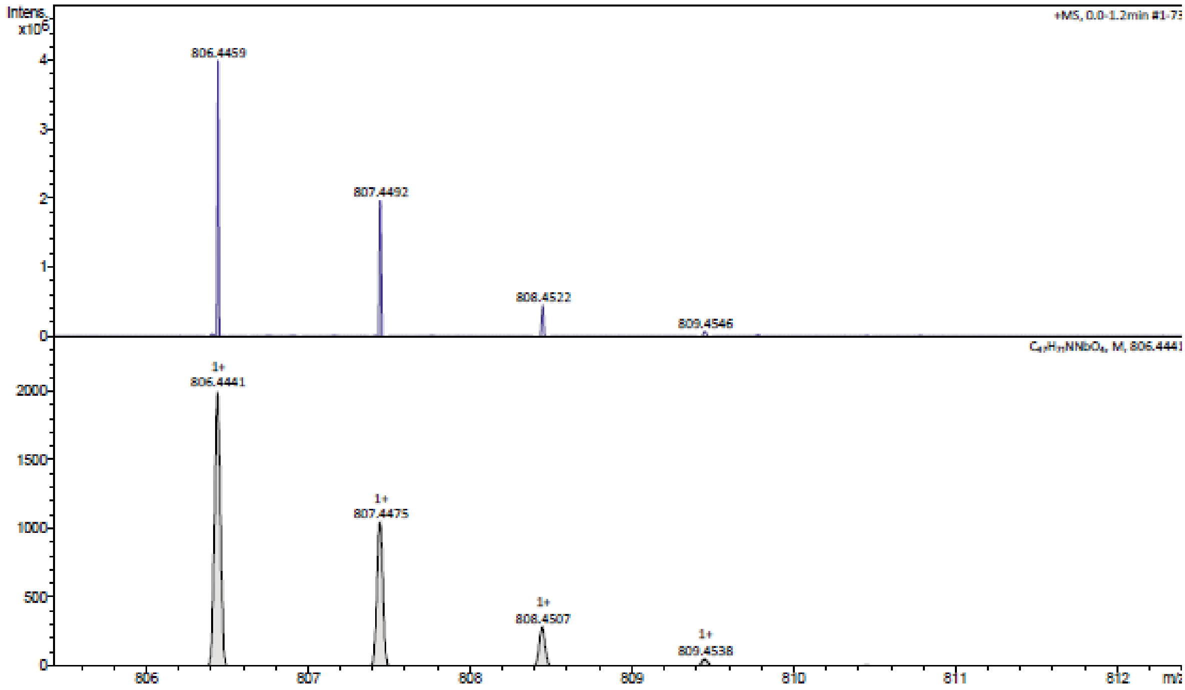

Figure S45. High-resolution mass spectrum detail (top) confirming presence of [L^{tBu}Nb(OEt)]$^+$, product of loss of ε-CL from [L^{tBu}Nb(OEt)(ε-CL)]$^+$, against simulated spectrum (bottom)

Synthesis of Niobium species [$L^{Me}Nb(OEt)_2$], 5

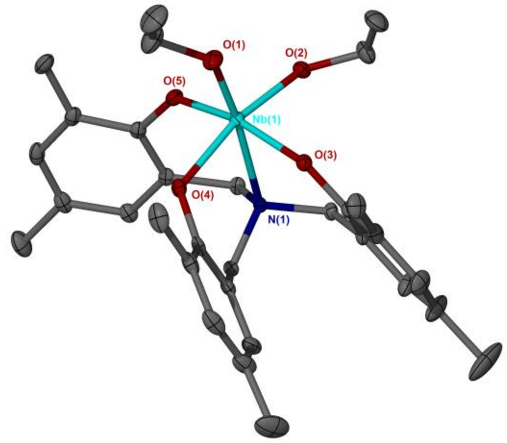

Figure S46. Solid state structure of **5**. Ellipsoids shown at 30% probability level. Hydrogen atoms and lattice solvent have been omitted for clarity. Selected bond lengths (Å) and angles (°): Nb(1)-O(1) 1.8669(15), Nb(1)-O(2) 1.9147(15), Nb(1)-N(1) 2.4195(15), N(1)-Nb(1)-O(1) 173.19(7), O(1)-Nb(1)-O(2) 98.27(7), O(1)-Nb(1)-O(3) 99.27(7), O(1)-Nb(1)-O(4) 94.61(7), O(1)-Nb(1)-O(5) 98.17(7), O(2)-Nb(1)-O(3) 87.76(6), O(2)-Nb(1)-O(5) 92.64(6), O(2)-Nb(1)-N(1) 88.46(6).

To a solution of H_3L^{Me} (21.7 mmol, 9.1 g, 1 equivalent) in THF was added by cannula transfer a solution of $Nb(OEt)_5$ (21.7 mmol, 6.8 g, 1 equivalent) in THF at 25 °C. A yellow colour was immediately observed. After stirring for two hours at ambient temperature, the solvent was removed under dynamic vacuum to yield an oily yellow solid. Washing with hexane (2 x 50 ml) produced a yellow powder which, after cannula filtration, was dried under dynamic vacuum. Yield: 9.1 g, 70 % Crystals suitable for diffraction were obtained by recrystallizing from a mixture of hexane and toluene.

^{1}H NMR (400 MHz, Toluene-*d$_8$*, 298 K, δ_H, ppm); 6.74 (2H, *s*, *Ar*), 6.68 (1H, *s*, *Ar*), 6.55 (2H, *s*, *Ar*), 6.24 (1H, *s*, *Ar*), 4.84 (2H, *q*, *J* = 7.0 Hz, OC*H$_2$*), 3.82 (2H, *d*, *J* = 13.3 Hz, NCH*H*), 3.75 (2H, *q*, *J* = 6.9 Hz, OC*H$_2$*), 3.31 (2H, *s*, NC*H$_2$*), 3.17 (2H, *d*, *J* = 13.3 Hz, NCH*H*), 2.34 (3H, *s*, Ar*C*H$_3$), 2.25 (6H, *s*, Ar*C*H$_3$), 2.20 (6H, *s*, Ar*C*H$_3$), 2.10 (3H, *s*, ArCH$_3$), 1.51 (3H, *t*, *J* = 7.0 Hz, OCH$_2$C*H$_3$*), 0.83 (3H, *t*, *J* = 6.9 Hz, OCH$_2$C*H$_3$*). **^{1}H NMR** (400 MHz, $CDCl_3$, 298 K, δ_H, ppm); 6.80 (2H, *s*, *Ar*), 6.69 (2H, *s*, *Ar*), 6.66 (1H, *s*, *Ar*), 6.26 (1H, *s*, *Ar*), 4.87 (2H, *q*, *J* = 7.0 Hz, OC*H$_2$*CH$_3$), 4.15 (2H, *d*, *J* = 13.0 Hz, NCH*H*), 3.93 (2H, *q*, *J* = 7.0 Hz OC*H$_2$*CH$_3$), 3.46 (2H, *s*, NC*H$_2$*), 3.40 (2H, *d*, *J* = 13.0 Hz, NCH*H*), 2.21 (9H, *s*, ArC*H$_3$*), 2.15 (6H, *s*, ArC*H$_3$*), 2.03 (3H, *s*, ArC*H$_3$*), 1.56 (3H, *t*, *J* = 7.0 Hz, OCH$_2$C*H$_3$*), 0.99 (3H, *t*, *J* = 7.0 Hz, OCH$_2$C*H$_3$*). **^{13}C{^{1}H} NMR** (101 MHz, Toluene-*d$_8$*, 298 K, δ_c, ppm); 156.6 (*Ar*O), 156.4 (*Ar*O), 131.5 (*Ar*), 131.0 (*Ar*), 128.4 (*Ar*), 128.1 (*Ar*), 127.8 (*Ar*), 125.8 (*Ar*), 123.9 (*Ar*), 72.5 (O*C*H$_2$), 68.1 (O*C*H$_2$), 62.9 (N*C*H$_2$), 60.5 (N*C*H$_2$), 20.7 (Ar*C*H$_3$), 20.7 (Ar*C*H$_3$), 18.7 (OCH$_2$*C*H$_3$), 18.6 (OCH$_2$*C*H$_3$), 16.8 (Ar*C*H$_3$), 16.5 (Ar*C*H$_3$). **^{13}C{^{1}H} NMR** (101 MHz, $CDCl_3$, 298 K, δ_c, ppm); 156.1 (*Ar*O), 155.9 (*Ar*O), 131.2 (*Ar*), 130.5 (*Ar*), 128.8 (*Ar*), 128.0 (*Ar*), 127.7 (*Ar*), 127.5 (*Ar*), 125.7 (*Ar*), 125.0 (*Ar*), 124.9 (*Ar*), 123.7 (*Ar*), 72.6 (O*C*H$_2$), 68.5 (O*C*H$_2$), 63.4 (N*C*H$_2$), 60.3 (N*C*H$_2$) , 20.7 (Ar*C*H$_3$), 20.5 (Ar*C*H$_3$), 18.6 (OCH$_2$*C*H$_3$), 18.4 (OCH$_2$*C*H$_3$), 16.5 (Ar*C*H$_3$), 16.2 (Ar*C*H$_3$). **Elemental (CHN) Analysis** (Calculated, for $C_{31}H_{40}NNbO_5$); C: 62.10 %, H: 6.72 %, N: 2.34 %, (Experimental); C: 62.16 %, H: 6.99 %, N: 2.40 %.

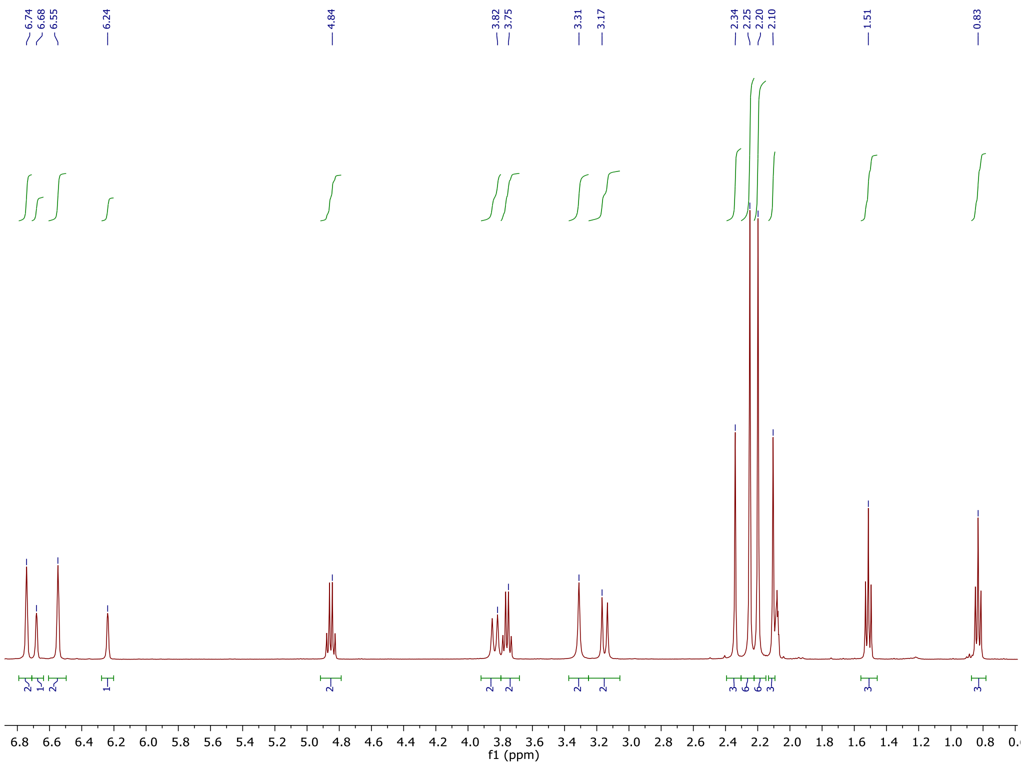

Figure S47. ^{1}H NMR spectrum of **5** in toluene-d_8 at 298 K

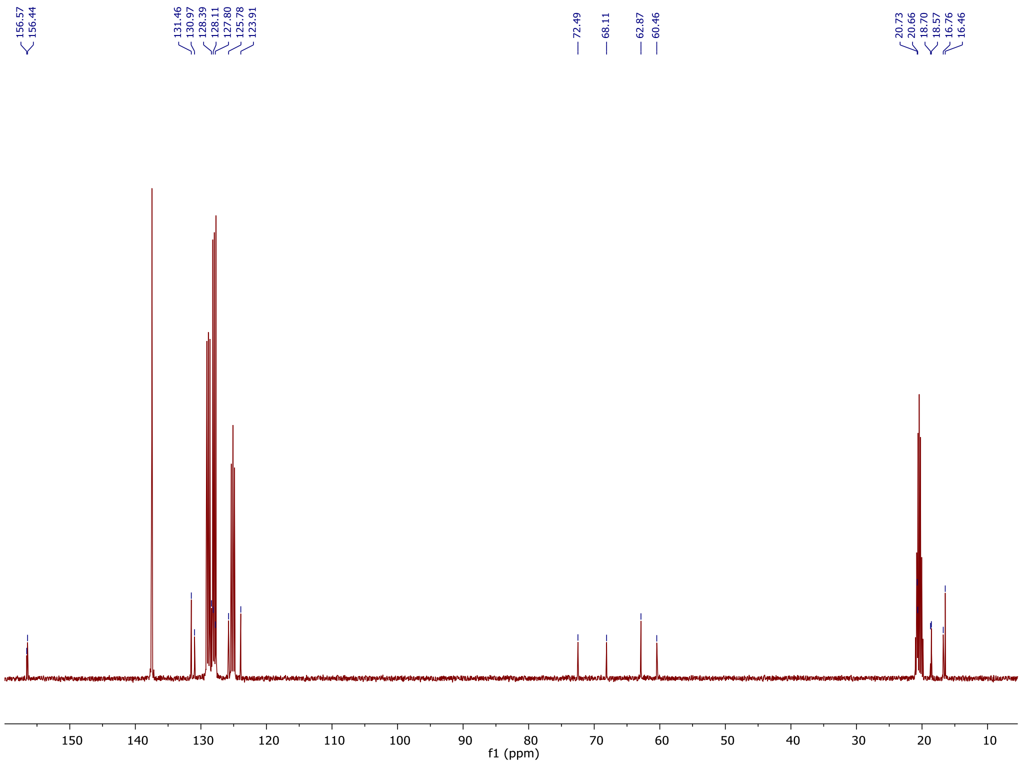

Figure S48. ^{13}C{^{1}H} NMR spectrum of **5** in toluene-d_8 at 298 K

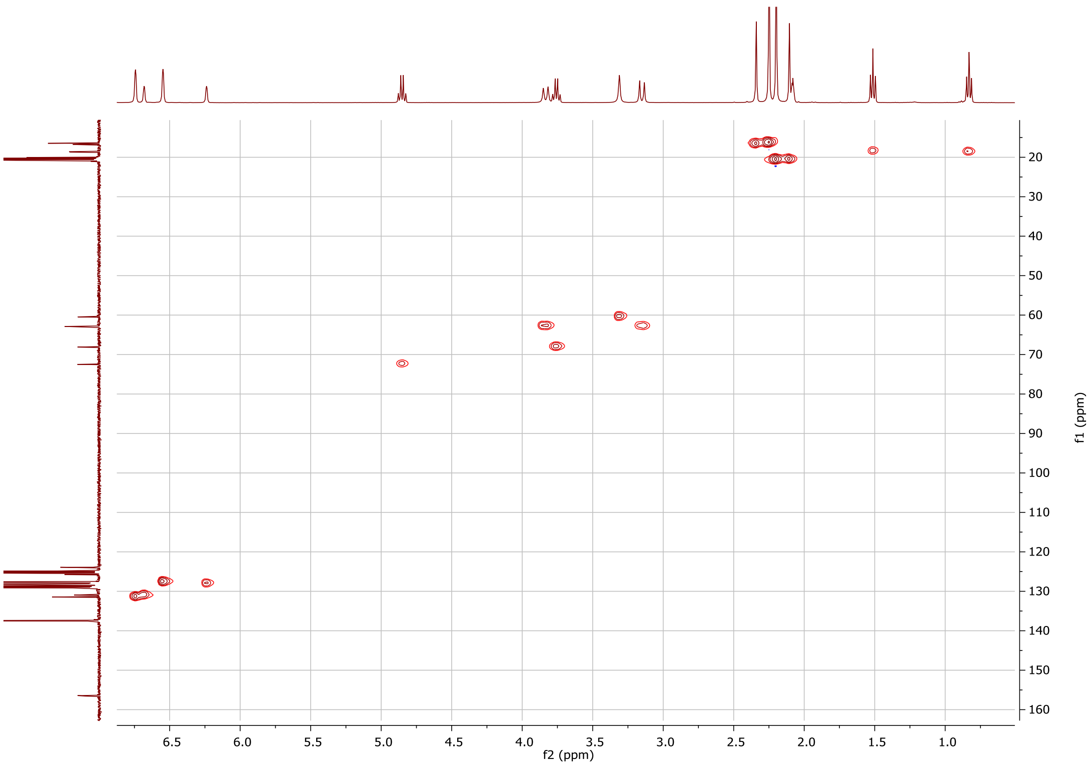

Figure S49. HSQC NMR spectrum of **5** in toluene-d_8 at 298 K

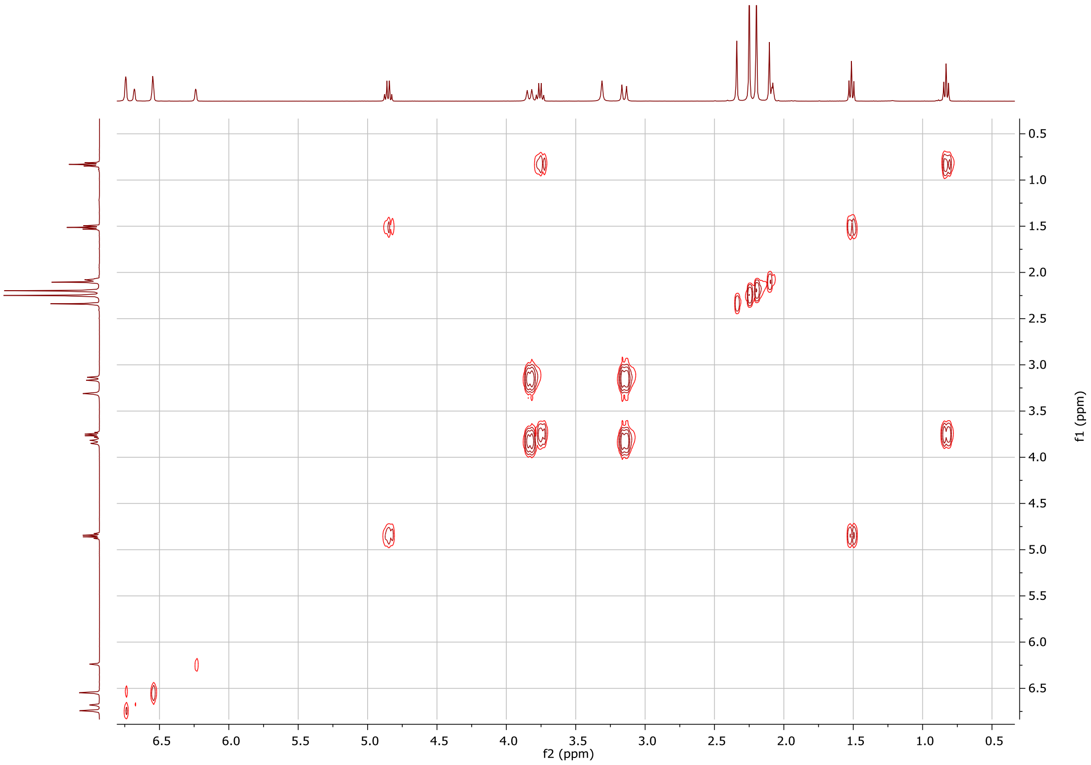

Figure S50. COSY NMR spectrum of **5** in toluene-d_8 at 298 K, allowing correct assignment of alkoxide CH_3 and CH_2 resonances

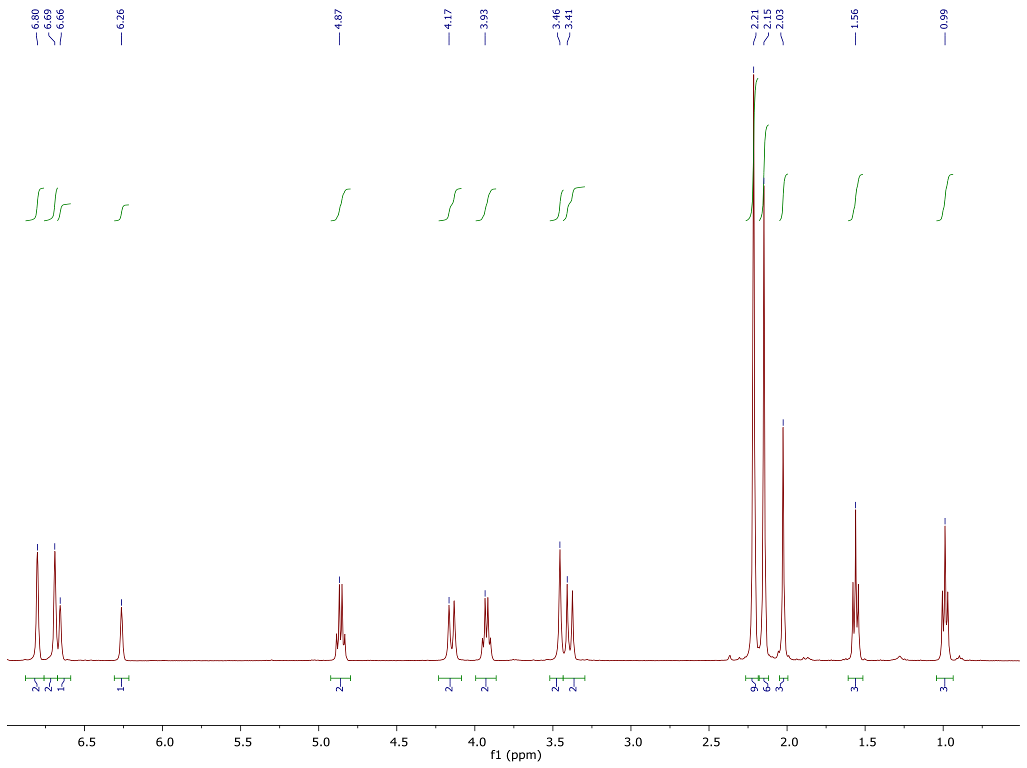

Figure S51. ^{1}H NMR spectrum of **5** in $CDCl_3$ at 298 K

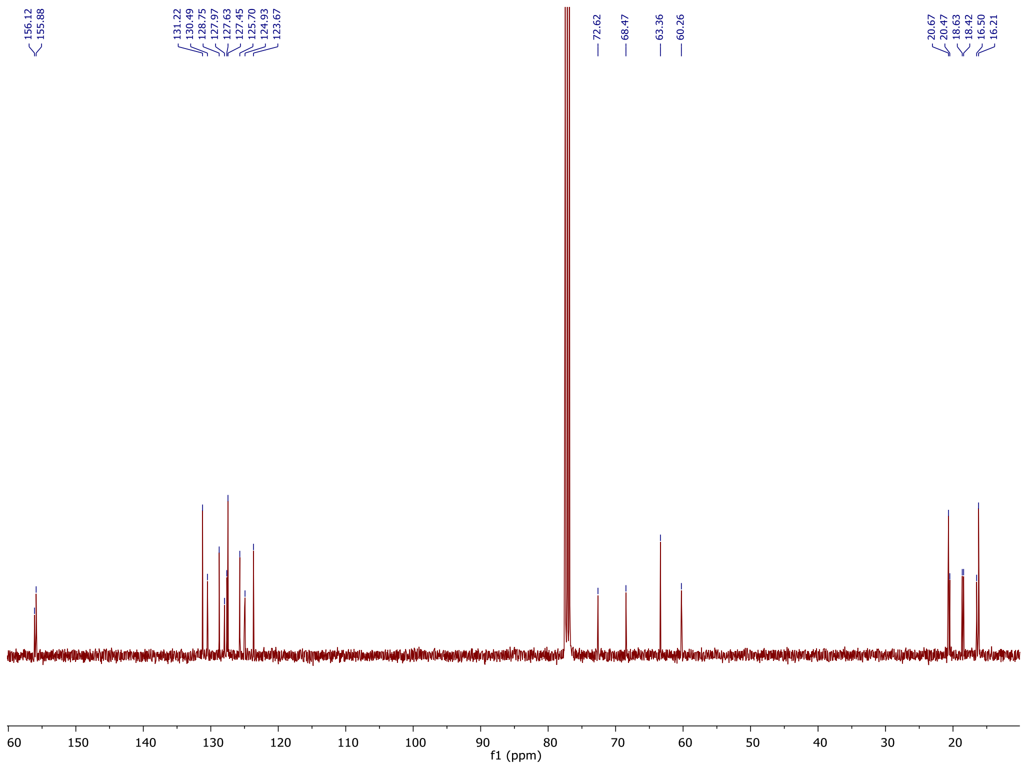

Figure S52. $^{13}C\{^{1}H\}$ NMR spectrum of **5** in $CDCl_3$ at 298 K

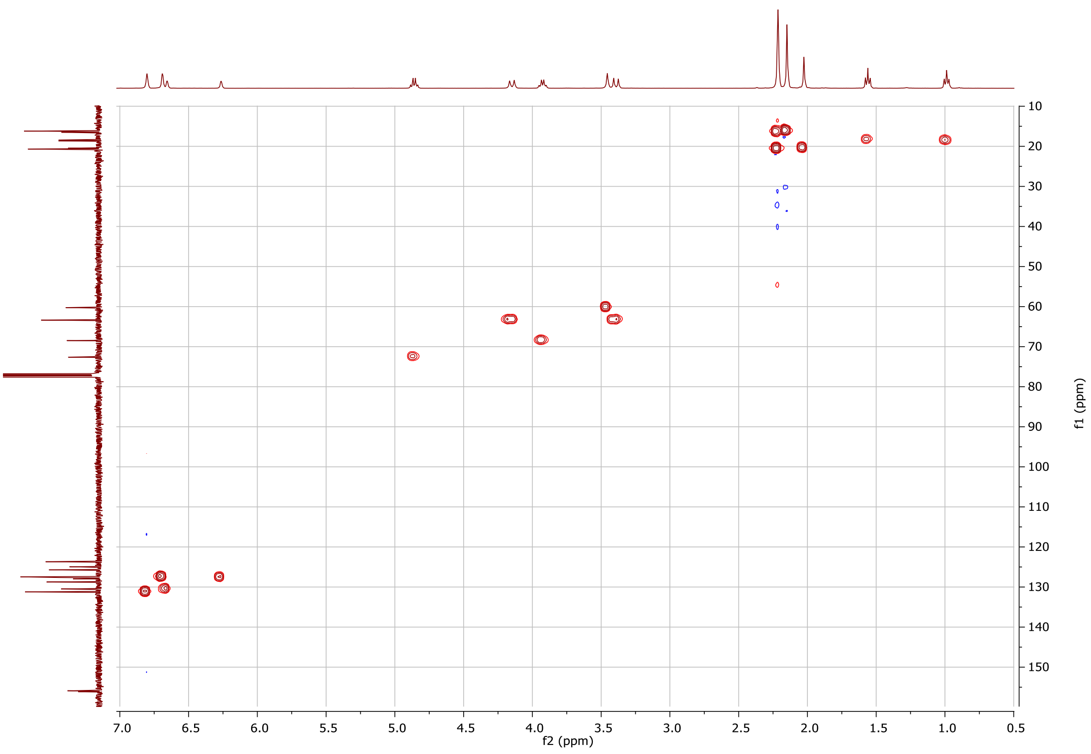

Figure S53. HSQC NMR spectrum of **5** in $CDCl_3$ at 298 K

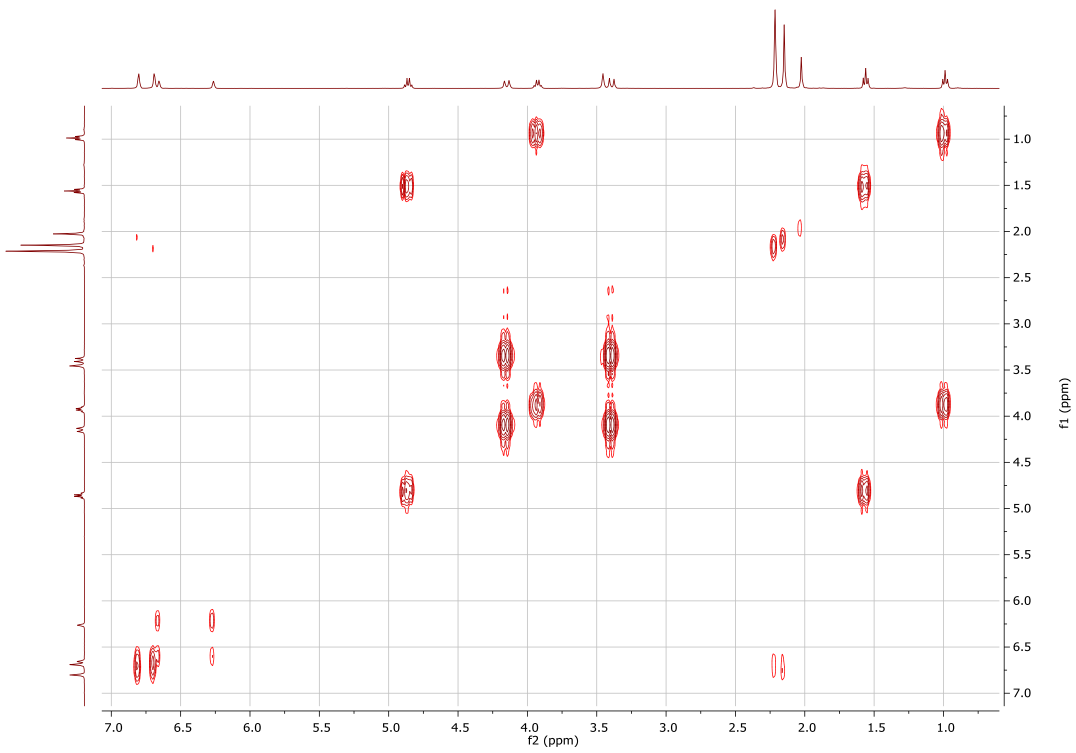

Figure S54. COSY NMR spectrum of **5** in $CDCl_3$ at 298 K, allowing correct assignment of alkoxide CH_3 and CH_2 resonances

Synthesis of Niobium species [L^{Me}Nb(OEt)Cl], 6

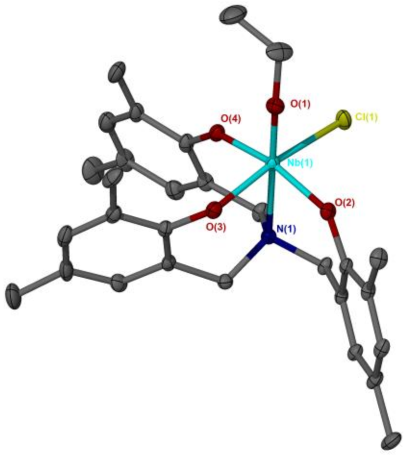

Figure S55. Solid state structure of **6**. Ellipsoids shown at 30% probability level. Hydrogen atoms and lattice solvent have been omitted for clarity. Selected bond lengths (Å) and angles (°): Nb(1)-O(1) 1.838(5), Nb(1)-Cl(1) 2.4211(16), Nb(1)-N(1) 2.418(5), N(1)-Nb(1)-O(1) 178.7(2), O(1)-Nb(1)-O(2) 98.4(2), O(1)-Nb(1)-O(3) 100.2(2), O(1)-Nb(1)-O(4) 99.4(2), O(1)-Nb(1)-Cl(1) 93.04(16), O(2)-Nb(1)-Cl(1) 90.51(13), O(4)-Nb(1)-Cl(1) 88.23(13), N(1)-Nb(1)-Cl(1) 86.21(12).

To a solution of **5** (15.4 mmol, 9.2 g, 1 equivalent) in dichloromethane was added excess chlorotrimethylsilane (39 mmol, 5.0 ml, 2.5 equivalents). Stirring for 24 hours yielded a dark red-brown solution. Solvent, unreacted chlorotrimethylsilane, and by-product ethoxytrimethylsilane were all removed under dynamic vacuum to yield a yellow-brown solid. The solid was washed with hexane (2 x 50 ml) and after cannula filtration, was dried under dynamic vacuum. Yield: 7.0 g, 77 % Crystals suitable for diffraction were obtained by recrystallizing from a mixture of THF and toluene.

^{1}H NMR (400 MHz, Toluene-d_8, 298 K δ_H, ppm); 6.66 (2H, *s*, *Ar*) 6.53 (1H, *s*, *Ar*), 6.49 (2H, *s*, *Ar*), 6.00 (1H, *s*, *Ar*), 4.77 (2H, *q*, *J* = 7.0 Hz, O*CH₂*), 4.57 (2H, *d*, *J* = 13.5 Hz, NC*H*H), 3.15 (2H, *s*, N*CH₂*), 2.94 (2H, *d*, *J* = 13.5 Hz, NC*H*H), 2.23 (3H, *s*, ArC*H₃*), 2.17 (12H, *s*, ArC*H₃*), 1.95 (3H, *s*, ArC*H₃*), 1.46 (3H, *t*, *J* = 7.0 Hz, OCH₂C*H₃*). **^{1}H NMR** (400 MHz, $CDCl_3$, 298 K δ_H, ppm); 6.82 (2H, *d*, *J* = 2.0 Hz, *Ar*), 6.73 (2H, *d*, *J* = 2.0 Hz, *Ar*), 6.68 (1H, *d*, *J* = 2.0 Hz, *Ar*), 6.25 (1H, *d*, *J* = 2.0 Hz, *Ar*), 4.97 (2H, *q*, *J* = 7.0 Hz, O*CH₂*CH₃), 4.67 (2H, *d*, *J* = 13.5 Hz, NCH*H*), 3.47 (2H, *s*, N*CH₂*), 3.38 (2H, *d*, *J* = 13.5 Hz, NCH*H*), 2.23 (6H, *s*, ArC*H₃*), 2.21 (3H, *s*, ArC*H₃*), 2.16 (6H, *s*, ArC*H₃*), 2.00 (3H, *s*, ArC*H₃*), 1.65 (3H, *t*, *J* = 7.0 Hz, OCH₂C*H₃*). **^{13}C{^{1}H} NMR** (101 MHz, Toluene-d_8, δ_c, ppm); 156.35 (*Ar*O), 155.64 (*Ar*O), 131.55 (*Ar*H), 130.82 (*Ar*H), 130.42 (*Ar*), 129.43 (*Ar*), 128.02 (*Ar*H), 127.87 (*Ar*H), 125.71 (*Ar*), 125.54 (*Ar*), 124.31 (*Ar*), 76.05 (O*C*H₂), 63.99 (N*C*H₂), 59.85 (N*C*H₂), 20.73 (Ar*C*H₃), 17.60 (OCH₂*C*H₃), 16.29 (Ar*C*H₃), 16.24 (Ar*C*H₃). **^{13}C{^{1}H} NMR** (101 MHz, $CDCl_3$, δ_c, ppm); 155.8 (*Ar*O), 155.1 (*Ar*O), 131.4 (*Ar*), 130.9 (*Ar*), 130.7 (*Ar*), 129.9 (*Ar*), 127.6 (*Ar*), 127.6 (*Ar*), 125.6 (*Ar*), 125.1 (*Ar*), 124.9 (*Ar*), 124.2 (*Ar*), 76.1 (O*C*H₂), 64.0 (N*C*H₂), 60.1 (N*C*H₂), 20.7 (Ar*C*H₃), 20.4 (Ar*C*H₃), 17.7 (OCH₂*C*H₃), 16.1 (Ar*C*H₃), 16.1 (Ar*C*H₃). **Elemental (CHN) Analysis** (Calculated, for $C_{29}H_{35}NNbO_4Cl$); C: 59.04 %, H: 5.98 %, N: 2.37 %, (Experimental); C: 58.44 %, H: 6.34 %, N: 2.33 %.

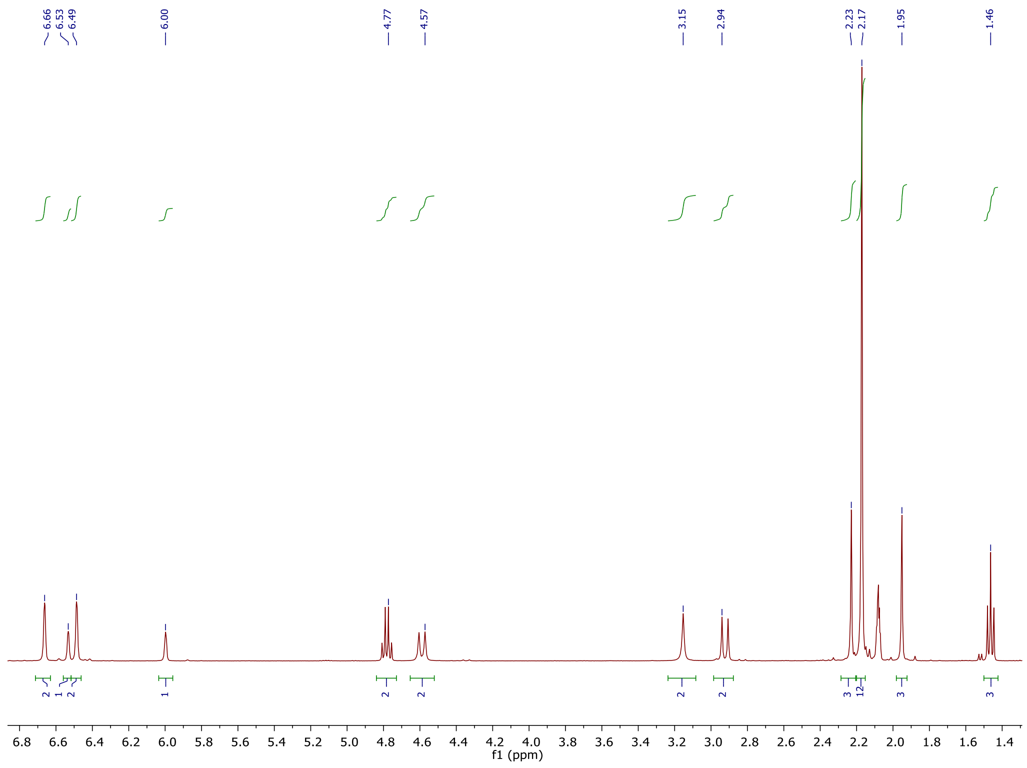

Figure S56. ^{1}H NMR spectrum of **6** in toluene-d_8 at 298 K

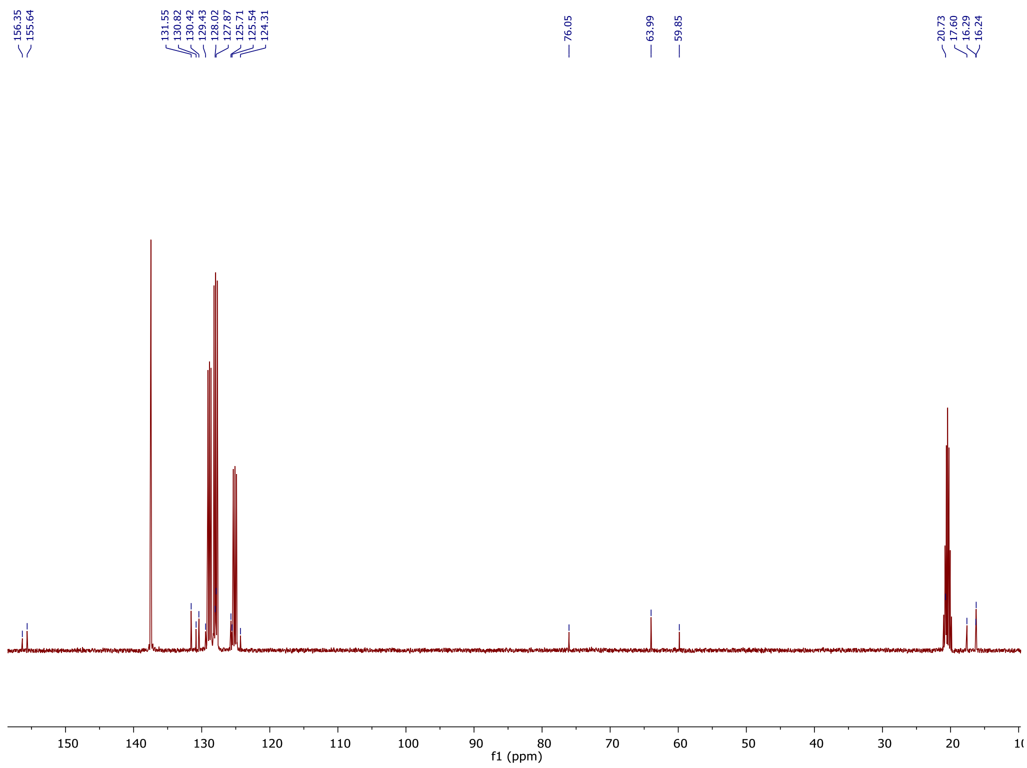

Figure S57. $^{13}C\{^{1}H\}$ NMR spectrum of **6** in toluene-d_8 at 298 K

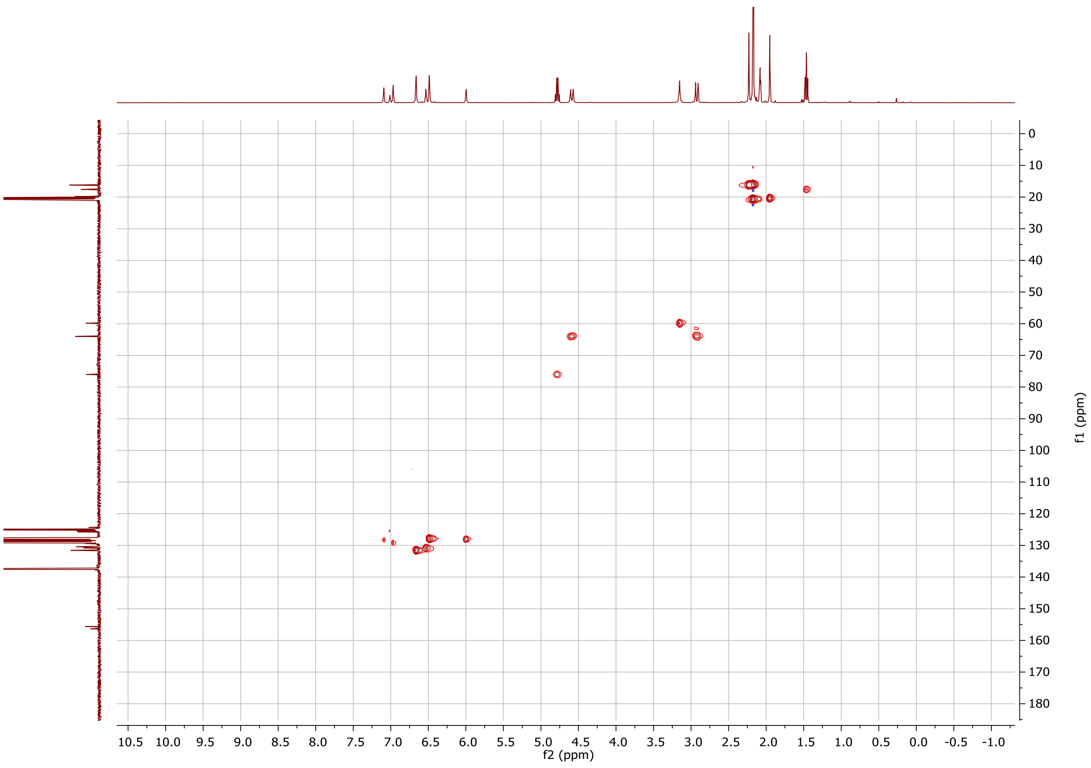

Figure S58. HSQC NMR spectrum of **6** in toluene-d_8 at 298 K

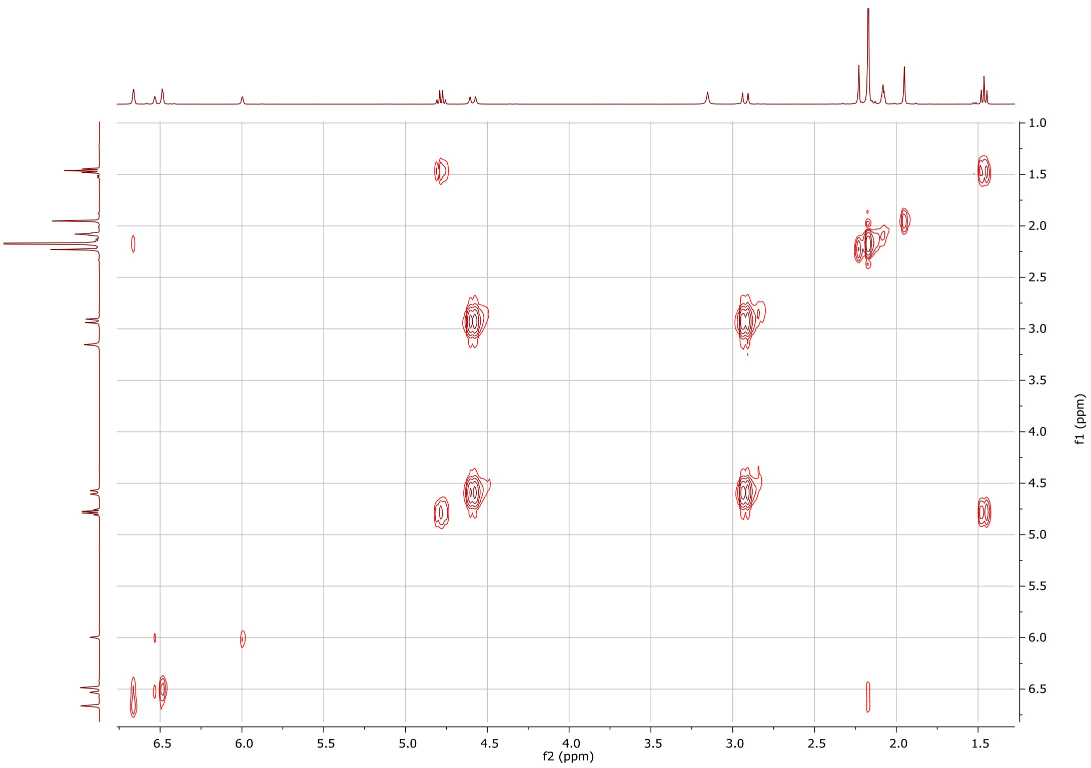

Figure S59. COSY NMR spectrum of **6** in toluene-d_8 at 298 K

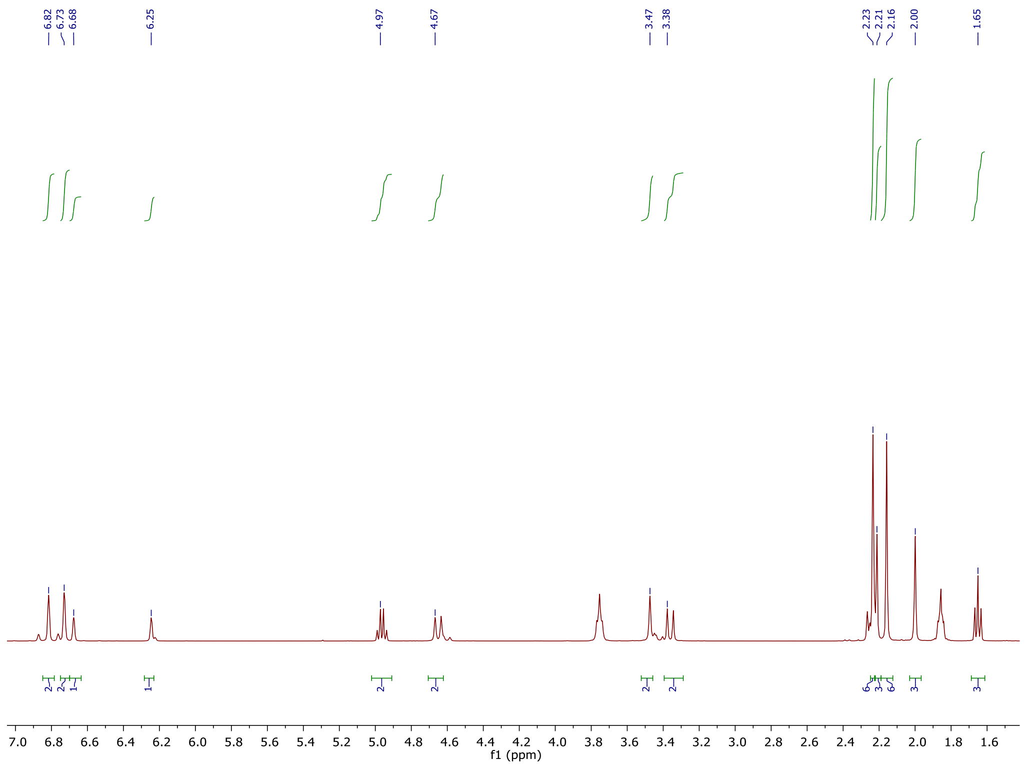

Figure S60. ^{1}H NMR spectrum of **6** in $CDCl_3$ at 298 K

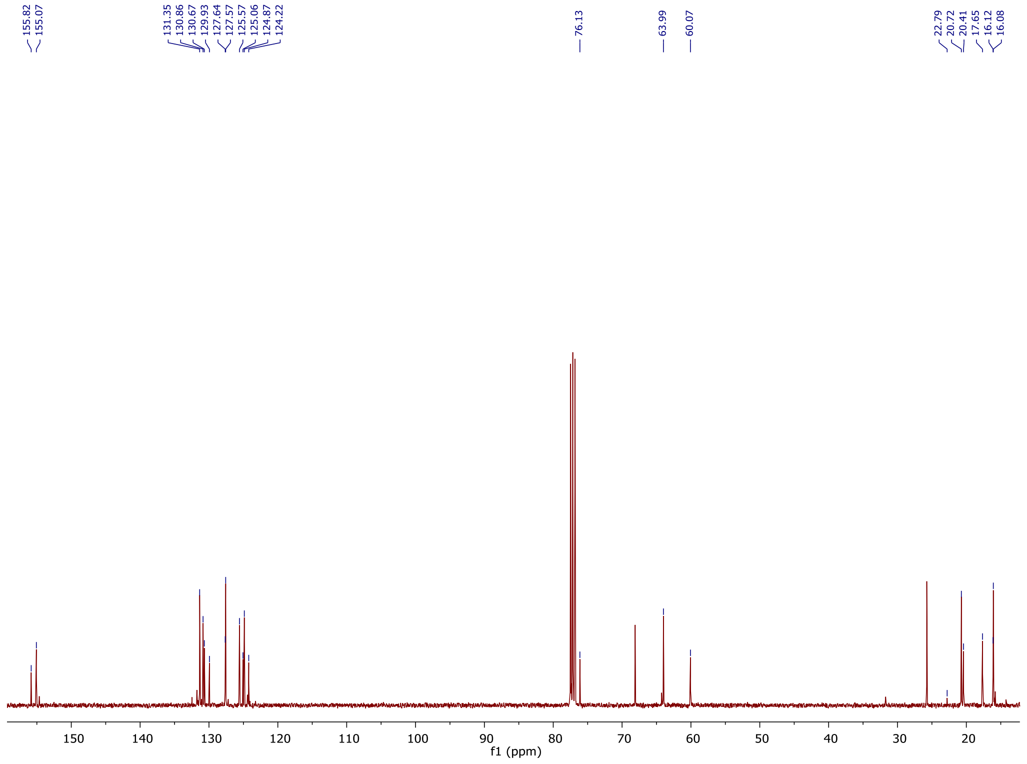

Figure S61. $^{13}C\{^{1}H\}$ NMR spectrum of **6** in $CDCl_3$ at 298 K

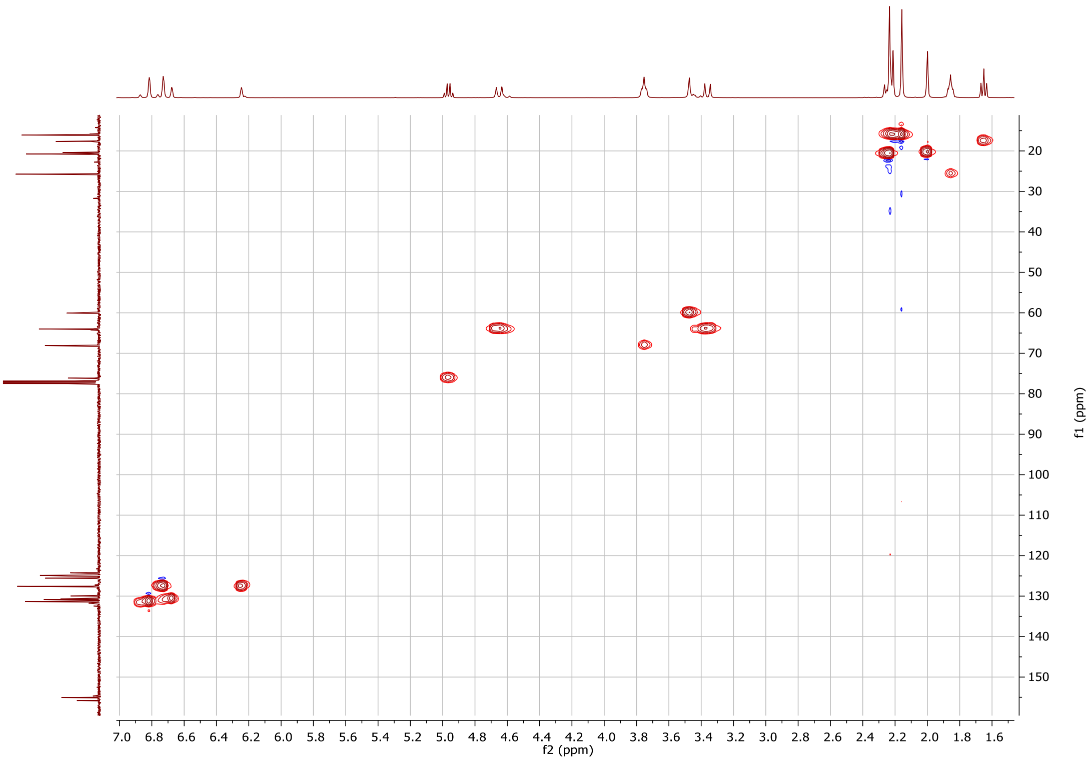

Figure S62. HSQC NMR spectrum of **6** in $CDCl_3$ at 298 K

Synthesis of Niobium species $[\{L^{Me}Nb(OEt)\}_2\text{-}\mu_2F]^+[SbF_6]^-$, 7

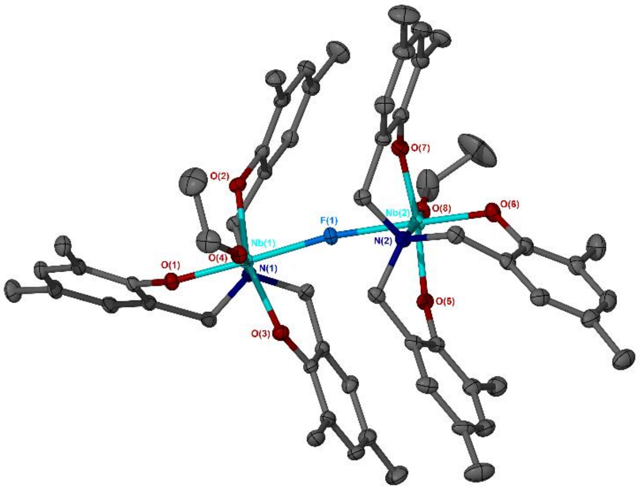

Figure S63. The crystal structure of the bimetallic, monocationic fragment of Nb(V) species **7**. Ellipsoids shown at 30% probability level. Hydrogen atoms, lattice solvent, and SbF_6^- anion have been omitted for clarity. Selected bond lengths (Å) and angles (°): Nb(1)-N(1) 2.368(4), Nb(1)-O(4) 1.838(3), Nb(1)-F(1) 2.112(2), Nb(2)-F(1) 2.110(2), Nb(2)-N(2) 2.379(4), Nb(2)-O(8) 1.841(3), Nb(1)-F(1)-Nb(2) 171.70(14), N(1)-Nb(1)-O(4) 174.86(13), N(2)-Nb(2)-O(8) 174.71(14), O(1)-Nb(1)-O(4) 94.05(14), O(2)-Nb(1)-O(3) 153.17(13), O(2)-Nb(1)-O(4) 100.47(14), O(2)-Nb(1)-F(1) 80.64(12), O(3)-Nb(1)-O(4) 100.24(14), O(3)-Nb(1)-F(1) 81.23(12), O(4)-Nb(1)-F(1) 93.55(12), O(5)-Nb(2)-O(7) 160.92(13), O(5)-Nb(2)-O(8) 99.53(15), O(5)-Nb(2)-F(1) 86.98(12), O(6)-Nb(2)-O(8) 101.70(14), O(7)-Nb(2)-O(8) 98.61(15), O(7)-Nb(2)-F(1) 86.68(12), O(8)-Nb(2)-F(1) 91.42(12), N(1)-Nb(1)-F(1) 91.39(11), N(2)-Nb(2)-F(1) 83.30(11).

In a 30 ml vial in the glove box, a sample of complex **6** (1.70 mmol, 1.0 g, 1 equivalent) was dissolved in dry toluene (4 ml). To this was added $AgSbF_6$ (2.53 mmol, 0.87 g, 1.5 equivalents). The vial was sealed and shaken vigorously for 30 seconds, precipitating AgCl. The mixture was then filtered twice through a 0.2 µm PTFE syringe filter, into a clean 30 ml vial, which was then sealed. The resulting red solution was allowed to stand in the glove box for 20 hours, after which a red solid had precipitated. The solution phase was removed using a syringe and hypodermic needle, and the solid washed with hexane (2 x 10 ml), which was also removed with a syringe and hypodermic needle. The solid was then transferred to a Schlenk flask and dried under dynamic vacuum for 1 hour, yielding a brown powder. Yield: 0.40 g, 35 %. Crystals suitable for diffraction were obtained on reaction of **6** and $AgSbF_6$ in toluene following an analogous procedure, however, the concentration of the reaction mixture was reduced by a factor of 5.

*Unlike **3**, the Nb-F-Nb moiety of **7** is not linear, with a bond angle of 171.70(14)°. Furthermore, whilst the amine tris(phenolate) ligand at one Nb centre of **7** retains the C_1 symmetry of complexes **5** and **6**, the other is C_3 symmetric. A bulk sample of **7** was not isolated in high purity; elemental analysis results were consistently unsatisfactory. We attribute this to decomposition of the crystalline material, in the absence of the kinetic stabilization afforded to complex **3** by the bulkier tert-butyl-substituted ancillary ligand. The ^1H NMR spectrum of **7** at 213 K exhibited highly fluxional character, attributed to rapid exchange of the ligands between C_1 and pseudo-C_3 symmetric conformations, and inversion of helical chirality. The equilibrium presence of monometallic species in solution may also occur. On addition of excess ε-CL, however, the spectrum became well-resolved, suggesting analogous behaviour to **3**; reversible cleavage of the bimetallic cation, to form a cationic adduct with the monomer, and neutral species $[L^{Me}Nb(OEt)F]$. Attempts to isolate the proposed ε-CL adduct, $[L^{Me}Nb(OEt)(\varepsilon\text{-}CL)]^+[SbF_6]^-$, have*

*been without success. Like **3** and **4**, solvation of **7** in THF led to polymerization of the solvent at ambient temperature.*

^{1}H NMR (500 MHz, $CDCl_3$, 213 K, δ_H, ppm); 6.20-7.80 (*m, broad, Ar)*, 5.00 (*broad*, OCH_2CH_3), 4.83 (*broad*, NC*H*H), 4.41 (*broad*, NC*H*H), 4.05 (*broad*, NC*H*H), 3.44 (*broad*, NC*H*H), 3.29 (*broad*, NC*H*H), 3.16 (*broad*, NC*H*H), 1.20-2.75 (*broad*, *m*, $ArCH_3$), 1.72 (*broad*, OCH_2CH_3).* **^{13}C{^{1}H} NMR** (101 MHz, $CDCl_3$, 213 K δ_c, ppm); Acquisition of ^{13}C{^{1}H} NMR data was precluded by the insolubility, and fluxional nature of **7** even at low temperature (213 K). **Elemental (CHN) Analysis** (Calculated, for $C_{58}H_{70}F_7N_2Nb_2O_8Sb$); C: 51.08 %, H: 5.17 %, N: 2.05 %, (Experimental); C: 46.04 %, H: 5.17 %, N: 1.89 %.**

** ^{1}H NMR spectrum could not be fully assigned, or signals integrated due to highly fluxional ^{1}H signals. Chemical shift values were determined from the corresponding COSY spectrum. Addition of ε-CL to **7** yielded a more well-resolved ^{1}H NMR spectrum at 213 K, presumably of the species [L^{Me}Nb(OEt)(ε-CL)]$^+$[SbF_6]$^-$* and [L^{Me}Nb(OEt)F]. *The fluxionality of ^{1}H environments may be due to the lability of the two Nb-μ_2F bonds permitting dissociation of **7**, into cationic and neutral fragments, [L^{Me}Nb(OEt)]$^+$, and [L^{Me}Nb(OEt)F].*

*** Elemental Analysis indicated that the sample **7** was impure, despite extensive efforts to prevent contamination or degradation. We suggest that such impurities are inorganic in nature, and arise from the inherent instability of **7**.*

7 was not detected with high-resolution mass spectrometry. Mass spectrometry samples were not prepared under air- or moisture-free conditions; we suggest that **7** was too unstable under those conditions to be detected. However, the presence of one alkoxide moiety *per* Nb centre (the dinuclear species bearing two alkoxide groups), as determined *via* single crystal x-ray diffraction, is supported by the ratios of EtO- end group and polymer backbone protons in the product of the ROP of ε-CL initiated by **7** (see "End-group analysis", below).

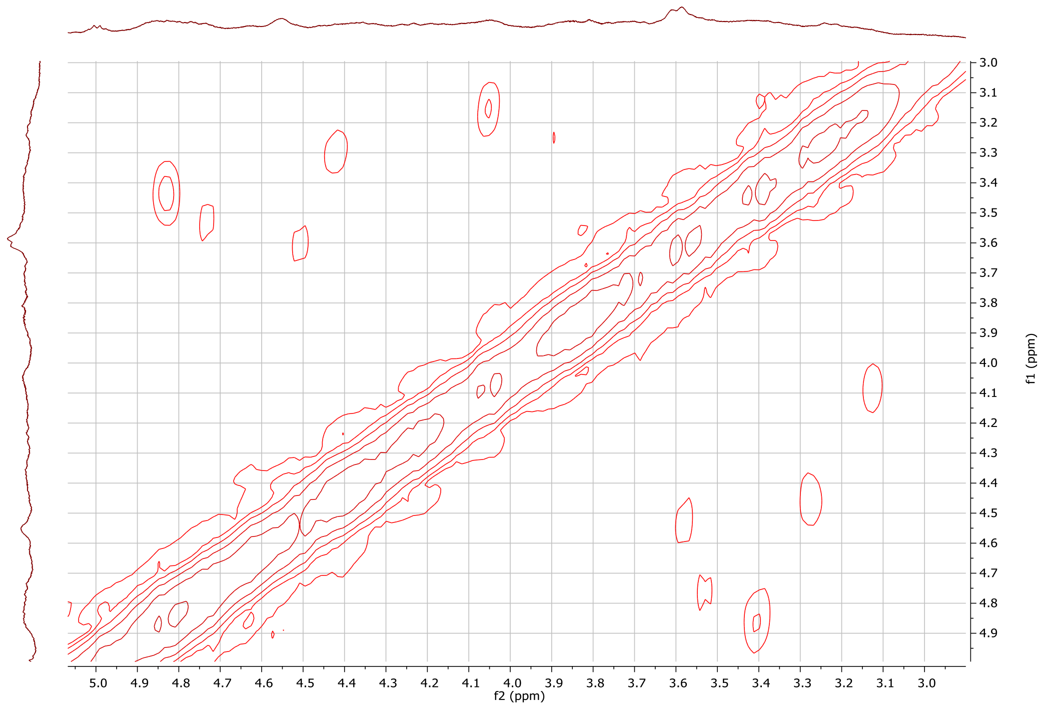

Figure S64. Methylene region of the COSY NMR spectrum of **7** in $CDCl_3$ at 213 K, from which NC*H*H ^1H shifts have been assigned

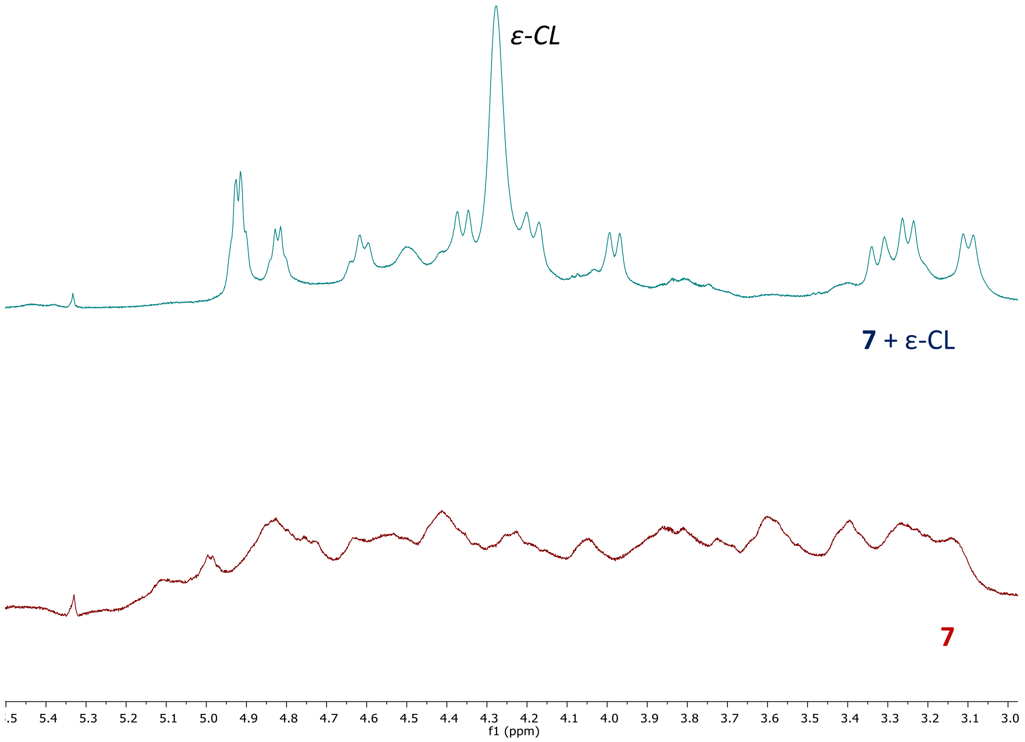

Figure S65. Methylene region of the ^1H NMR spectrum of **7** in $CDCl_3$ at 213 K (bottom) and of the same species following addition of excess ε-CL (top), showing well-resolved signals corresponding to proposed ε-CL adduct **8**. The broad singlet at δ_H= 4.25 ppm corresponds to excess free ε-CL

Stoichiometric reactions

Addition of ε-caprolactone to Nb complex 3

On addition of excess ε-CL to pre-catalyst **3** at ambient temperature, the ^{1}H NMR spectrum acquired at -40 °C suggested cleavage of the dinuclear species occurred at one of the Nb-F bonds, with coordination of ε-CL to the cationic Nb centre yielding ε-CL adduct **4** and, presumably, neutral complex $[L^{tBu}NbF_2]$. When ε-CL was added to the NMR sample of **3** in $CDCl_3$, the insoluble, red, crystalline solid complex was rapidly solubilised, yielding a yellow solution.

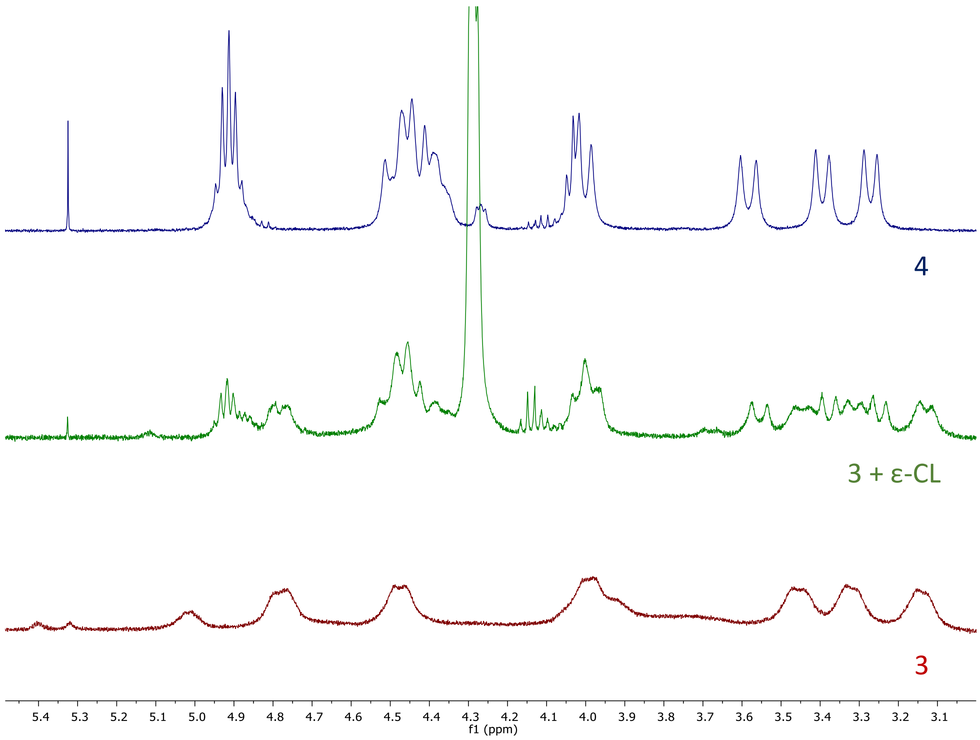

Figure S66. Stacked ^{1}H NMR spectra of **3** (red, bottom), **3** with addition of excess ε-CL (green, middle), and **4** (blue, top) in $CDCl_3$ at 233 K. The broad singlet at δ_H= 4.25 ppm corresponds to excess free ε-CL

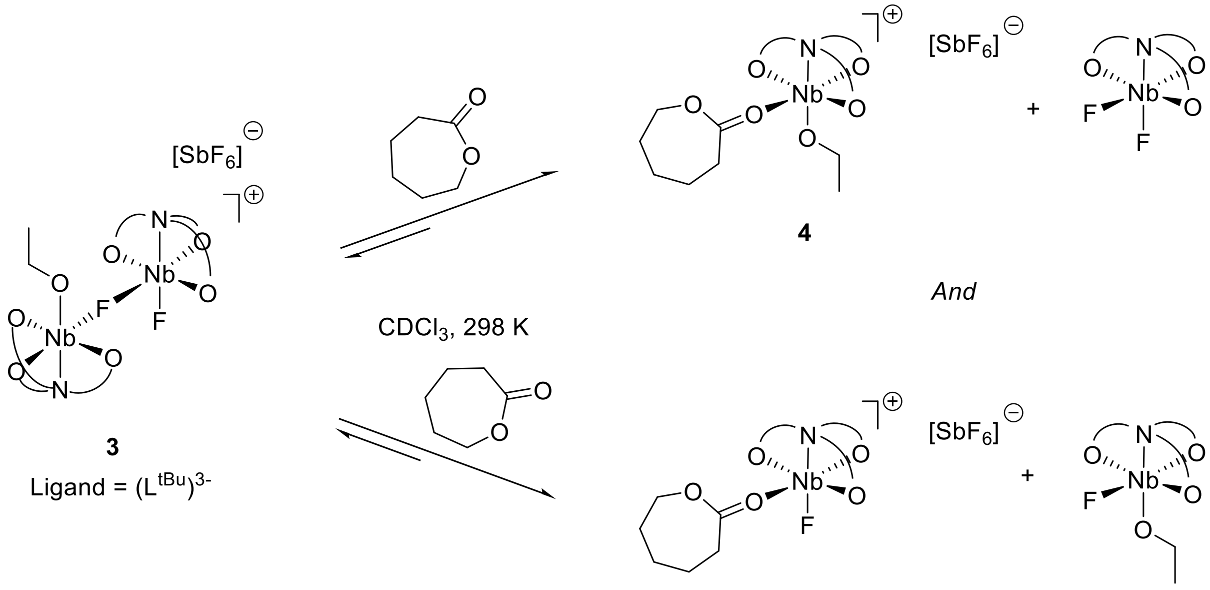

Scheme S1. Treatment of **3** with excess ε-CL at 298 K yields in equilibrium **4**, and other species, by cleavage of Nb-μ_2F bonds

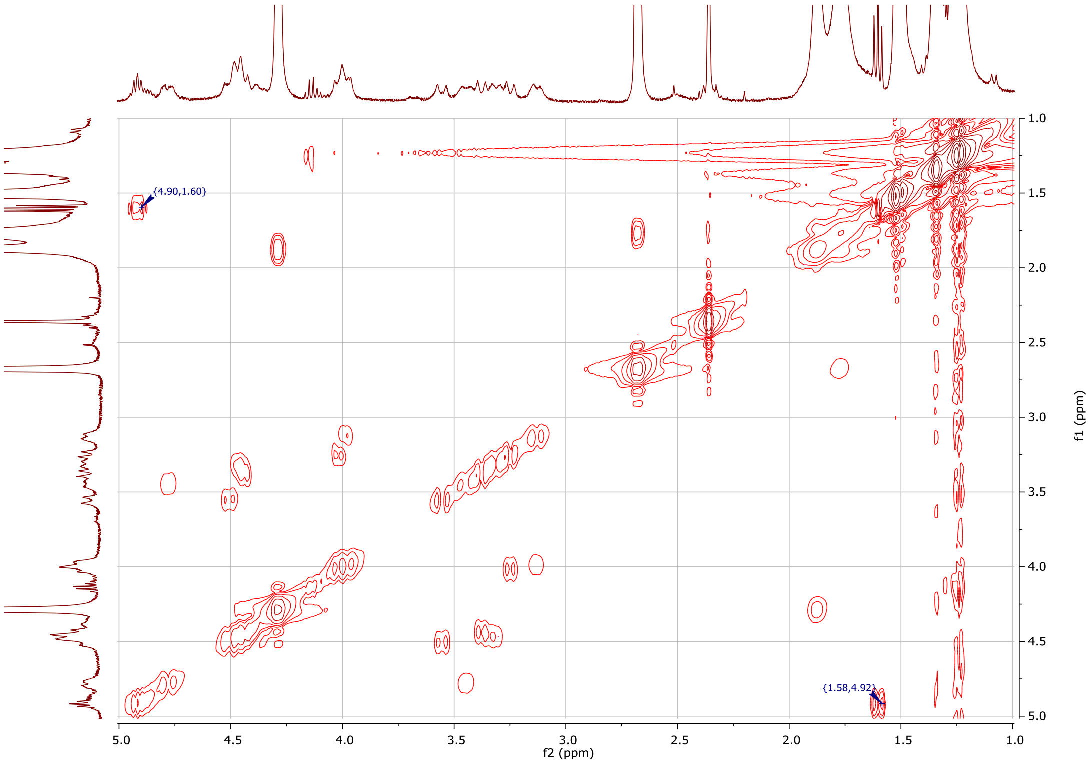

Figure S67. COSY NMR spectra of **3** with addition of excess ε-CL in $CDCl_3$ at 233 K, showing alkoxide resonances characteristic of **4**

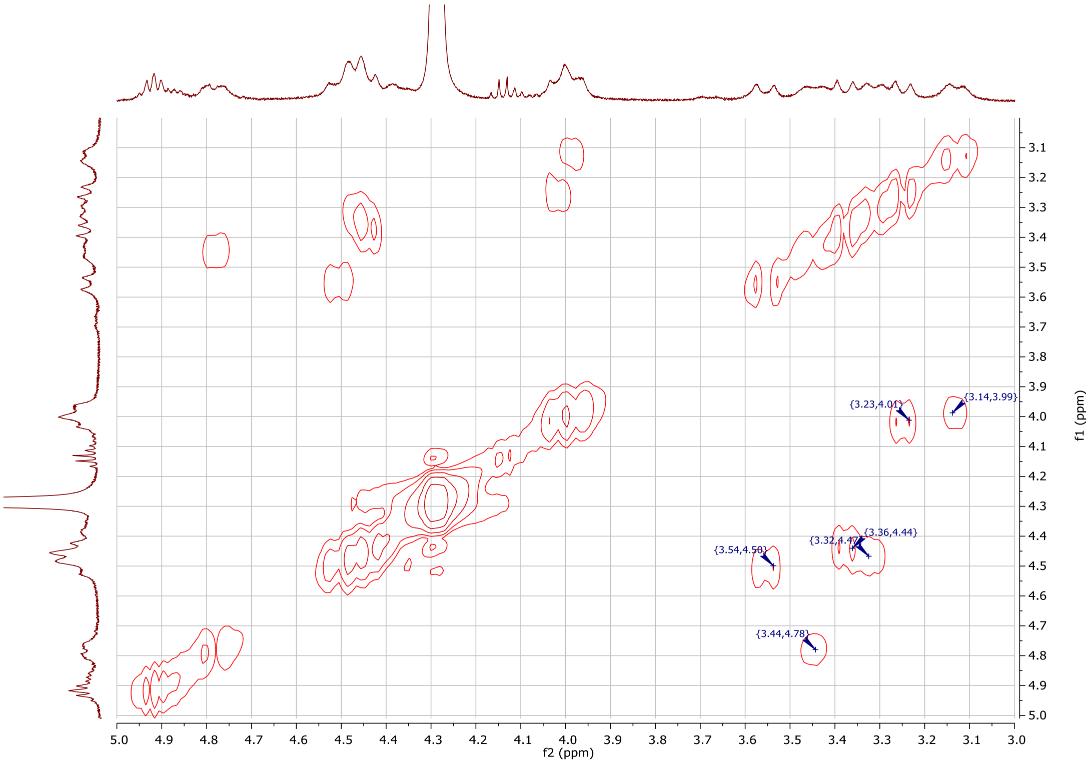

Figure S68. Methylene region of the COSY NMR spectra of **3** with addition of excess ε-CL in $CDCl_3$ at 233 K, showing increased number of NC*H*H resonances

Addition of *L*-lactide to Nb complex 3

On addition of excess *L*-LA to **3** at ambient temperature, little significant change was seen in the ^{1}H NMR spectrum at -40 °C. This suggests that lactide does not coordinate to a Nb centre or promote cleavage of the dinuclear complex, in the manner that ε-CL does. Although polymerisations were attempted using *rac*-LA, L-LA was used here in order to ensure that ^{1}H NMR spectra were as simple as possible. Whilst the literature contains examples of ROP catalysts that are deactivated by lactide, this typically occurs after one equivalent of that monomer has been ring-opened, the linear lactyl dimer then chelating the metal centre.[2–4] In this work, *L*-LA appears not to coordinate at all, which we surmise is due to steric congestion arising from the bulky amine tris(phenolate) ligand scaffold, and the methyl groups of *L*-LA.[5]

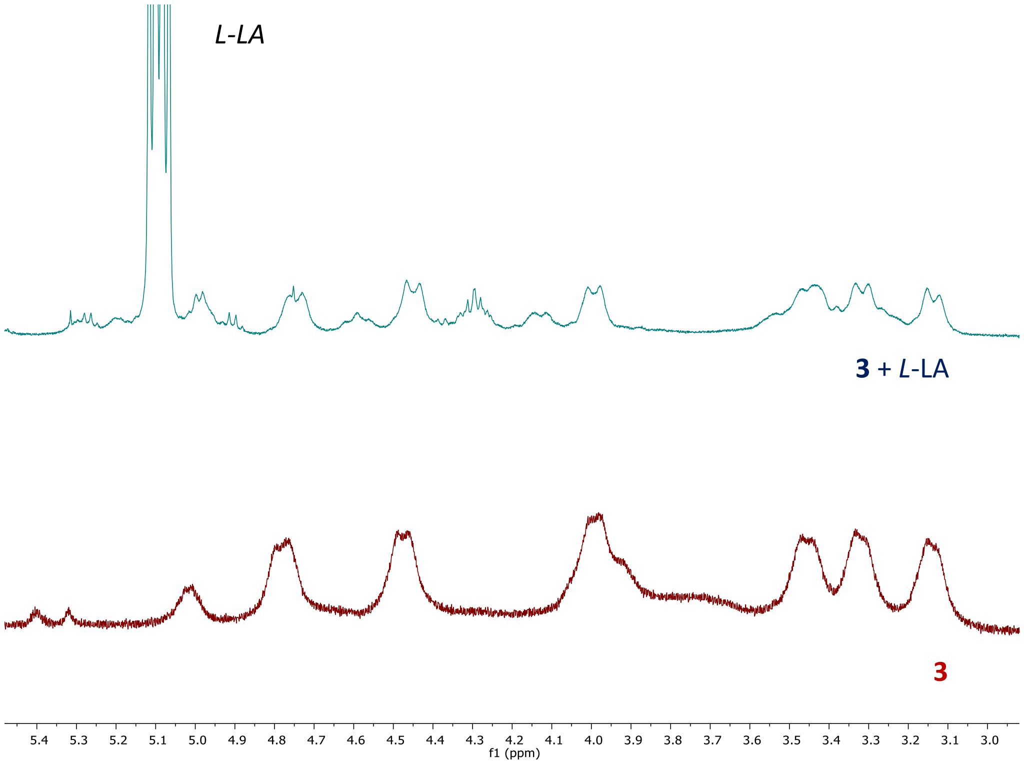

Figure S69. Stacked ^{1}H NMR spectra of **3** (red, bottom), **3** with addition of excess *L*-LA (blue, top) in $CDCl_3$ at 233 K, showing persistence of **3**. The resonance at δ_H= 5.1 ppm corresponds to the methine proton of free *L*-LA

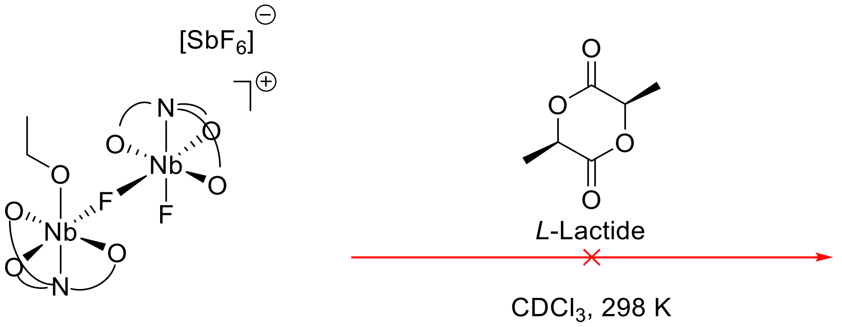

3

Ligand = $(L^{tBu})^{3-}$

Scheme S2. Treatment of **3** with excess *L*-LA at 298 K yields no significant change in the ^{1}H NMR spectrum

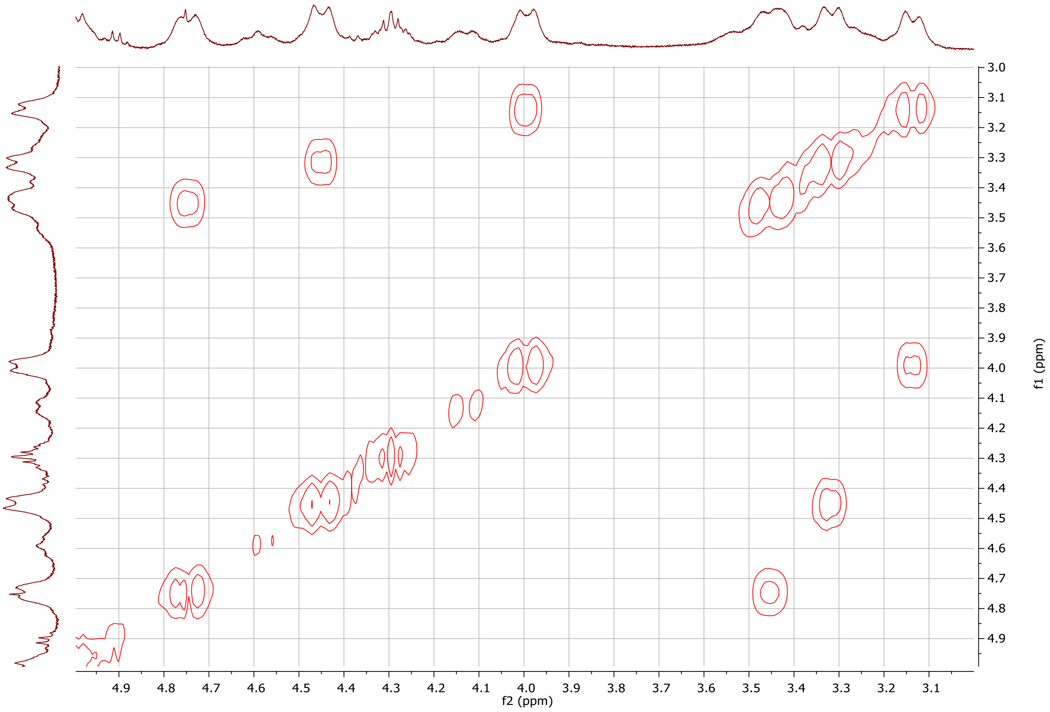

Figure S70. COSY NMR spectra of **3** with addition of excess *L*-LA in $CDCl_3$ at 233 K, showing persistence of **3**

Addition of ε-caprolactone to Nb complex 7

On addition of excess ε-CL to **7**, the ^{1}H NMR spectrum acquired at -60 °C in $CDCl_3$ is well-resolved, whereas that of **7** in the absence of ε-CL appears highly fluxional. The observed change is compatible with cleavage of the dinuclear species and coordination of ε-CL to the remaining cationic Nb centre, as described for the analogous system **5**. Although the proposed product $[L^{tBu}Nb(OEt)(\varepsilon\text{-CL})]^+[SbF_6]^-$, **8**, appears sufficiently stable for ^{1}H NMR analysis, efforts to directly synthesize (in analogy to the preparation of **4**), isolate, or crystallise it were repeatedly unsuccessful, this likely being attributable to the reduced kinetic stabilization provided by ancillary ligand $(L^{Me})^{3-}$, relative to the bulkier scaffold $(L^{tBu})^{3-}$. The two well-resolved alkoxide O*CH₂* signals presumably correspond, respectively, to the neutral and cationic fragments formed on cleavage of **7**. Whilst those monometallic species may exist in equilibrium with **7**, this can not be seen in the ^{1}H NMR spectrum, due to the extreme fluxionality of ^{1}H environments in **7**.

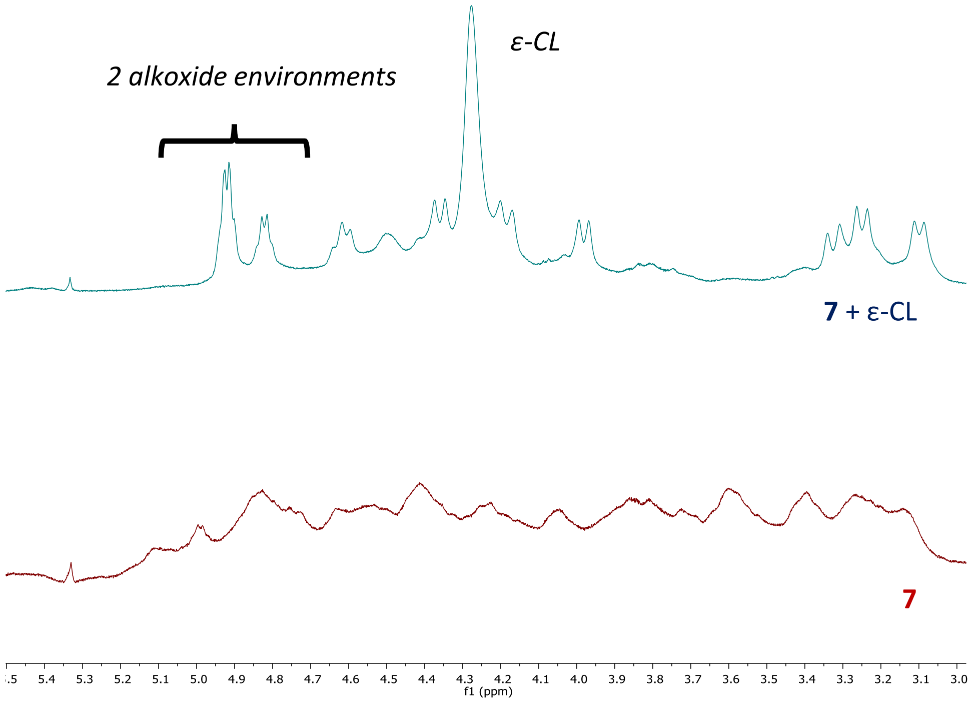

Figure S71. Methylene region of the ^{1}H NMR spectrum of **7** in $CDCl_3$ at 213 K (bottom) and of the same species following addition of excess ε-CL (top), showing well-resolved signals. The broad singlet at δ_H= 4.25 ppm corresponds to excess free ε-CL

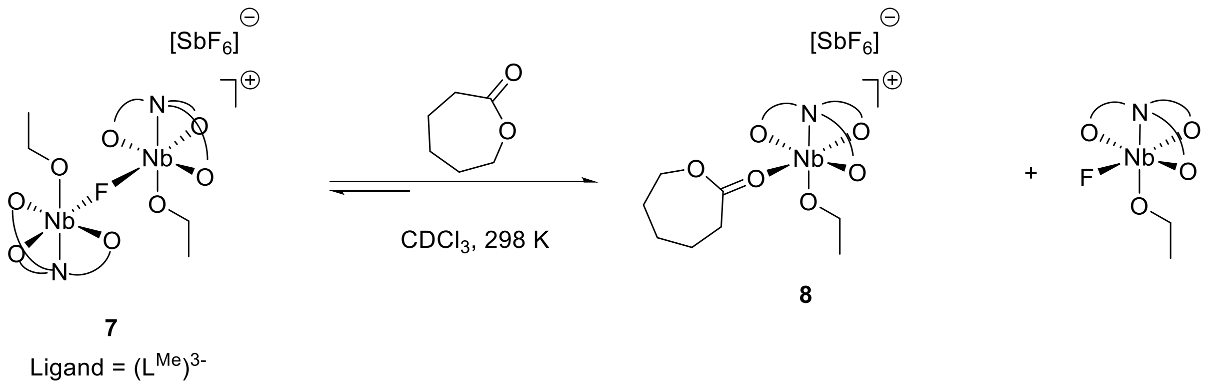

Scheme S3. Treatment of **7** with excess ε-CL at 298 K yields in equilibrium **8**, by cleavage of Nb-μ_2F bonds

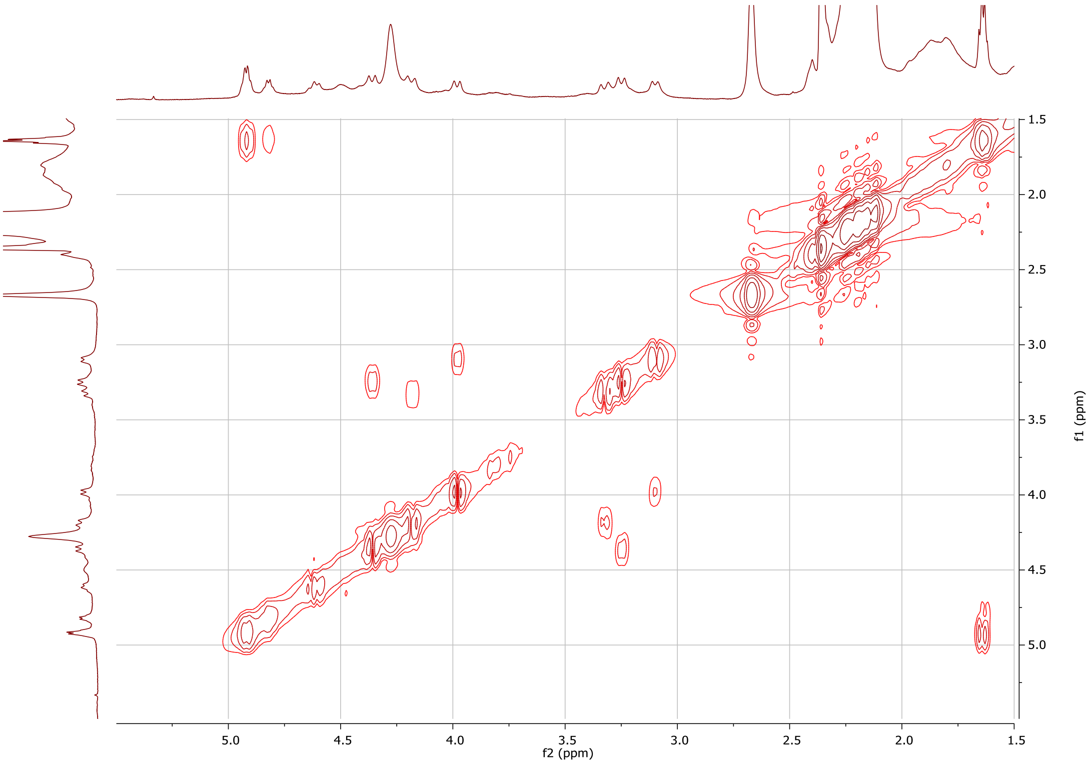

Figure S72. COSY NMR spectrum of **7** with excess ε-CL (top), in $CDCl_3$ at 213 K. The broad singlet at δ_H= 4.25 ppm corresponds to excess free ε-CL

Heating Nb complex 4

In a sealed J Young's NMR tube, a solution of **4** in $CDCl_3$ was heated stepwise, and 1H NMR spectra acquired periodically at ambient temperature. As expected, degradation occurred, presumably *via* insertion of the coordinated ε-CL moiety into the metal-alkoxide bond. Moreover, the alkoxide OCH_2 signal is seen to move downfield with heating. A signal is also seen to grow in at δ = 5.18 ppm, integrating in a 1:1 ratio with the shifted ethoxy OCH_2 signal (previously alkoxide), which we attribute to the metal-coordinated polymer chain-end OCH_2 protons. A signal also appeared at δ = 4.35 ppm, corresponding to the OCH_2 protons of the polymer backbone arising from ROP of residual uncoordinated ε-CL contaminating the sample of **4**, yielding PCL with d.p > 1. A white precipitate slowly formed as the sample was heated. We tentatively suggest that in the absence of further equivalents of ε-CL to occupy the vacant coordination site following intramolecular nucleophilic attack, the resulting unstable species undergoes degradation in $CDCl_3$. Accordingly, the signal at δ = 4.35 ppm corresponding to PCL with >1 ε-caprolactyl repeat unit is larger than might be expected, that material remaining in solution as the concentration of cationic Nb species is decreased as a result of decomposition. Such decomposition was not apparent in catalytic use in toluene-d_8.

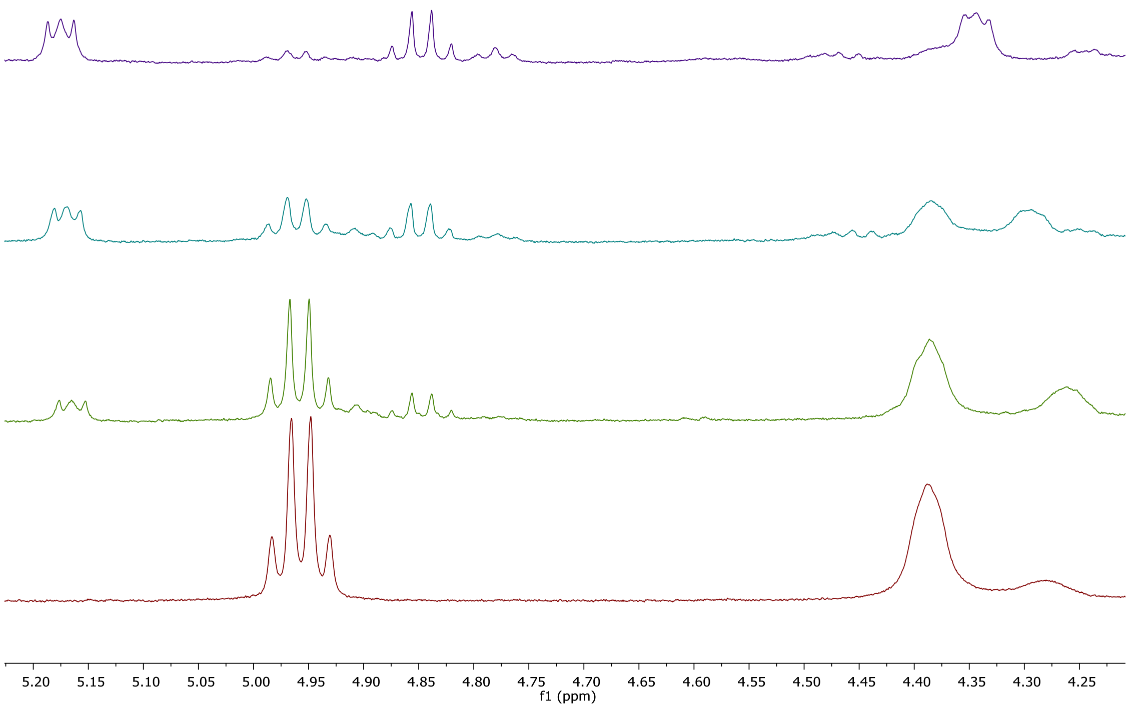

Figure S73. Methylene region of the ^{1}H NMR spectra of **4** (red, bottom) and of the same species after being heated to 65 °C for 30 minutes followed by 75 °C for; 45 minutes (green), 105 minutes (blue) and 165 minutes (purple, top) acquired in $CDCl_3$ at 298 K

Polymerisation studies

General polymerisation procedure

In the glove box, a J Young's polymerisation tube (Schlenk flask) containing a magnetic stirring bead was charged with the solid catalyst. Then, 2.0 ml dry SPS toluene was added, followed by 200 mg ε-CL (resulting in a monomer concentration of 0.80 mol dm^{-3}). The flask was sealed, removed from the glove box and placed into a silicone oil bath heated to 80 °C. After the desired reaction time (typically 6 hours) had passed, the flask was cooled to 298 K, opened, and quenched by addition of 10 mL wet dichloromethane. Then the solvent was removed with a rotary evaporator, and the remaining material retained for analysis. Conversion was determined by integration of the ^1H NMR methylene resonances corresponding to monomer and polymer species, at δ=4.20 ppm and δ=4.05 ppm, respectively, in $CDCl_3$. Polymer molecular weight (g mol^{-1}) was determined *via* gel permeation chromatography (GPC), using a refractive index (RI) detector calibrated against polystyrene standards. Molecular weight values are reported after application of a conversion factor of 0.56.[6,7]

Where analysis of the polymer end group was required, the reaction was carried out at a scale of 500 mg with respect to the monomer. After removal of solvent, the polymer product in those cases was precipitated and stirred for 20 hours in methanol (25 ml) to remove catalyst-derived species (in particular any persistent Nb ethoxide species, or ethanol), and the resulting white powder isolated by vacuum filtration over a glass frit. This was then washed with a further 50 ml methanol, and dried under dynamic vacuum for 2 hours. The resulting purified polymer was then analysed with GPC and the end group quantified *via* ^1H NMR spectroscopy. This was achieved by integration of the methylene signals corresponding to the polymer backbone and EtO- end-group, appearing at δ=4.05 ppm and δ=4.12 ppm, respectively, in $CDCl_3$. Due to the partial superimposition of the two resonances, only the two most down-field peaks of the well-resolved quartet corresponding to the EtO- end group were integrated, and the value doubled to give the integrated area of the whole signal.

End-group analysis

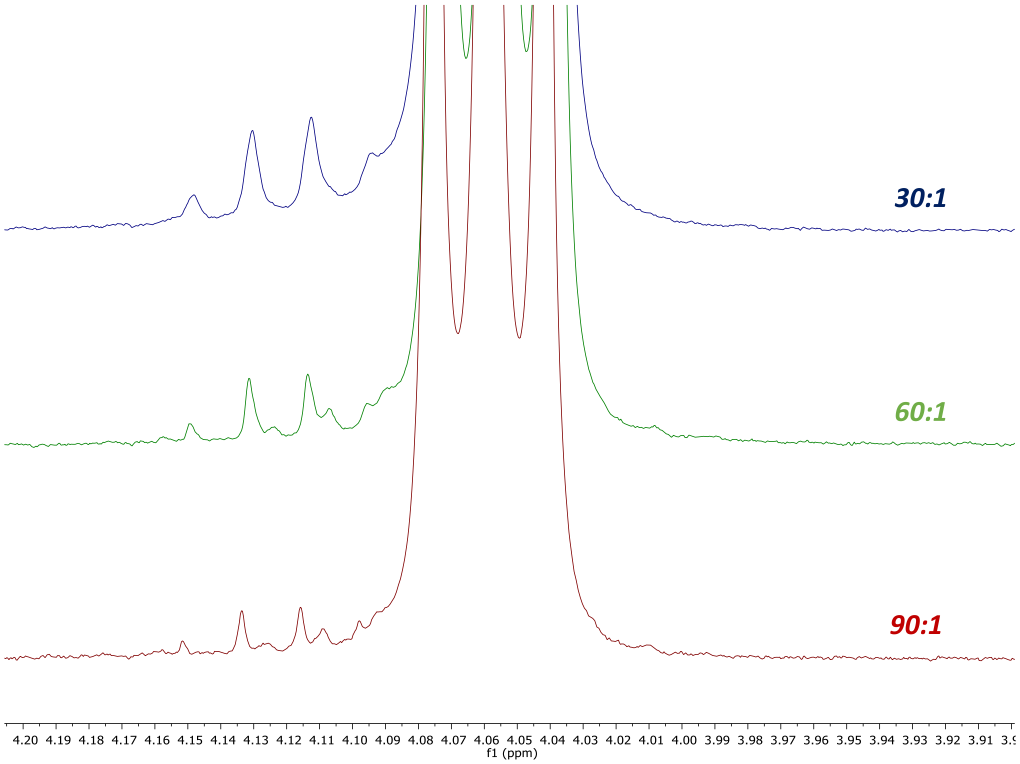

Figure S74. Stacked ^{1}H NMR spectra of purified PCL$^-$ produced in the presence of initiator **4** (3.33 mol% blue, top; 1.67 mol% green, middle; and 1.11 mol% red, bottom) showing polymer backbone (OCH_2) and ethoxide end-group (OCH_2) signals (at $\delta_H = 4.05$ ppm and $\delta_H = 4.12$ ppm, respectively), acquired in $CDCl_3$ at 298 K. Due to overlap of polymer backbone and end-group signals, the end group abundance was calculated from the two well-resolved down-field peaks of the quartet.

Table S1. Polymerization data for the ROP of ε-CL in the presence of each cationic Nb(V) species, for analysis of polymer end-groups, including catalyst loading and abundance of ethoxy end-groups, determined *via* ^{1}H NMR. Data corresponds to products of polymerization reactions described in Tables 1-3 of the main text.

Entry	*Pre-catalyst*	*[ε-CL]:[Pre-catalyst]*	^{a}*[ε-CL]:[OEt]; ^{1}H NMR*	^{b}*Conversion, %*	$^c M_n^{GPC}$	$^{d,e} M_n^{Theo}$	$^f M_n^{NMR}$
1	**3**	30:1	33:1	>99	5000	^{d}3470	3813
2	**3**	60:1	64:1	>99	7350	^{d}6894	7351
3	**4**	30:1	28:1	>99	4900	^{e}3585	3242
4	**4**	60:1	64:1	>99	8150	^{e}7007	7351
5	**4**	90:1	86:1	>99	12000	^{e}10435	9862
6	**7**	30:1	18:1	>99	3150	^{d}3471	2101
7	**7**	60:1	35:1	>99	6750	^{d}6894	4041

Conditions: 0.80 mol dm^{-3} ε-CL solution in toluene, 80 °C for six hours, in the presence of cationic Nb(V) pre-catalyst. ^{a} Ratio determined from relative integrations of signals at δ=4.05 ppm and δ=4.12 ppm in the ^{1}H NMR spectrum of the polymer product, corresponding to OCH_2 methylene protons of the polymer backbone and EtO-/H- end-group, respectively. ^{b} Conversion determined *via* ^{1}H NMR spectroscopy, by integration of the monomer and polymer OCH_2 methylene resonances. ^{c} Determined *via* GPC analysis in THF using a refractive index detector calibrated against polystyrene standards, and with application of a conversion factor of 0.56.[6,7] ^{d} M_n^{Theo} for polymerizations in the presence of **3** or **7**, calculated from conversion and catalyst concentration, assuming one initiation event *per* molecule of initiator $\left\{\left(M_{r,\varepsilon-CL} \times \frac{\%_{conv}}{100} \times \frac{[\varepsilon-CL]}{[Cat.]}\right) + M_{r,EtOH}\right\}$. ^{e} M_n^{Theo} for polymerizations in the presence of **4**, calculated from conversion and catalyst concentration, assuming one initiation event *per* molecule of initiator $\left\{\left(M_{r,\varepsilon-CL} \times \frac{\%_{conv}}{100} \times \frac{[\varepsilon-CL]}{[Cat.]}\right) + M_{r,EtOH} + M_{r,\varepsilon-CL}\right\}$. ^{f} M_n^{NMR} calculated from the ratio of integrated ^{1}H NMR signal areas A_{PCL} and A_{EtO-}, corresponding to the polymer backbone, and EtO- end group, respectively, $\left\{\left(M_{r,\varepsilon-CL} \times \frac{A_{PCL}}{A_{EtO-}}\right) + M_{r,EtOH}\right\}$.

End group analysis *via* ^{1}H NMR spectroscopy (Figure S74, Table S1) showed that, whilst **3** and **4** yielded a ratio of ε-caprolactyl polymer backbone and ethoxy end group OCH_2 methylene signals corresponding to initiation of one polymer chain per molecule of the respective Nb complex, for **7**, the concentration of ethoxy polymer end groups corresponded to two initiation events occurring *per* molecule of the pre-catalyst. This is compatible with the proposed active species $[L^{Me}Nb(OP)(\varepsilon\text{-}CL)]^+[SbF_6]^-$ (an analogue of **8**, where OP is the growing polymer chain) and neutral fragment $[L^{Me}NbF(OP)]$ existing in equilibrium with bimetallic species $[\{L^{Me}Nb(OP)\}_2\text{-}\mu_2F]^+[SbF_6]^-$ (an analogue of **7**) (Scheme S4). Neutral Nb(V) fluoro complex $[L^{Me}NbF(OP)]$ is structurally analogous to Nb(V) chloro complex **6**, which is entirely inactive for the ROP of ε-CL under our experimental conditions (see "Control reactions", below). It is therefore implausible that ROP activity of $[L^{Me}NbF(OP)]$ could account for half of the polymer chains in a PCL sample produced in the presence of pre-catalyst **7**. Such an equilibrium process as that described by Scheme S4 would permit both alkoxide moieties of **7** to enter the catalytic manifold *via* reversible, asymmetric cleavage of the bimetallic species at either Nb-μ_2F bond in the presence of ε-CL, to form active catalyst $[L^{Me}Nb(OP)(\varepsilon\text{-}CL)]^+[SbF_6]^-$.

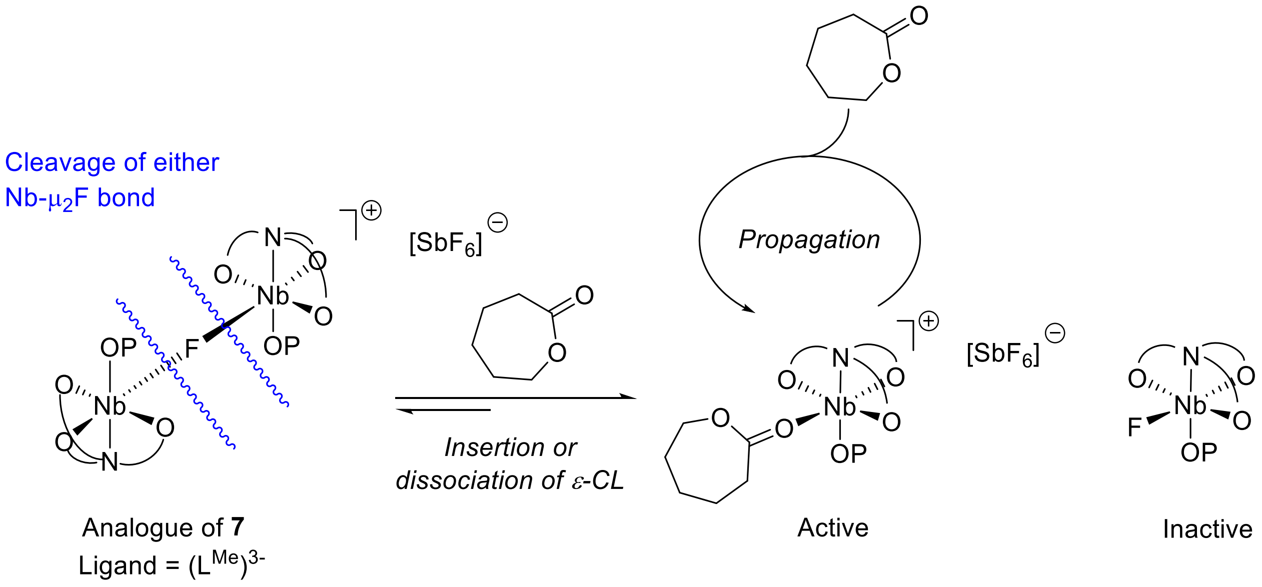

Scheme S4. Proposed equilibria between active and inactive species on use of presence of pre-catalyst **7**, permitting initiation by all alkoxide moieties.

In this scenario, a maximum of half of the alkoxide groups would be active at a given time. Additionally, the reversible formation of the bimetallic species $[\{L^{Me}Nb(OP)\}_2\text{-}\mu_2F]^+[SbF_6]^-$ must be non-rate-determining in order to be compatible with living or immortal polymerization kinetics. Furthermore, as the steric demands of the growing polymer chain increase, the reformation of a bimetallic species is likely to become kinetically unfavourable. Therefore, it is expected that after oligomer formation, the portion of the growing chains corresponding to the dormant neutral species would be unable to re-enter the catalytic cycle. Although their ethoxy end groups would be detectable by NMR spectroscopy, such oligomers would not be readily characterised *via* GPC. Moreover, continued growth of the remaining active chains would consume the rest of the monomer feed, resulting in polymer of only slightly reduced molecular weight, relative to if only one of the two pre-catalyst alkoxide moieties was active. Comparison of predicted and experimental molecular weight (M_n^{GPC}) values for PCL samples produced in the presence of **7** (Table 3 in main paper, Entries 6, 7), in addition to the quantification of ethoxy end groups *via* ^{1}H NMR spectroscopy, supports this mechanistic scenario.

The monoalkoxide species, **3**, presumably undergoes similar dynamic behaviour to **7** under polymerization conditions, although the asymmetric nature of **3** is compatible only with more complex equilibria (Scheme S5).

The slowest catalyst system was **7**. In addition to reversible deactivation, we attribute this to the decreased lability of the Nb-alkoxide bond of **7**, relative to **3**, demonstrated by the retention of alkoxide moieties at both metal centers.

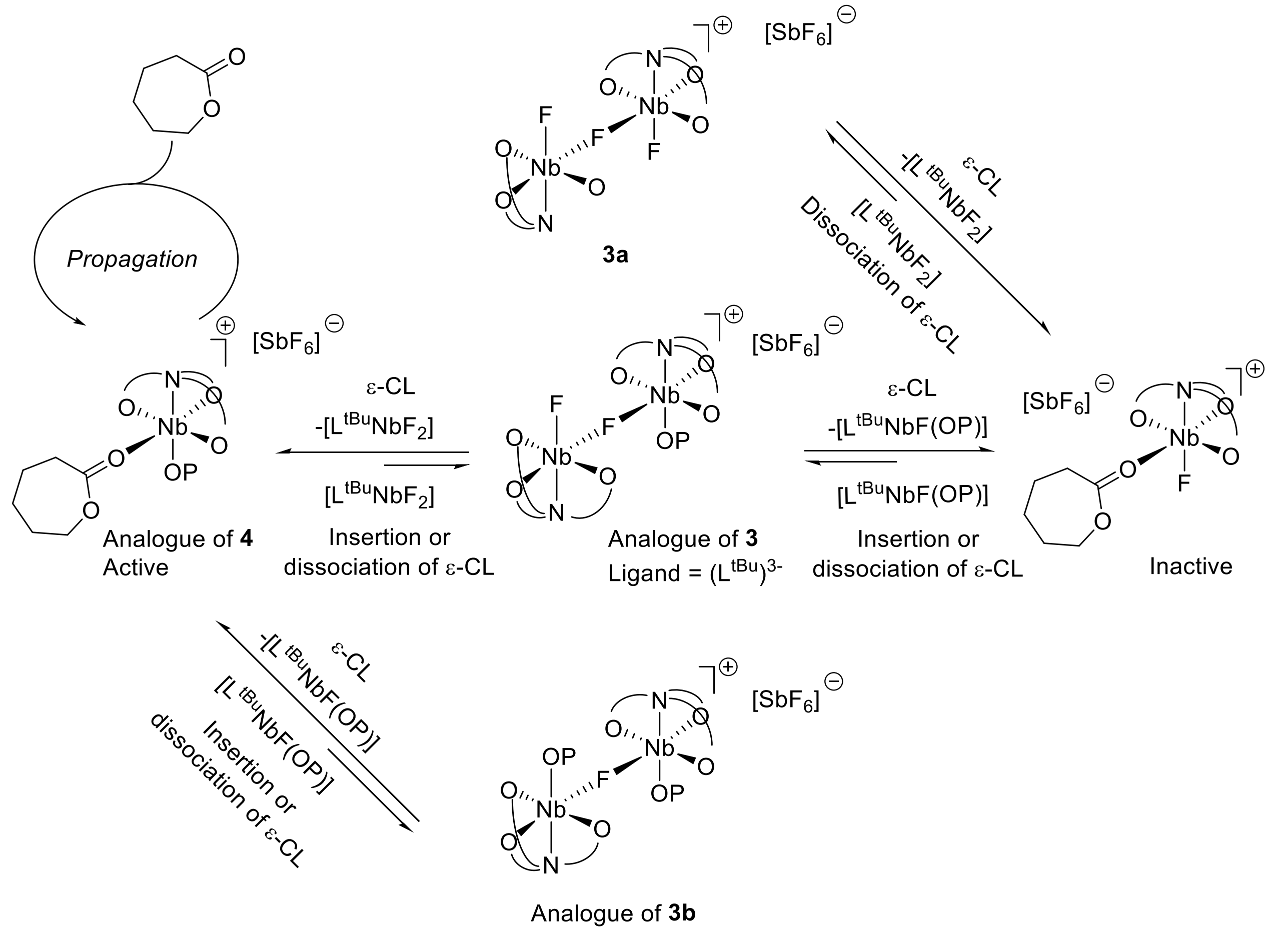

Scheme S5. Proposed equilibria between active and inactive species on use of pre-catalyst **3**

Kinetic studies of ε-caprolactone polymerisation

General procedure for kinetic studies

In the glove box, a J Young's NMR tube was charged with the solid initiator, **3**, **4** or **7**. Then, 1.0 ml dry toluene-d_8 was added, followed by 100 mg ε-CL (resulting in a monomer concentration of 0.80 mol dm^{-3}). The tube was sealed, removed from the glove box and heated to 80 °C inside the NMR spectrometer. The temperature was maintained for 16 hours, and ^1H NMR spectra were acquired at thirty-minute intervals for the first 12 hours. After the desired reaction time had passed, the tube was cooled, opened, and the polymer worked up and analysed as described in the general polymerisation procedure, above. The reaction progress was monitored by observation of ^1H NMR methylene resonances corresponding to monomer and polymer species, at δ = 3.66 ppm and δ = 4.02 ppm, respectively. For determination of the observed rate constant k_{obs}, for each catalyst, semi-logarithmic plots were constructed by plotting –ln(1-[PCL]) against time, where [PCL] is the concentration of PCL, between 30 mins and 300 mins. For dinuclear, monocationic pre-catalysts **3** and **7**, no more than half of the Nb centers in each case can be simultaneously represented by active cationic species. This corresponds to a maximum of one active growing chain *per* dinuclear pre-catalyst molecule, at any given time, permitting valid comparison of the rates of ROP in the presence of initiators **3** and **7**.

TOF values, in h^{-1}, were calculated in each case for the period of the reaction falling between the data points closest in value to 15% and 65% conversion, respectively, assuming a constant rate between those points. TOF = $(\Delta[\varepsilon\text{-CL}]/\Delta t)\times([\varepsilon\text{-CL}]_0/[\text{Cat}])$. The values obtained were: Complex **3** = 38.6 h^{-1}; Complex **4** = 51.4 h^{-1}; Complex **7** = 20.9 h^{-1}.

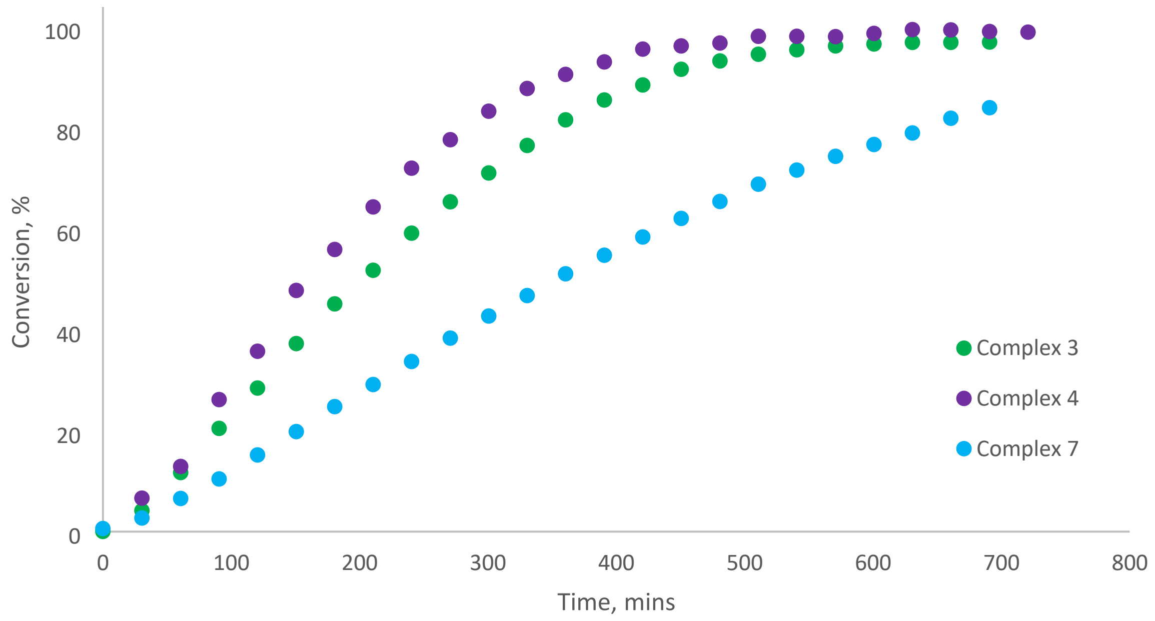

Figure S75. Plots of Conversion versus time for the ROP of ε-CL in the presence of initiators **5**-**7** (0.4 mol%), Conversion determined *via* ^1H NMR spectroscopy, by integration of the monomer and polymer OCH_2 methylene resonances

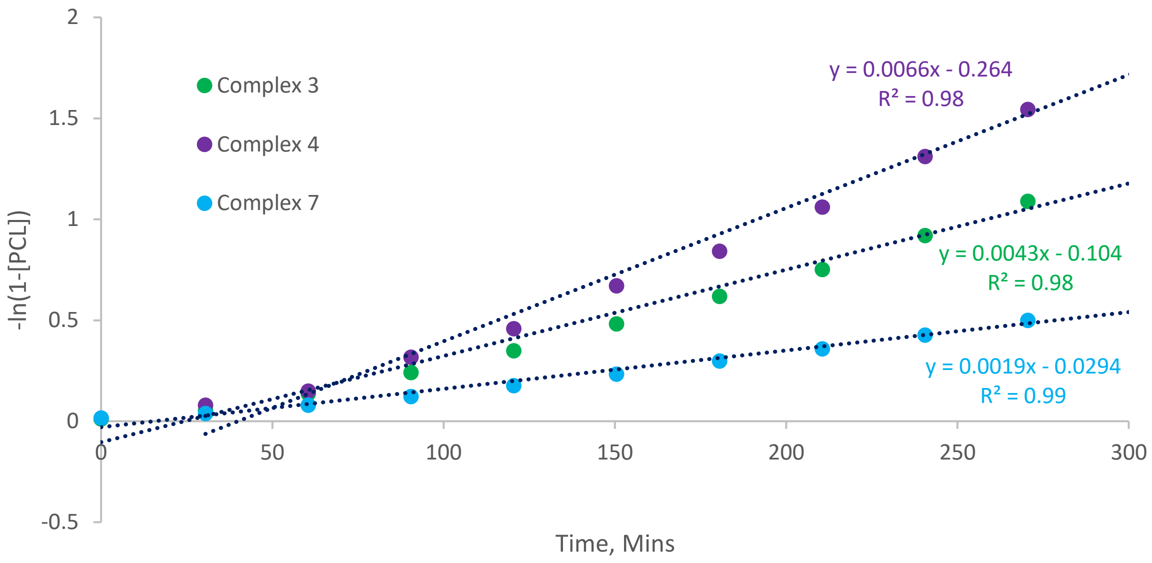

Figure S76. Semi-logarithmic initial rate plots, corresponding to the polymer concentration, for the ROP of ε-CL, in the presence of 0.4 mol% of each cationic Nb(V) species in toluene-d_8 at 80 °C.

3, **4** and **7** were all empirically observed to afford *pseudo*-first order kinetics with respect to the monomer in the ROP of ε-CL. Of the three cationic initiators, **4** produced the highest rate in the ROP of ε-CL. **3** appeared slightly slower. The rate difference between **3** and **4** may be attributed to inhibition of ε-CL coordination at the metal of the monometallic active species by equilibrium formation of bimetallic species (analogues of **3**, **3a**, **3b**) (Scheme S5, above). The rate difference between **3** and **4** cannot be due to a rate-determining cleavage event during initiation, as this would entail a departure from living polymerization kinetics, and a dramatic broadening of the polymer molecular weight distribution, as well as a significant induction period in the case of **3**, none of which were observed. It should be noted, however, that the relatively modest difference in the rates afforded by **3** and **4**, respectively, may result in some part to an error in the catalyst loadings, as the complexes were dosed as solid materials on the mg scale, due to poor solubility in toluene-d_8 at ambient temperature. Furthermore, the kinetic study of the ROP of ε-CL in the presence of **4** was carried out using a different spectrometer from those reactions in which **3** and **7** were used. Variation in temperature calibration may therefore be anticipated to influence the rate difference between **3** and **4**. It is more significant, therefore, that the rate of ROP in the presence of **7** was much lower than that of **3**. We attribute this to the decreased lability of the alkoxide moiety of **7**, relative to that of **3**, as indicated by the retention of alkoxides at both Nb centers of **7**, compared to substitution of one alkoxide for a terminal fluoro ligand in **3**.

Polymer characterisation data for kinetic studies

Table S2. Polymerisation data for the NMR-scale ROP of ε-CL in the presence of cationic Nb complexes, for kinetic studies

Entry	*Pre-Catalyst*	*Conversion, %*[a]	[b]M_n^{GPC}	[b]M_w	[b]$Đ_M$	[c,d]M_n^{Theo}	k_{obs}, min^{-1}	*TOF*, h^{-1}
1	**3**	>99	9650	13500	1.40	[c]28581	0.0043	38
2	**4**	>99	22550	39250	1.74	[d]28695	0.0066	49
3	**7**	97	13950	18850	1.36	[c]27725	0.0019	21

Conditions: 0.80 mol dm^{-3} ε-CL solution in toluene-d_8 (100 mg in 1 ml), 80 °C for sixteen hours, in the presence of 0.4 mol% pre-catalyst. [a] Conversion determined *via* ^{1}H NMR spectroscopy, by integration of the monomer and polymer OCH_2 methylene resonances. [b] Determined *via* GPC analysis in THF using a refractive index detector calibrated against polystyrene standards, and with application of a conversion factor of 0.56. [c]M_n^{Theo} for polymerizations in the presence of **3** or **7**, calculated from conversion and catalyst concentration, $\left\{\left(M_{r,\varepsilon-CL} \times \frac{\%_{conv}}{100} \times \frac{[\varepsilon-CL]}{[Cat.]}\right) + M_{r,EtOH}\right\}$. [d] M_n^{Theo} for polymerizations in the presence of **4**, calculated from conversion and catalyst concentration, $\left\{\left(M_{r,\varepsilon-CL} \times \frac{\%_{conv}}{100} \times \frac{[\varepsilon-CL]}{[Cat.]}\right) + M_{r,EtOH} + M_{r,\varepsilon-CL}\right\}$.

Control reactions

Attempted polymerisation of *rac*-lactide

Complexes **3** and **4** were both also assessed with respect to the ROP of *rac*-LA under identical reaction conditions to those used for the ROP of ε-CL. Both **3** and **4** were entirely inactive. We have demonstrated that, unexpectedly,[2–5] **3** does not interact with *L*-LA, as it does with ε-CL to form monometallic adduct **4** (see "Addition of *L*-LA to Nb complex 3", above). Accordingly, coordination-insertion and activated monomer mechanisms of ROP are not accessible for lactide with this system.

Table S3. Polymerisation data for the attempted ROP of *rac*-LA in the presence of **3** and **4**, respectively.

Entry	*Initiator*	*Scale (monomer), mg*	[a]*Conversion, %*
1	**3**	200	0
2	**4**	250	0

Conditions: 0.63 mol dm^{-3} *rac*-LA solution in toluene, 80 °C for sixteen hours, in the presence of 1 mol% pre-catalyst. [a] Conversion determined *via* ^{1}H NMR spectroscopy, by integration of the monomer and polymer methine resonances.

Attempted polymerisation of ε-CL in the presence of neutral Nb(V) complexes

The neutral Nb complexes **1**, **2**, **5**, and **6**, and $AgSbF_6$, synthetic precursors to the cationic Nb pre-catalysts, variously exhibited no activity or extremely low activity for the polymerisation of ε-CL under the relevant conditions. The inactivity of $AgSbF_6$ confirms, in addition to our mechanistic studies and end-group analysis, that the ROP of ε-CL in the presence of cationic Nb complexes is not catalysed by the SbF_6^- counterion.

Table S4. Control reactions of ε-CL in the presence of various synthetic precursors to cationic pre-catalyst species under polymerisation conditions

Entry	*Catalyst*	*Catalyst Loading, mol%*	*BnOH Loading, mol%*	*Duration, hours*	*Conversion, %[a]*
1	**1**	1.00	0.00	16	11
2	**2**	1.00	1.00	16	22
3	**5**	1.00	0.00	6	0
4	**6**	1.00	0.00	6	0
5	$AgSbF_6$	3.30	3.30	16	5

Conditions: 200 mg ε-CL, in 0.80 mol dm^{-3} solution in toluene, 80 °C. [a] Conversion determined *via* ^1H NMR spectroscopy, by integration of the monomer and polymer methylene resonances.

Polymerisation of ε-caprolactone in the presence of *L*-lactide

General procedure

In the glove box, a J Young's NMR tube was charged with the solid initiator, **4** (20 mg, 2 mol%). Then, 1.0 ml dry toluene-d_8 was added, followed by 100 mg ε-CL (resulting in a monomer concentration of 0.80 mol dm^{-3}) and 126 mg *L*-LA (0.80 mol dm^{-3}). The tube was sealed, removed from the glove box and heated to 80 °C inside the NMR spectrometer. The temperature was maintained for 16 hours, and ^1H NMR spectra were acquired at ten-minute intervals for the first 12 hours. After the desired reaction time had passed, the tube was cooled, opened, and the solvent removed under dynamic vacuum. The crude reaction mixture was then dissolved in $CDCl_3$, and a ^1H NMR spectrum acquired at 298 K. No further analysis of the product was carried out. The reaction progress was monitored by observation of ^1H NMR methylene resonances corresponding to monomer and polymer species, at δ = 3.75 ppm and δ = 3.98 ppm, respectively.

Kinetic data and analysis

Homopolymerization of ε-CL was observed in the presence of *L*-LA, which was not polymerized (Figures S77, S78). The *L*-LA methine proton signal shifted significantly from δ = 4.67 ppm to δ = 4.42 ppm during the polymerization (Figure S77). NMR titration of *L*-LA against ε-CL under the polymerization conditions (80 °C in toluene-d_8) in the absence of an initiator showed that the shift in the *L*-LA signal was due to the changing ε-CL concentration (Figure S79). The absence of any observable shift when a second titration was carried out, of *L*-LA against PCL confirmed this to be the case. Notably, whilst selectivity toward homopolymerization of ε-CL was retained, the presence of *L*-LA did produce a much lower rate than was anticipated at this catalyst loading. As *L*-LA has not been observed to interact with any of the Nb(V) complexes reported herein, we attribute this retardation of rate to hydrogen bonding between *L*-LA and ε-CL in competition with coordination of ε-CL to the metal centre. Although the concentration of *L*-LA did decrease slightly during the course of the reaction, this empirically appears to be zero-order with respect to the monomer, and is attributed to a non-catalytic ring-opening event, evidence for which can be seen in the ^1H NMR spectrum of the crude product mixture in $CDCl_3$ at 298 K (Figure S80).

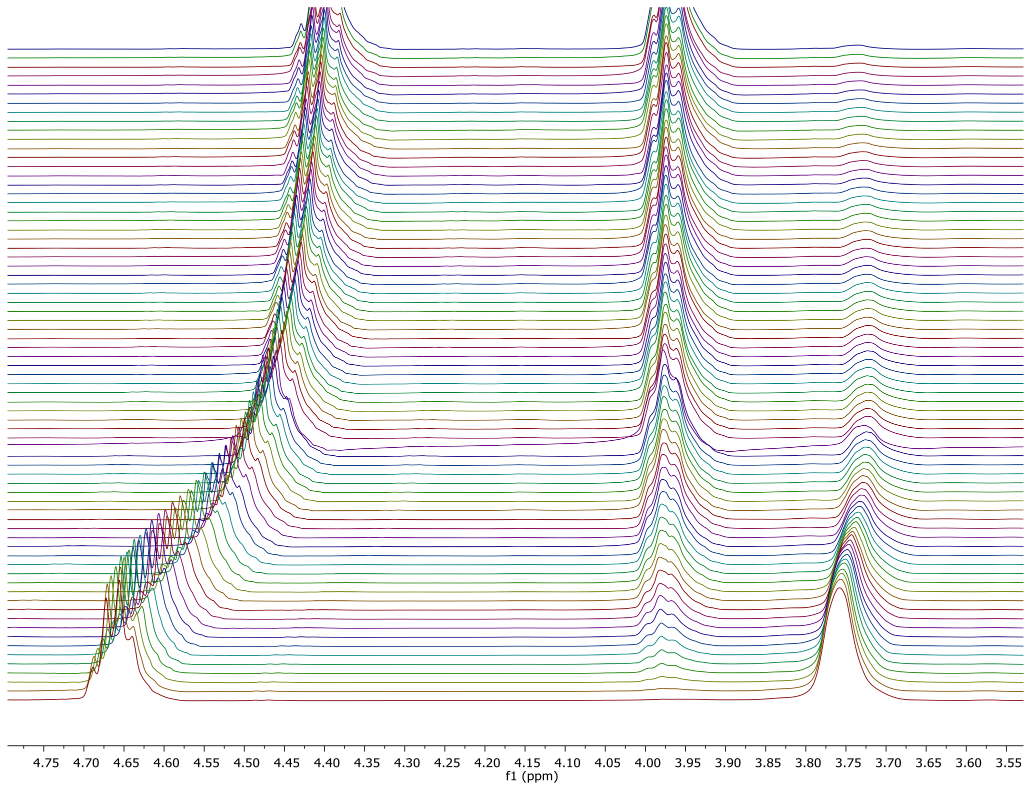

Figure S77. Stacked ^{1}H NMR spectra showing the ROP of ε-CL in the presence of 2 mol% **4** and 100 mol% *L*-LA.

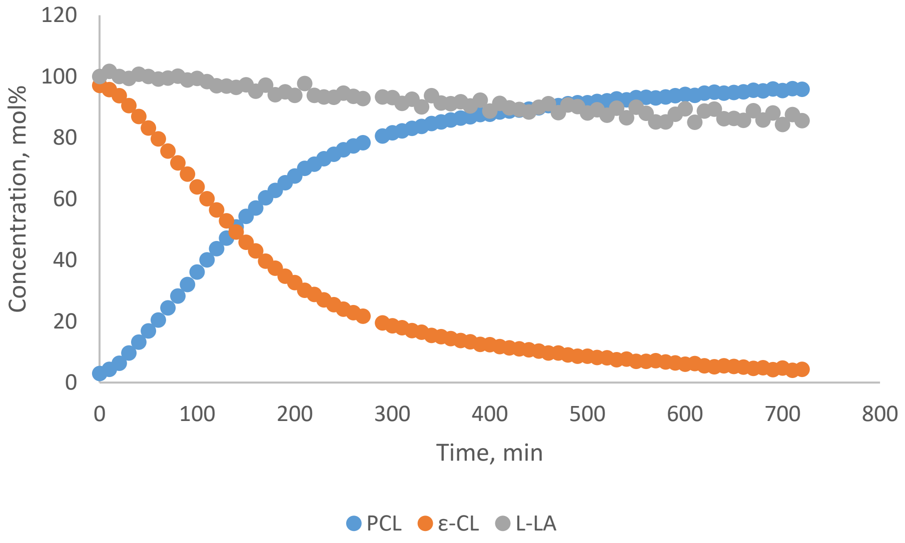

Figure S78. Plots of concentration *versus* time for the various monomer and polymer species present, where ε-CL was polymerized by **4** in the presence of 100 mol% *L*-LA

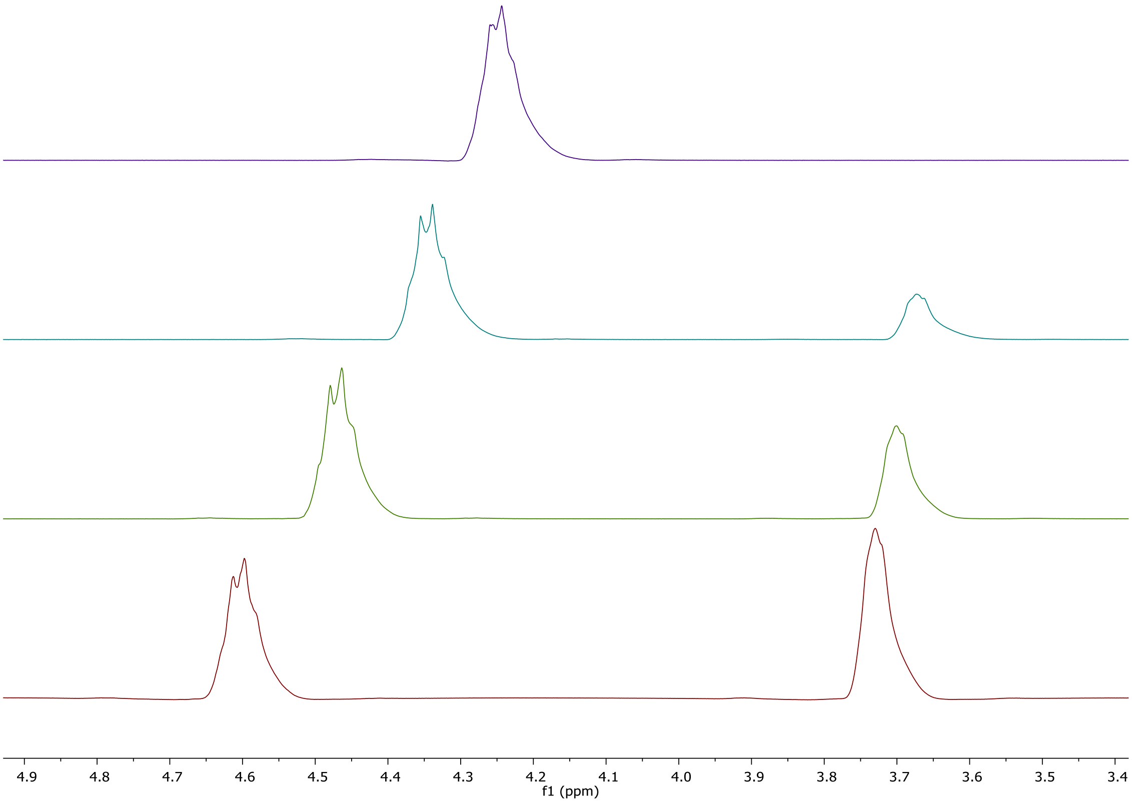

Figure S79. Stacked ^{1}H NMR spectra showing the titration of *L*-LA (δ = 4.25–4.6 ppm) against ε-CL (δ = 3.7 ppm) in toluene-d_8 at 80 °C.

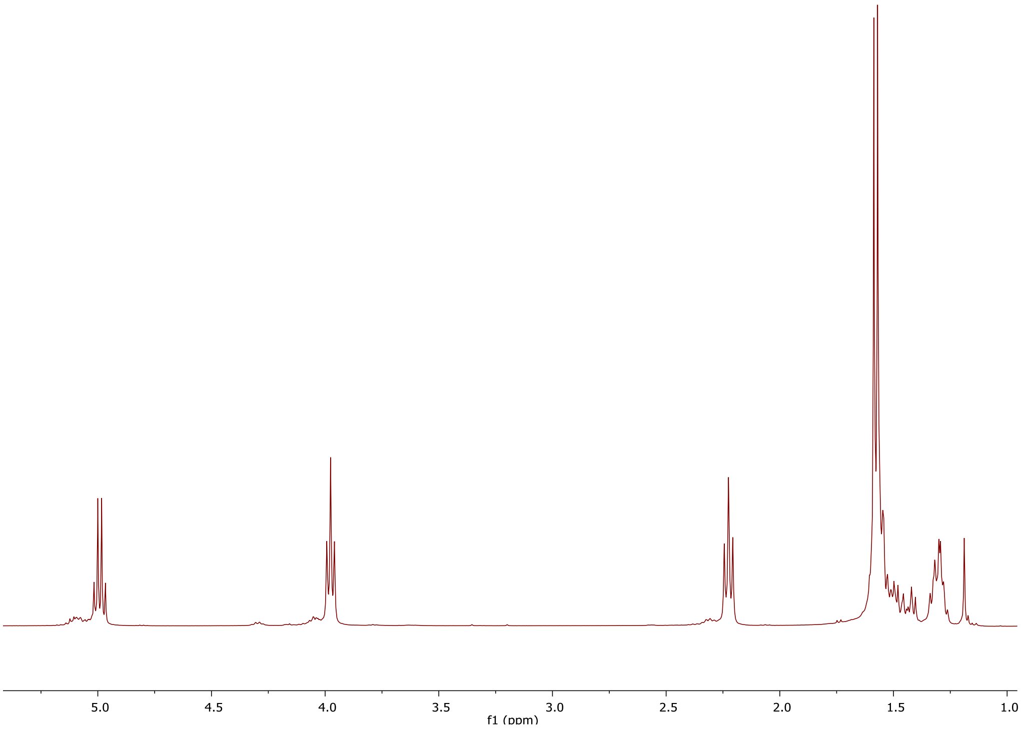

Figure S80. ^{1}H NMR spectrum of crude product mixture of ε-CL polymerization by **4** in the presence of *L*-LA, showing selective homopolymerization of ε-CL.

Polymerisation of THF

In the course of NMR solvent screening for the characterisation of species **3**, **4** and **7** we observed that THF-d_8 was polymerised at ambient temperature on addition of each respective cationic species. This reactivity was confirmed by adding 0.1 mol% of each species to *protio*-THF.

In the glove box, to 200 mg (200 µL) *protio*-THF was added 0.1 mol% of the relevant solid Nb complex. The mixture was sealed in a 1 ml vial and shaken vigorously until solvation of the solid catalyst occurred (<1 minute). Samples were then not stirred, in order to best replicate the conditions of the NMR samples.

Conversion was ascertained by ^{1}H NMR spectroscopy, by comparison of resonances at δ_H= 3.73 ppm and δ_H= 1.84 ppm, corresponding to the OCH_2 and OCH$_2$CH_2 protons of THF, respectively, and those at δ_H= 3.40 and δ_H= 1.61, corresponding to the OCH_2 and OCH$_2$CH_2 protons of poly(THF), respectively. The conversion given is the mean value determined from the OCH_2 and OCH$_2$CH_2 regions (Table S5, Figure S81). The polymers were not characterised further, and improved polymerisation conditions were not pursued, as the preparation of poly(THF) was not of relevance to the current mechanistic study of the ROP of cyclic esters. A control reaction was carried out in the presence of 1 mol% $AgSbF_6$, in order to confirm the relevance of the Nb-containing cationic species to the polymerisation of THF.

Table S5. Conversion data corresponding to the solvent-free polymerisation of THF in the presence of cationic Nb species

Entry	Pre-catalyst	[c]Conversion, %
1[a]	**3**	87
2[a]	**4**	85
3[a]	**7**	66
4[b]	$AgSbF_6$	0

Conditions: [a] 250 mg THF, solvent-free, 298 K, 18 hours, 0.1 mol% pre-catalyst. [b] 200 mg THF, solvent-free, 298 K, 18 hours, 1.0 mol% catalyst. [c] Conversion determined *via* ^{1}H NMR spectroscopy, by integration of the resonances at δ_H= 3.73 ppm and δ_H= 1.84 ppm, corresponding to the OCH_2 and OCH$_2$CH_2 protons of THF, respectively, and those at δ_H= 3.40 and δ_H= 1.61, corresponding to the OCH_2 and OCH$_2$CH_2 protons of poly(THF), respectively. The conversion given is the mean value determined from the OCH_2 and OCH$_2$CH_2 regions.

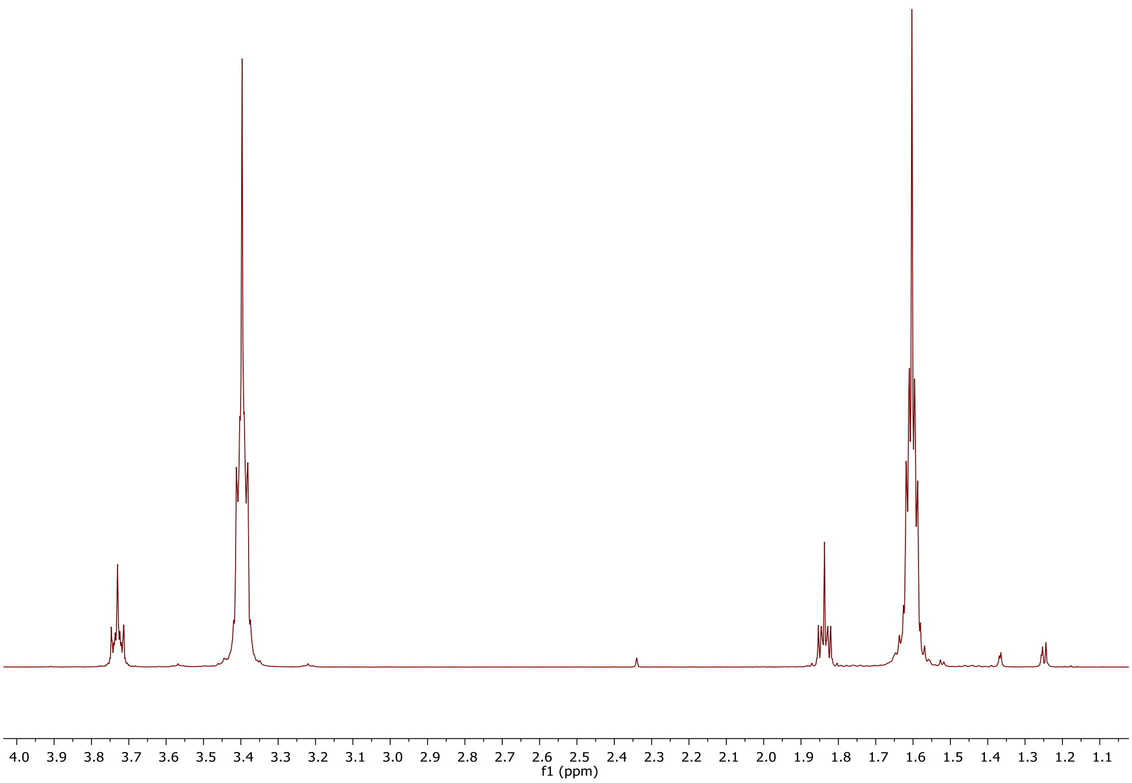

Figure S81. 1H NMR spectrum of crude reaction mixture from ROP of THF in the presence of Nb species **3**

Example gel permeation chromatograms

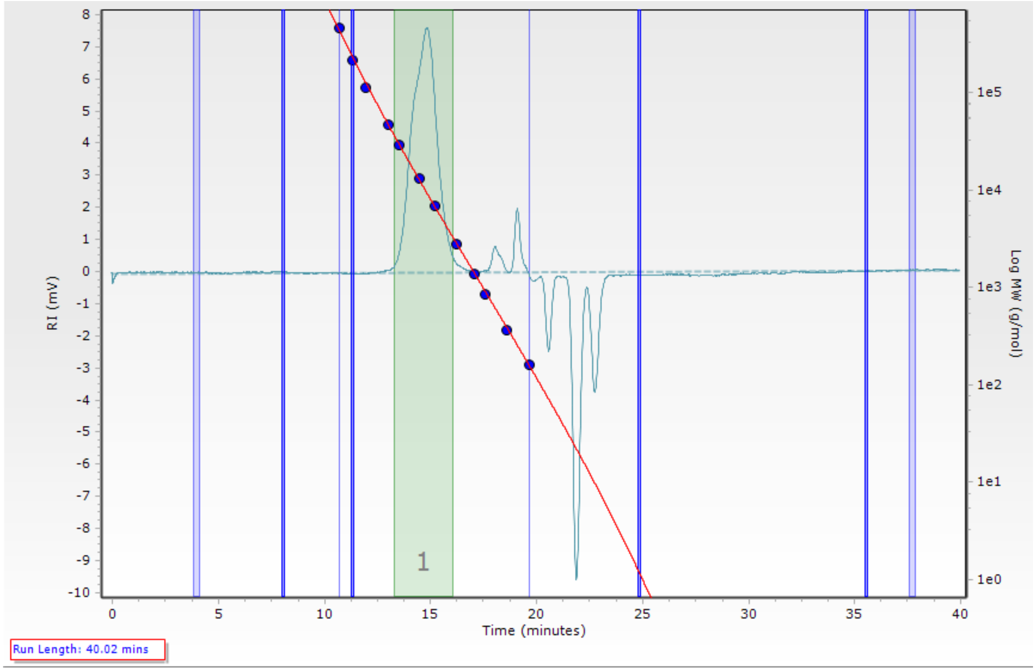

Figure S82. GPC trace, showing the refractive index (RI) data, for the PCL sample produced in the presence of pre-catalyst **3** (ε-CL:**3**:BnOH = 60:1:0) and calibration curve, from polystyrene standards.

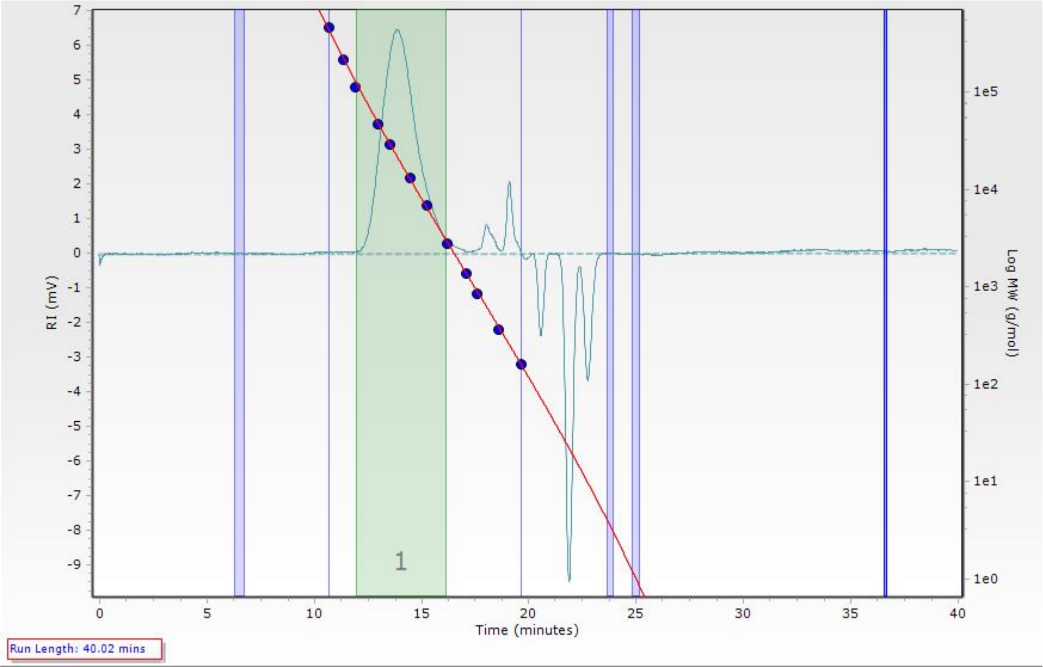

Figure S83. GPC trace, showing the refractive index (RI) data, for the PCL sample produced in the presence of catalyst **4** (ε-CL:**4**:BnOH = 60:1:0) and calibration curve, from polystyrene standards.

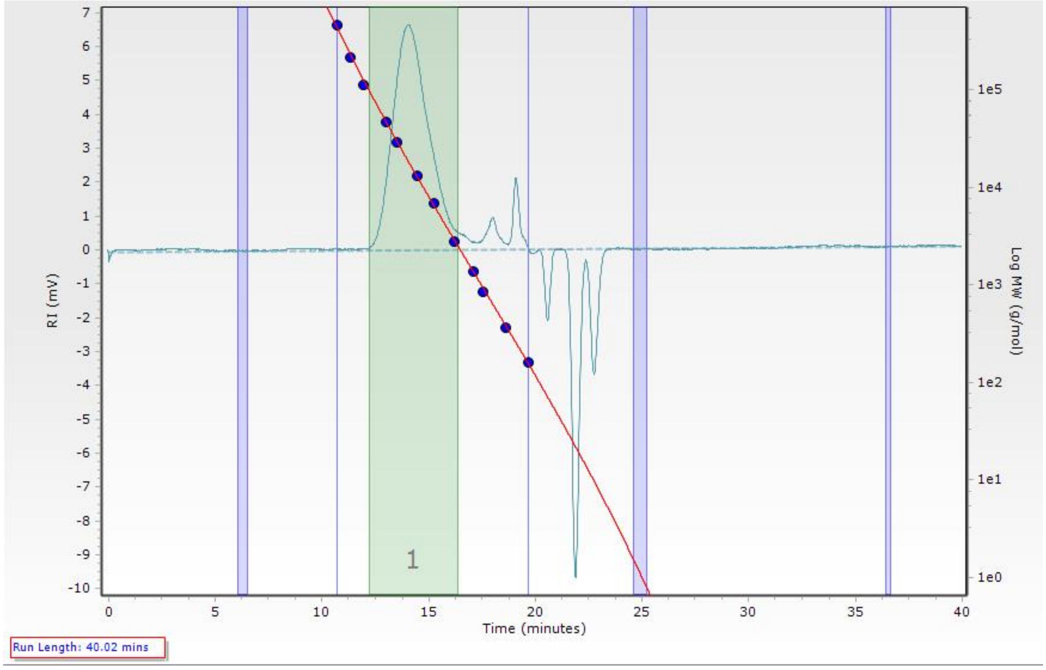

Figure S84. GPC trace, showing the refractive index (RI) data, for the PCL sample produced in the presence of pre-catalyst **7** (ε-CL:**7**:BnOH = 60:1:0) and calibration curve, from polystyrene standards.

Computational details

Density Functional theory (DFT) and Natural Bond Orbital (NBO) calculations were performed using Gaussian16 (revision A.03)[8] and NBO 3.1.[9] Geometries were fully optimised without any symmetry or geometry constraints. When the metal complex considered was cationic, the anion was omitted to prevent from arbitrarily assigning it a position in space. The nature of all the stationary points as minima or transition states (first-order saddle points) on the potential energy surface was verified by calculations of the vibrational frequency spectrum. Using the the GoodVibes program,[10] quasi-harmonic corrections for 353.15 K and 0.8 mol/L were applied to the computed free enthalpies using a frequency cut-off value of 100.0 cm^{-1}, according to the model proposed by Grimme.[11]

NBO calculations

Structures were optimized using the rωB97XD LC hybrid functional developed by Chai and Head-Gordon that includes an empirical dispersion correction,[12,13] using the 6-311+G(d) basis set for O and N atoms, 6-31G(d,p) for C and H atoms and the SDD pseudopotential and associated basis set for Nb. Solvent effects in toluene were considered using conductor-like polarisable continuum model (CPCM) at 298.15 K in toluene. Natural Bond Orbital (NBO) calculations were performed using the NBO 3.1 package included in Gaussian16.

Full coordinates for modelled complexes **1**, **2**, **4** and **4'**, together with computed Gibbs free energy and vibrational frequency and NBO data, are available via the corresponding Gaussian 16 output files, stored in the digital repository, DOI: 10.6084/m9.figshare.8299313..

Free enthalpy profile for the initiation step of ε-caprolactone ROP from 4

DFT optimisation was carried out using the rωB97XD LC hybrid functional developed by Chai and Head-Gordon that includes an empirical dispersion correction,[12,13] using the 6-311+G(d) basis set for O and N atoms, 6-31G(d,p) for C and H atoms and the SDD pseudopotential and associated basis set for Nb. Solvent effects in toluene were considered using conductor-like polarisable continuum model (CPCM).

Full coordinates for all the stationary points, together with computed Gibbs free energy and vibrational frequency data, are available via the corresponding Gaussian 16 output files, stored in the digital repository, DOI: 10.6084/m9.figshare.8299496..

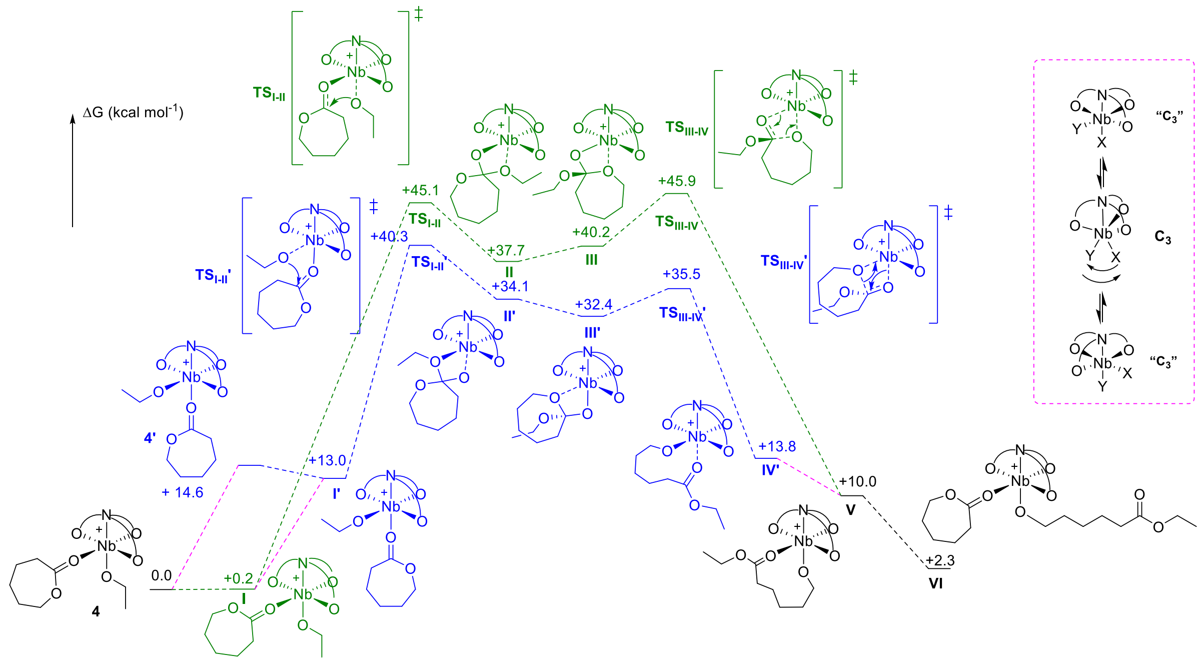

Figure S85. Free enthalpy profiles for the initiation step of ε-caprolactone ROP from **4** (same figure as Figure 5 in main article).

Table S6. Computed free Gibbs energies at the rωB97XD/6-311+g(d)/6-31g(d)/SDD/cpcm=toluene level of theory, corrected for 353.15 K and 0.8 mol L^{-1}, for the ring-opening of ε-caprolactone ROP from **4**

Structure	***G (Hartree)* (QH correction)**	***ΔG (kcal mol^{-1})***
ε-caprolactone	-384.932237	
**4**	-2629.912305	0.0
**I**	-2629.911979	+0.2
**TS$_{I-II}$**	-2629.840452	+45.1
**Alt-TS$_{I-II}$**	-2629.840424	+45.1
**II**	-2629.852256	+37.7
**III**	-2629.848249	+40.2
**TS$_{III-IV}$**	-2629.840424	+45.9
**Alt-TS$_{III-IV}$**	-2629.836451	+47.6
**V**	-2629.89638	+10.0
**VI**	-3014.840954	+2.3 (inc.+ ε-CL)
**4’**	-2629.889083	+14.6
**I’**	-2629.89162	+13.0
**TS$_{I-II}$’**	-2629.848104	+40.3
**Alt-TS$_{I-II}$’**	-2629.845894	+41.7
**II’**	-2629.857885	+34.1
**III’**	-2629.860652	+32.4
**TS$_{III-IV}$’**	-2629.855809	+35.5
**Alt-TS$_{III-IV}$’**	-2629.843106	+43.4
**IV’**	-2629.890369	+13.8

Using an otherwise identical protocol, DFT optimisations were also carried out using functionals M06-D3 and B3LYP-BJ for geometries **4**, **I**, **TS$_{I-II}$** and **4’**, **I’**, **TS$_{I-II}$’**, to consolidate the comparison between the two initiation pathways identified.

Full coordinates for all the stationary points, together with computed Gibbs free energy and vibrational frequency data, are available via the corresponding Gaussian 16 output files, stored in the digital repository, DOI: 10.6084/m9.figshare.8299496.

These calculations revealed good agreement between all functionals, with $\Delta G_{TSI-II} > \Delta G_{TSI-II'}$.

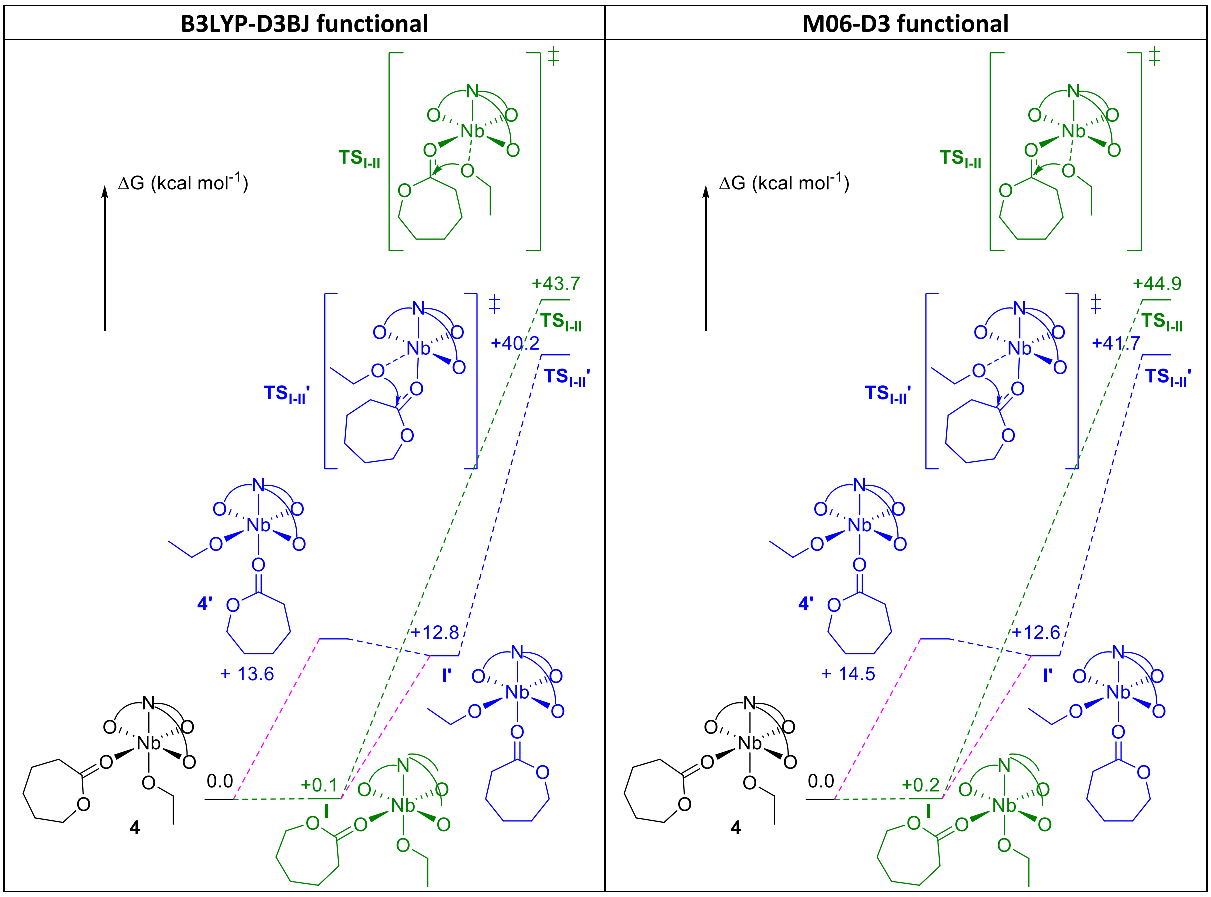

Figure S86. Partial free enthalpy profiles for the initiation step of ε-caprolactone ROP from **4**, using B3LYP-D3BJ (left) and M06-D3 functionals (right).

Table S7. Gibbs free energies computed using B3LYP-D3BJ or M06-D3 functional at the 6-311+g(d)/6-31g(d)/SDD/cpcm=toluene level of theory, corrected for 353.15 K and 0.8 mol L^{-1}, for the ring-opening of ε-caprolactone ROP from **4**

	B3LYP-D3BJ		***M06-D3***	
Structure	*G (Hartree)* (QH correction)	*ΔG (kcal mol^{-1})*	*G (Hartree)* (QH correction)	*ΔG (kcal mol^{-1})*
**4**	-2630.988405	0.0	-2628.767792	0.0
**I**	-2630.988248	+0.1	-2628.767399	+0.2
**TS$_{I-II}$**	-2630.918798	+43.7	-2628.696256	+44.9
**4’**	-2630.966711	+13.6	-2628.744717	+14.5
**I’**	-2630.967994	+12.8	-2628.747652	+12.6
**TS$_{I-II}$’**	-2630.924356	+40.2	-2628.701319	+41.7

Additional discussion of DFT results

While experimental evidence supports a coordination insertion mechanism, the true active catalyst remains uncertain. The facile isolation of **4**, and NBO calculations, collectively suggest that the proposed isomer **4’** should be more prone to initiating ROP *via* an insertion event than **4**. As described above, Density Functional Theory (DFT) was therefore also applied to model the initiation of ROP *via* insertion of the coordinated ε-CL molecule into the metal-alkoxide bond of complexes **4** and **4’**, permitting a qualitative comparison of the energetic accessibility of pathways proceeding with and without inversion, respectively, of the labile positions. $\Delta G^{\ddagger}$ values were evaluated at 353.15 K using a toluene solvent model and a concentration of 0.8 mol L^{-1}, corresponding to experimental conditions under which **4** is observed to be catalytically active. The most favorable Gibbs Free Energy profiles calculated for the initiation step are summarized in Figure S85 (see above for alternative transition states and full computational details, including links to output files).

Accordingly, two types of transition states were modelled, corresponding to nucleophilic attack at the monomer carbonyl group, and the subsequent ring-opening event. From **4’**, the nucleophilic attack at the monomer carbonyl group by the adjacent ethoxide moiety was found to be the limiting step, whereas from **4** the ring-opening event was rate-limiting. Figure S87 illustrates the various transition state structures calculated for this step, depending on the orientation of the ε-caprolactone molecule. For **4**, the large activation barrier for intramolecular nucleophilic attack ($\Delta G^{\ddagger}$ = +45.1 kcal mol^{-1}) is consistent with the observed stability of that species at ambient temperature, and with initiation of ROP becoming facile upon heating to 80 °C. While the Gibbs Free Energy of **4’** was calculated to be +14.6 kcal mol^{-1} higher than that of **4**, the subsequent nucleophilic attack by the alkoxide moiety on the adjacent monomer carbonyl group (**TS_{I-II}’**) was significantly more favorable in the case of **4’** than for **4** ($\Delta G^{\ddagger}$ = +40.3 kcal mol^{-1}; so 4.8 kcal mol^{-1} lower, and 5.6 kcal mol^{-1} lower than the rate-limiting ring-opening step, $\Delta G^{\ddagger}$ = +45.9 kcal mol^{-1}, in the calculated pathway from **4**), in line with previous NBO calculations.

Whilst the absolute Gibbs Free Energy values reported here appear unexpectedly high for a ROP process of this type (indeed, Transition State Theory indicates that $\Delta G^{\ddagger}$ should be in the approximate range 27 – 30 kcal mol^{-1}), there are several challenges intrinsic to modelling the current system. Firstly, all species considered are cationic, existing in the presence of a non-coordinating hexafluoroantimonate anion. Given the inherent ambiguity associated with determining the location of such a counterion, relative to the cationic fragment, this component of the system, which may play a significant role in stabilizing the various intermediates and transition states formed, was not accounted for in our calculations. Secondly, our computational study only considered the initiation event, rather than the propagation process that makes the vastly greater contribution to determining the experimentally observed activity of the system, but which is significantly more computationally demanding to model. Expanding our computational study, the relatively modest scope of which is intended only to assist in qualitatively elucidating possible mechanistic bases to support our experimental findings, to encompass propagation was, accordingly, beyond the scope of the current work. Moreover, although **4’** represents the only conceivable *pseudo*-octahedral isomer of **4** resulting from rearrangement about the Nb center, it is possible that propagation could follow a subtly distinct pathway, for example with variation of the coordination geometry of the active species.

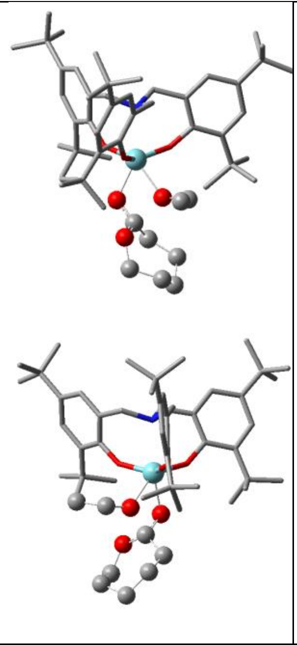	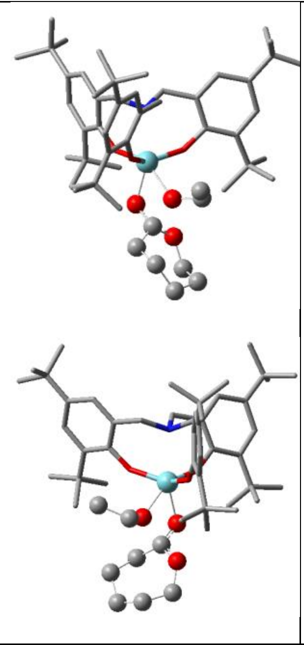	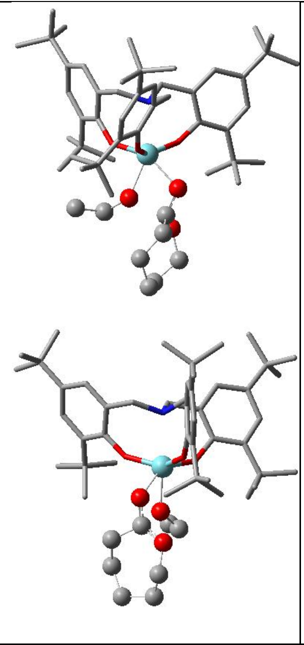	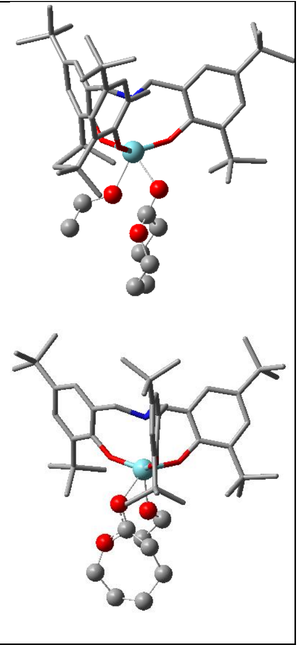
from **4’**	from **4’**	from **4**	from **4**
$\Delta G^{\ddagger}$ = +41.7 kcal mol^{-1}	$\Delta G^{\ddagger}$ = +40.3 kcal mol^{-1}	$\Delta G^{\ddagger}$ = +45.1 kcal mol^{-1}	$\Delta G^{\ddagger}$ = +45.1 kcal mol^{-1}

Figure S87. Various transition state structures for the nucleophilic attack at the ε-CL carbonyl group by the Nb-ethoxide bond of **4** and **4’**, and their Gibbs Free Energy relative to **4** (compound isolated experimentally) (rωB97XD/353.15K/cpcm=toluene/6-31(+)g(d)/6-311+g(d)/SDD protocol; all complexes are cationic and the counter anion is omitted).

Given the outlined difficulties associated with ascertaining absolute Gibbs Free Energy values for the various species and transition states modelled, the calculated values should be considered representative of the relative energetic favorability of the two pathways that appear to be most feasible for the current system. To ensure maximum validity in making such a comparison, the first structures of the initiation step, including the rate-determining transition states **TS_{I-II}** and **TS_{I-II}’**, were reoptimized using two additional functionals: M06-D3 and B3LYP-D3BJ (Figure S86, Table S7), which provided results consistent with those obtained using rωB97XD.

Crystallographic Parameters

Solid state structures for complexes **1** – **7** are represented by CCDC deposition numbers 2252657-2252664.

Table S7. Crystallographic Parameters for Complexes **1-7**

Compound reference	**1**	**2**	**3**	**3a**	**4**	**5**	**6**	**7**
Chemical formula	$C_{399}H_{616}N_8Nb_8O_{40}$	$C_{51}H_{79}ClNNbO_5$	$C_{108.80}H_{156.20}F_8N_2Nb_2O_7Sb$	$C_{103.50}H_{147}F_9N_2Nb_2O_6Sb$	$C_{65}H_{93}F_6NNbO_6Sb$	$C_{31}H_{40}NNbO_5$	$C_{33.40}H_{41.40}ClNNbO_{4.40}$	$C_{65}H_{78}F_7N_2Nb_2O_8Sb$
Formula Mass	6908.28	914.51	2063.72	1993.79	1313.06	599.55	655.63	1455.86
Crystal system	Tetragonal	Monoclinic	Monoclinic	Monoclinic	Monoclinic	Orthorhombic	Monoclinic	Monoclinic
a/Å	31.71100(10)	17.2143(3)	22.9621(6)	22.8756(9)	13.3706(4)	23.4940(2)	17.3465(12)	13.5593(4)
b/Å	31.71100(10)	10.6849(2)	19.2799(3)	19.2700(3)	27.1212(10)	9.45640(10)	19.6497(8)	22.6077(6)
c/Å	9.88450(10)	28.0303(5)	27.1056(6)	27.8865(11)	18.6887(5)	26.2189(2)	9.6027(5)	21.5667(8)
α/°	90	90	90	90	90	90	90	90
β/°	90	99.200(2)	112.479(3)	115.752(5)	104.767(3)	90	96.443(5)	103.440(3)
γ/°	90	90	90	90	90	90	90	90
Unit cell volume/Å^3	9939.73(12)	5089.38(16)	11088.1(5)	11071.8(8)	6553.2(4)	5825.02(9)	3252.4(3)	6430.1(4)
Temperature/K	150(2)	150(2)	150(2)	150(2)	150(2)	150(2)	150(2)	150(2)
Space group	*P4/n*	*P21/c*	*C2/c*	*C2/c*	*P21/n*	*Pbca*	*P21/c*	*P21/n*
No. of formula units per unit cell, Z	1	4	4	4	4	8	4	4
Radiation type	Cu Kα	Cu Kα	Mo Kα	Mo Kα	Mo Kα	Cu Kα	Cu Kα	Cu Kα
Absorption coefficient, μ/mm^{-1}	2.296	2.741	0.509	0.508	0.652	3.682	4.068	6.797
No. of reflections measured	76026	38491	65622	70436	74006	43236	27209	50479
No. of independent reflections	9887	9646	12900	12580	14427	5527	6321	12220
R_{int}	0.0428	0.0725	0.0503	0.0425	0.0555	0.0323	0.0813	0.0645
Final R_1 values ($I > 2\sigma(I)$)	0.0359	0.0454	0.0557	0.0643	0.0558	0.0279	0.0775	0.0478
Final $wR(F^2)$ values ($I > 2\sigma(I)$)	0.0947	0.1134	0.1286	0.1525	0.0988	0.0710	0.1770	0.1144
Final R_1 values (all data)	0.0400	0.0514	0.0868	0.0869	0.0755	0.0299	0.1070	0.0675
Final $wR(F^2)$ values (all data)	0.0973	0.1182	0.1421	0.1642	0.1049	0.0724	0.1934	0.1254

References

(1) Groysman, S.; Segal, S.; Shamis, M.; Goldberg, I.; Kol, M.; Goldschmidt, Z.; Hayut-Salant, E. Tantalum(v) Complexes of an Amine Triphenolate Ligand: A Dramatic Difference in Reactivity between the Two Labile Positions. *J. Chem. Soc. Dalt. Trans.* **2002**, *0* (18), 3425–3426. https://doi.org/10.1039/b206759e.

(2) Lewiński, J.; Horeglad, P.; Wójcik, K.; Justyniak, I. Chelation Effect in Polymerization of Cyclic Esters by Metal Alkoxides: Structure Characterization of the Intermediate Formed by Primary Insertion of Lactide into the Al-OR Bond of an Organometallic Initiator. *Organometallics* **2005**, *24* (19), 4588–4593. https://doi.org/10.1021/om050295v.

(3) Chumsaeng, P.; Haesuwannakij, S.; Bureekaew, S.; Ervithayasuporn, V.; Namuangruk, S.; Phomphrai, K. Polymerization of ε-Caprolactone Using Bis(Phenoxy)-Amine Aluminum Complex: Deactivation by Lactide. *Inorg. Chem.* **2018**. https://doi.org/10.1021/acs.inorgchem.8b01364.

(4) Delle Chiaie, K. R.; Biernesser, A. B.; Ortuño, M. A.; Dereli, B.; Iovan, D. A.; Wilding, M. J. T.; Li, B.; Cramer, C. J.; Byers, J. A. The Role of Ligand Redox Non-Innocence in Ring-Opening Polymerization Reactions Catalysed by Bis(Imino)Pyridine Iron Alkoxide Complexes. *Dalt. Trans.* **2017**, *46* (38), 12971–12980. https://doi.org/10.1039/c7dt03067c.

(5) Nomura, N.; Akita, A.; Ishii, R.; Mizuno, M. Random Copolymerization of ε-Caprolactone with Lactide Using a Homosalen-Al Complex. *J. Am. Chem. Soc.* **2010**, *132* (6), 1750–1751. https://doi.org/10.1021/ja9089395.

(6) Gontard, G.; Amgoune, A.; Bourissou, D. Ring-Opening Polymerization of ε-Caprolactone Catalyzed by Ionic Hydrogen Bond Activation with Bis-Pyridiniums. *J. Polym. Sci. Part A Polym. Chem.* **2016**, *54* (20), 3253–3256. https://doi.org/10.1002/pola.28238.

(7) Save, M.; Schappacher, M.; Soum, A. Controlled Ring-Opening Polymerization of Lactones and Lactides Initiated by Lanthanum Isopropoxide, 1 General Aspects and Kinetics. *Macromol. Chem. Phys.* **2002**, *203* (5–6), 889–899. https://doi.org/10.1002/1521-3935(20020401)203:5/6<889::AID-MACP889>3.0.CO;2-O.

(8) Frisch, M. J.; Trucks, G. W.; Schlegel, H. B.; Scuseria, G. E.; Robb, M. A.; Cheeseman, J. R.; Scalmani, G.; Barone, V.; Petersson, G. A.; Nakatsuji, H.; Li, X.; Caricato, M.; Marenich, A. V.; Bloino, J.; Janesko, B. G.; Gomperts, R.; Mennucci, B.; Hratchian, H. P.; Ortiz, J. V.; Izmaylov, A. F.; Sonnenberg, J. L.; Williams-Young, D.; Ding, F.; Lipparini, F.; Egidi, F.; Goings, J.; Peng, B.; Petrone, A.; Henderson, T.; Ranasinghe, D.; Zakrzewski, V. G.; Gao, J.; Rega, N.; Zheng, G.; Liang, W.; Hada, M.; Ehara, M.; Toyota, K.; Fukuda, R.; Hasegawa, J.; Ishida, M.; Nakajima, T.; Honda, Y.; Kitao, O.; Nakai, H.; Vreven, T.; Throssell, K.; Montgomery, J. A., J.; Peralta, J. E.; Ogliaro, F.; Bearpark, M. J.; Heyd, J. J.; Brothers, E. N.; Kudin, K. N.; Staroverov, V. N.; Keith, T. A.; Kobayashi, R.; Normand, J.; Raghavachari, K.; Rendell, A. P.; Burant, J. C.; Iyengar, S. S.; Tomasi, J.; Cossi, M.; Millam, J. M.; Klene, M.; Adamo, C.; Cammi, R.; Ochterski, J. W.; Martin, R. L.; Morokuma, K.; Farkas, O.; Foresman, J. B.; Fox, D. J. Gaussian 16, Revision. A.03. *Gaussian, Inc., Wallingford, CT*. 2016.

(9) Glendening, E. D.; Badenhoop, J. K.; Reed, A. D.; Carpenter, J. E.; Weinhold, F. NBO 3.1. *Theor. Chem. Institute, Univ. Wisconsin, Madison, WI* **1996**.

(10) Funes-Ardoiz, I.; Paton, R. S. GoodVibes: Version 2.0.3. **2018**. https://doi.org/10.5281/ZENODO.1435820.

(11) Grimme, S. Supramolecular Binding Thermodynamics by Dispersion-Corrected Density Functional Theory. https://doi.org/10.1002/chem.201200497.

(12) Chai, J. Da; Head-Gordon, M. Optimal Operators for Hartree-Fock Exchange from Long-Range Corrected Hybrid Density Functionals. *Chem. Phys. Lett.* **2008**, *467* (1–3), 176–178. https://doi.org/10.1016/j.cplett.2008.10.070.

(13) Chai, J. Da; Head-Gordon, M. Long-Range Corrected Hybrid Density Functionals with Damped Atom-Atom Dispersion Corrections. *Phys. Chem. Chem. Phys.* **2008**, *10* (44), 6615–6620. https://doi.org/10.1039/b810189b.
